# Stereoselective Activation of Small Molecules by a Stable Chiral Silene

**DOI:** 10.1002/chem.202201963

**Published:** 2022-08-03

**Authors:** Xiaofei Sun, Alexander Hinz, Hannes Kucher, Michael T. Gamer, Peter W. Roesky

**Affiliations:** ^1^ Institute of Inorganic Chemistry Karlsruhe Institute of Technology (KIT) Engesserstraße 15 76131 Karlsruhe Germany

**Keywords:** group 14, silene, silicon, small molecule activation, stereoselectivity

## Abstract

The reaction of the dilithium salt of the enantiopure (*S*)‐BINOL (1,1’‐bi‐2‐naphthol) with two equivalents of the amidinate‐stabilized chlorosilylene [L^Ph^SiCl] (L^Ph^=PhC(N*t*Bu)_2_) led to the formation of the first example of a chiral cyclic silene species comprising an (*S*)‐BINOL ligand. The reactivity of the Si=C bond was investigated by reaction with elemental sulfur, CO_2_ and HCl. The reaction with S_8_ led to a Si=C bond cleavage and concomitantly to a ring‐opened product with imine and silanethione functional groups. The reaction with CO_2_ resulted in the cleavage of the CO_2_ molecule into a carbonyl group and an isolated O atom, while a new stereocenter is formed in a highly selective manner. According to DFT calculations, the [2+2] cycloaddition product is the key intermediate. Further reactivity studies of the chiral cyclic silene with HCl resulted in a stereoselective addition to the Si=C bond, while the fully selective formation of two stereocenters was achieved. The quantitative stereoselective addition of CO_2_ and HCl to a Si=C bond is unprecedented.

## Graphical abstract

**A novel cyclic** and chiral silene was synthesized and fully characterized. The chiral (*S*)‐BINOL scaffold in the silene enabled unprecedented complete control over the diastereoselectivity in the reactions with different small molecules, S_8_, CO_2_ and HCl.

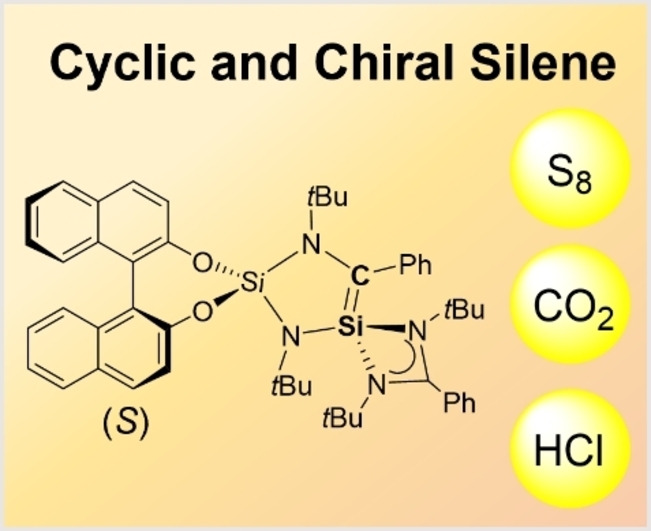

## Introduction

Many basic chemical processes such as catalysis rely on the bond cleavage of ubiquitous small molecules as the initial step for further transformations. For several decades, the activation of small molecules has been dominated by transition‐metal systems. Over the last two decades, the chemistry of low‐valent main group species has become more mature and as main group compounds that mimic the behavior of transition‐metal complexes have developed significantly, remarkable progress has been made in the field of activating small molecules.^[1]^ The utilization of readily available small molecules as convenient building blocks for the preparation of useful functional compounds is of particular interest, moreover, as molecules comprising multiple functional entities can enable application potential in material science. Extensive studies showed that diverse main group element compounds, including multiple‐bonded species,^[2]^ frustrated Lewis pairs (FLPs),^[3]^ carbenes and their heavier congeners^[4]^ can activate simple small molecules such as H_2_, CO, CO_2_ and N_2_O. Considering environmental and economic aspects, the quest for bond activation by earth abundant main group species is an avenue worth pursuing. Development of novel main group element systems for the potential use of small molecule activation is the aim of many synthetic chemists.

Heavier group 14 alkene analogues have unique properties and reactivities distinguishing them from their parent organic species. The synthesis and characterization of compounds comprising silicon‐carbon double bonds was first realized in 1981 when Brook isolated the first stable silene [(Me_3_Si)_2_Si=C(OSiMe_3_)Ad] (Ad=1‐adamantyl).^[5]^ Over the years different pathways to access silenes have been established, most of them are acyclic molecules. More recently, novel cyclic silenes have been established.^[6]^ Due to lack of suitable synthetic methodology, examples of cyclic silene molecules are rather rare, most of them have been synthesized through different unexpected exotic pathways. For instance, the reaction between an amidosilylene with a phosphaalkyne resulted in the formation of a five‐membered heterocyclic silene.^[6a]^ Due to the polarity difference between carbon and silicon, silenes readily react with various nucleophiles.^[7]^ In addition, the use of chiral low‐valent main‐group‐based species has received considerable attention in the recent years, as an example, chiral phosphines and carbenes find broad applications in asymmetric catalysis.^[8]^ In our work, we focused on generating a low‐valent group 14 species with a chiral ligand scaffold. For that purpose, (*S*)‐BINOL (1,1’‐bi‐2‐naphthol) was chosen as a chiral precursor as it is commercially available, cheap, and easy to doubly deprotonate with *n*‐BuLi.

Herein, we report the synthesis of the cyclic silene [BINO−Si(N*t*Bu)_2_(Ph)C=Si(L^Ph^)] (**1**) (L^Ph^=PhC(N*t*Bu)_2_) comprising a chiral (*S*)‐BINOL backbone. Compound **1** features an excellent combination of high thermal stability and high reactivity towards S_8_, CO_2_ and HCl. Due to the presence of the chiral (*S*)‐BINOL moiety, the reactions with the corresponding molecules occurred in highly stereoselective pathways and thus, exclusively produced a single activation product.

## Results and Discussion

**Scheme 1:**
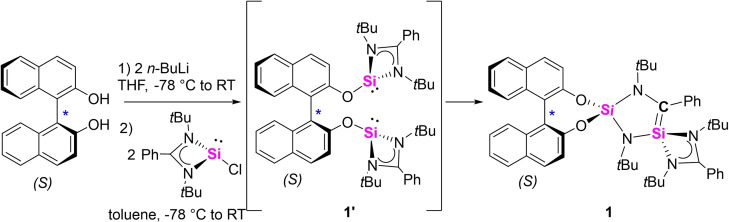
Synthesis of the chiral silene **1**.

Deprotonation of (*S*)‐BINOL with two equivalents *n*‐BuLi in THF and subsequent reaction with two equivalents of *N*,*N*’‐di‐*tert*‐butyl(phenylamidinato)‐chlorosilylene [L^Ph^SiCl]^[9]^ (L^Ph^=PhC(N*t*Bu)_2_) led to the formation of a dark brown solution (Scheme 1). After extraction with toluene, the cyclic silene [BINO−Si(N*t*Bu)_2_(Ph)C=Si(L^Ph^)] (**1**) could be obtained in 69 % yield as orange crystalline solid. The molecular structure of **1** was established by multinuclear (^1^H, ^13^C and ^29^Si) NMR spectroscopy and single crystal X‐ray diffraction analysis. Compound **1** crystallized in the non‐centrosymmetric space group *P*2_1_ with a low Flack parameter,^#^ which indicated the expected retention of the chiral axis.

**Figure 1:**
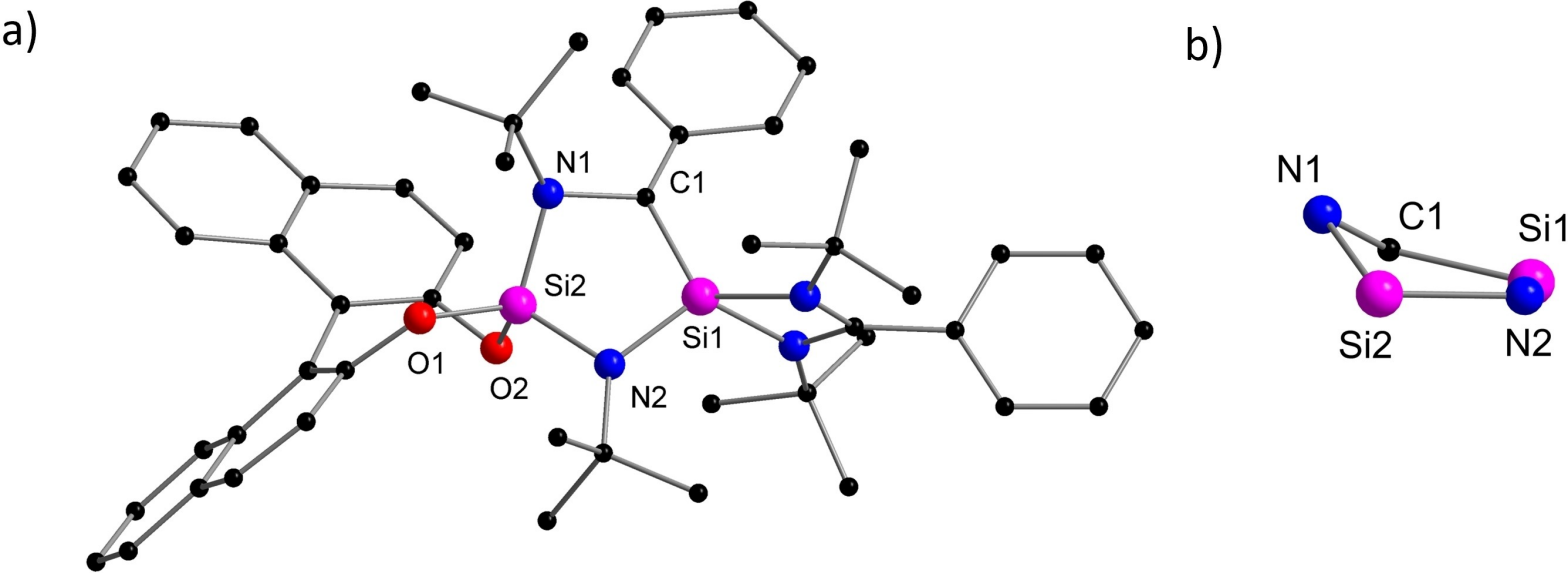
a) Molecular structure of compound **1** in the solid state, hydrogen atoms and non‐coordinating solvent molecules are omitted for clarity. Selected bond distances [Å] and angles [°]: Si1−C1 1.759(3), Si1−N2 1.719(2), C1−N1 1.488(4), Si2−N1 1.699(2), Si2−N2 1.737(3), Si2−O1 1.655(2), Si2−O2 1.655(2); S1−C1−N1 114.4(2), C1−N1−Si2 102.7(2), N1−Si2−N2 107.43(13), Si2−N2−Si1 102.61(12), N2−Si1−C1 102.22(14), O1−Si2−O2 104.11(11). b) side view of the slightly puckered Si_2_N_2_C ring.

The molecular structure confirmed the formation of a cyclic silene (Figure 1, a). Even though the exact mechanism is uncertain, we assume that **1** is formed via a bis(silylene) intermediate **1’** and subsequent insertion of one silylene unit into the C−N*t*Bu bond of the other silylene. Two crystallographically independent molecules were found in the asymmetric unit with similar metric parameters, therefore, the following discussion is restricted to only one of them. The central motif of **1** is composed of a slightly puckered five‐membered Si_2_N_2_C heterocycle (Figure 1, b). The two different Si atoms Si1 and Si2 are both in a distorted tetrahedral geometry but surrounded by strikingly distinct chemical environments. Si2 is coordinated by two amide nitrogen atoms and two oxygen atoms of the chiral (*S*)‐BINOL ligand. In turn, Si1 is coordinated by the amidinato moiety, one amide nitrogen (N2) and the carbon atom C1 with a Si−C distance of 1.759(3) Å. The Si1−C1 bond distance in **1** is much shorter than typical Si−C single bonds (1.86‐1.93 Å),^[10]^ but well comparable to that of Brook's silene [(Me_3_Si)_2_Si=C(OSiMe_3_)Ad] (1.764(3) Å),^[5]^ which suggests the presence of a Si=C double bond. Another noticeable structural feature is the relatively planar geometry around the tricoordinate C1 atom (∑C_1_=356.8°), indicating a sp^2^‐hybridized carbon atom. In addition, the Si=C double bond length in compound **1** is only marginally longer to that of another P‐containing heterocyclic silene (1.7536(3) Å),^[6a]^ in which the silene silicon atom is coordinated by the same amidinate substituent. Cyclic silenes are less studied than acyclic ones, although there are examples of cyclic silenes, to the best of our knowledge, compound **1** in which the Si=C double bond is incorporated in a five‐membered ring, is the first structurally characterized chiral cyclic silene.

The ^1^H NMR spectrum of **1** shows four singlets at *δ* 1.11, 1.20, 1.36 and 1.52 ppm for the four non‐equivalent *t*Bu substituents. The ^29^Si{^1^H} NMR spectrum displays two singlets at *δ* −27.5 (Si=C) and −61.3 ppm (OSiO). The silene silicon signal is comparable to that of the disilapentalene (*δ* −18.9 ppm)^[6e]^ and the phosphine‐oxide‐stabilized silene (*δ* −18.7 ppm),^[11]^ but at a much higher field than the sp^2^ silicon signals found in most of the 1,1‐disilyl‐substituted silenes (Me_3_Si)_2_Si=CR_2_ (*δ* 41–54 ppm).^[12]^ The ^13^C resonance of the unsaturated carbon nucleus appeared at *δ* 58.2 ppm, considerably upfield‐shifted compared to Brook's silene (*δ* 214.2 ppm).

When an NMR tube containing a C_6_D_6_ solution of **1** was heated at 80 °C for one week, no decomposition occurred. Although the silene **1** is very stable in the solid state as well as in solution under inert atmosphere, it was found to be highly reactive. Due to the polarization of the Si^δ+^=C^δ−^ bond, silenes are highly unstable species and have ambiphilic reactivity. Previous work has shown that typical reactions are cycloadditions with π‐bonded species or insertion reactions with polarized compounds.[^7^, ^12^, ^13^]

In an early report in 1994, Brook described that when they attempted to add sulfur across the double bonds of silenes of the type [(SiMe_3_)_2_Si=C(OSiMe_3_)R], very complex mixtures were obtained. Only in one instance when a silyl group on the silicon atom was exchanged by a mesityl group, the simple addition product, a three‐membered silathiirane (SiCS‐ring), could be isolated.^[14]^ To test the reaction of silene **1** with S_8_, the NMR‐scale reaction between the two compounds (1 : 0.14 molar ratio) was carried out in C_6_D_6_ (see Supporting Information, Figure S35). And indeed, an unexpected and rather complicated reaction occurred.

**Scheme 2:**
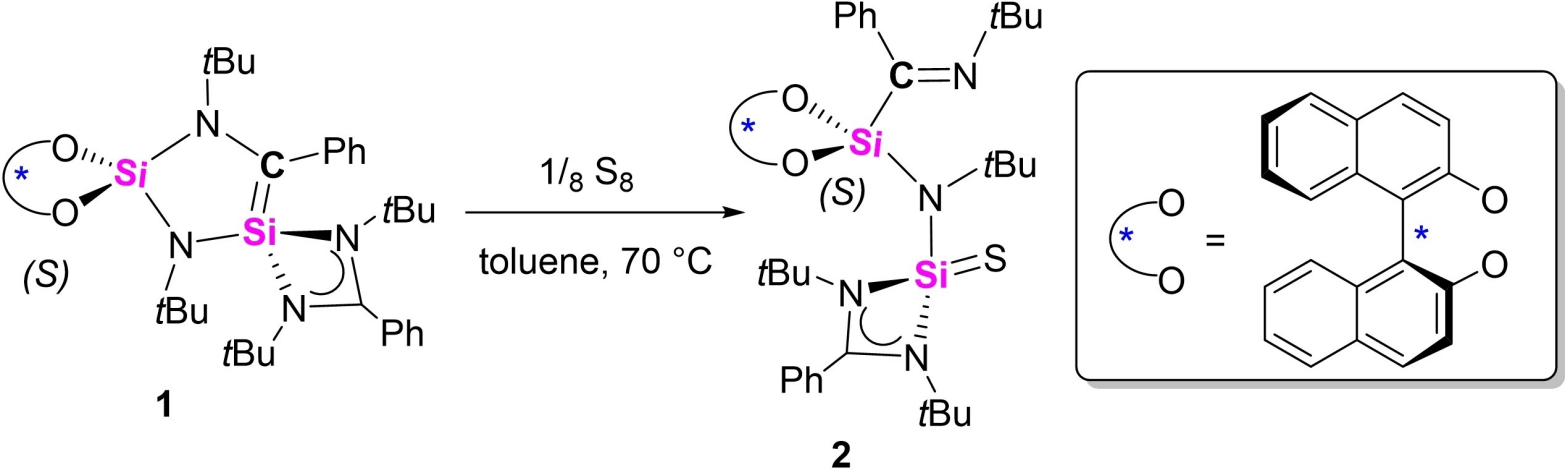
Formation of compound **2** by oxidation of **1** with S_8_.

**Figure 2:**
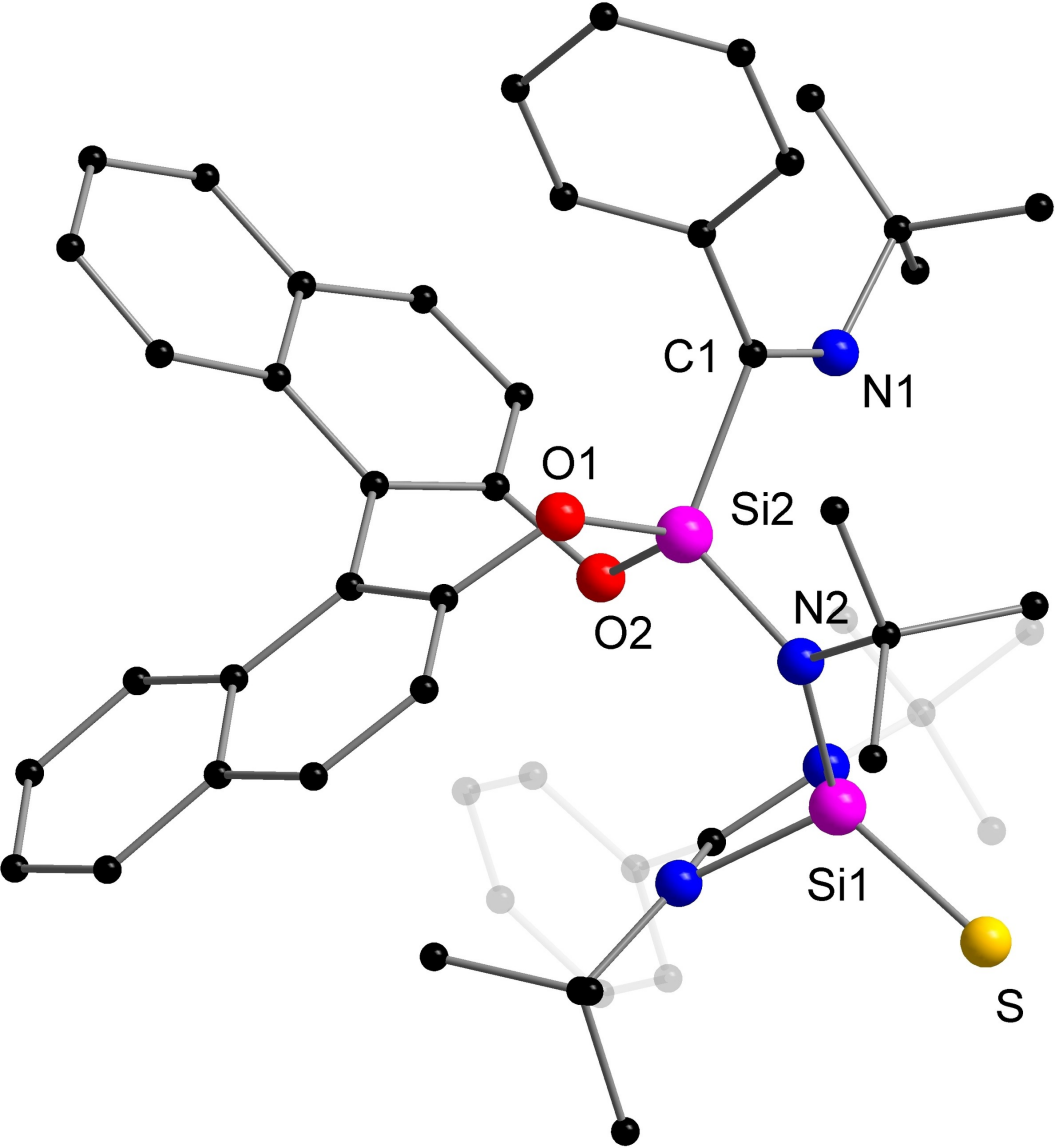
Molecular structure of compound **2** in the solid state, hydrogen atoms are omitted for clarity. Selected bond distances [Å] and angles [°]: Si1−S 2.0006(10), Si1−N2 1.753(2), Si2−C1 1.900(3), Si2−N2 1.720(2), Si2−O1 1.654(2), Si2−O2 1.662(2), C1−N1 1.276(4); N1−C1−Si2 110.0(2), O1−Si2−O2 103.85(10), C1−Si2−N2 117.72(12), Si2−N2−Si1 125.66(13), N2−Si1−S 123.38(8).

Of the mixture, compound **2** could be crystallized from toluene at room temperature as colorless crystals in 38 % yield (Scheme 2). The molecular structure of **2** determined by X‐ray analysis is displayed in Figure 2. Interestingly, the addition reaction with sulfur results in the cleavage of the Si=C double bond and a rearrangement to a newly formed imine moiety (C1=N1=1.273(4) Å). Simultaneously, a silanethione (Si=S) functional group is formed, the Si=S double bond length of 2.0006(10) Å is comparable to those in other amidinato‐silanethiones, such as [L^Ph^Si(=S)S*t*Bu] (1.984(8) Å),^[15]^ [L^Ph^Si(=S)Si(SiMe_3_)_3_] (1.9996(6) Å)^[6c]^ and [L^Ph^Si(=S)N(SiMe_3_)_3_] (1.984(8) Å),^[16]^ and related silanethione‐stabilized group 12 metal complexes (2.00–2.02 Å).^[17]^ A common route to access silanethiones was developed in the past by treatment of silylenes with elemental sulfur. The reaction of silene **1** with sulfur provided a novel route for the preparation of a chiral compound, which comprises two newly generated functional groups (imine and silanethione).

The NMR data of the isolated single crystals of **2** clearly suggests the co‐existence of two closely related isomers in solution. In C_6_D_6_, the two sets of *t*Bu protons (major isomer: *δ* 2.39, 1.75, 1.19, 0.56 ppm; minor isomer: 1.65, 1.41, 1.35, 1.35 ppm) are found in 4 : 3 molar ratio. This is in accordance with its ^29^Si{^1^H} NMR spectrum in which four signals were detected due to the presence of two species (*δ* −14.0, −20.2, −56.1 and −64.8 ppm). Changing the solvent from the aromatic C_6_D_6_ to the non‐aromatic but more polar CDCl_3_, the molar ratio of the two isomers varied from 4 : 3 to 9 : 4. The corresponding ^29^Si signals are only marginally shifted compared with those in C_6_D_6_. The signal for the major isomer was obtained at *δ* −13.1 and −56.6 ppm and those for the minor isomer at *δ* −20.0 and −64.8 ppm. The assignment of the conformation obtained by XRD being the major species in solution was corroborated by DFT calculations (Gaussian16/PBE0/def2‐SVP), as the calculated NMR parameters of **2** obtained by XRD are in good agreement with the experimental values of the major isomer (see Supporting Information). For the minor signals, we considered several isomers until assigning **2’** as a rotamer of **2**, where the imine moiety is rotated around the Si−C bond by 180°. It is reasonable to assume the rotation around this bond to be hindered, as all involved *t*Bu and Ph substituents are in close proximity. The characteristic upfield‐shifted ^1^H NMR resonances for two of the aromatic protons are accurately reproduced by the computation and result from the orientation of the imine‐phenyl group, which causes one *ortho* and one *meta* H to point into the aromatically shielded region of the BINOL moiety. High temperature ^1^H NMR studies indicate free rotation of the imine‐phenyl group but no interconversion of the two isomers (see Supporting Information, Figure S23–25).

The reaction leading to **2** was monitored by ^29^Si NMR spectroscopy and the results were interpreted by using DFT methods. Details are outlined in the Supporting Information and only briefly summarized here. Complete consumption of the starting materials occurred after 10 min reaction. Four new singlet signals (*δ* −28.0, −47.8, −52.4 and −107.3 ppm) appeared in the ^29^Si{^1^H} NMR spectrum, indicative of the formation of two new compounds. We suggested that sulfur was added in the first step to the Si=C bond forming two silaazirane isomers. These signals slowly disappear and a new transient intermediate, which is the result of the silaazirane ring opening, was seen. Thermally driving the reaction to completion resulted finally in **2** and **2’** as stable products.

**Scheme 3:**
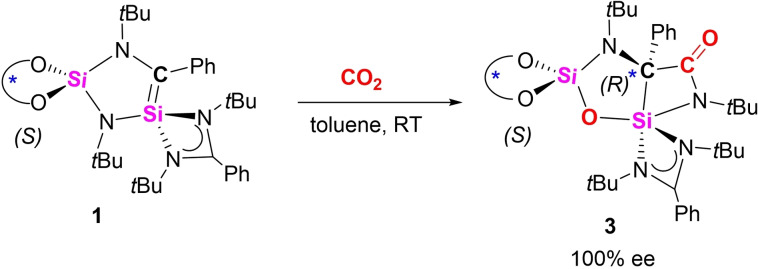
Formation of compound **3** by reaction of **1** with CO_2_.

The efficient capture and utilization of CO_2_ has been a great scientific challenge over the years. Synthetic chemists have developed different strategies to utilize this greenhouse gas as useful C1 building blocks. Compared to transition‐metal‐based systems, the reaction and conversion of CO_2_ with main group species are underexplored. Prominent examples in main group chemistry are FLPs, which combine nucleophilic and electrophilic centers and thus can form the corresponding CO_2_ insertion products.[^3c^, ^18^] Another possible mechanism is the [2+2] cycloaddition with polar multiple bond systems.^[19]^ Recently, the activation of CO_2_ by low valent silicon species especially silylenes has undergone intensive research. In most cases, CO_2_ acts as a O transfer reagent and releases CO.^[20]^ In contrast, the reactivity between CO_2_ and silenes remains much less explored. Kato and Baceiredo reported the [2+2] cycloaddition of the phosphine‐oxide‐stabilized silene with CO_2_.^[11]^ Very recently, Driess reported the CO_2_ insertion and fixation by a disilapentalene.^[6e]^ Intrigued by these examples, we started to explore the reaction of our cyclic chiral silene with CO_2_. Stirring a toluene solution of silene **1** under CO_2_ atmosphere at room temperature led to the decoloration of the solution (Scheme 3). After stirring for 48 h, the solvents were removed and the crude product **3** was obtained. Recrystallization led to colorless crystals of **3** in 34 % yield as single crystalline material. Due to loss upon crystallization relative low yields of crystalline material were observed. Monitoring the reaction by ^1^H NMR spectroscopy revealed slow formation of signals for only one set of *t*Bu groups (*δ* 1.82, 1.41, 1.11, 0.60 ppm), which hinted the quantitative and selective formation of only a sole reaction product **3** and there was spectroscopic evidence for neither an intermediate nor for the formation of a second diasteromer. Thus, **3** is the result of a stereoselective activation of CO_2_ with quantitative ee.

**Figure 3:**
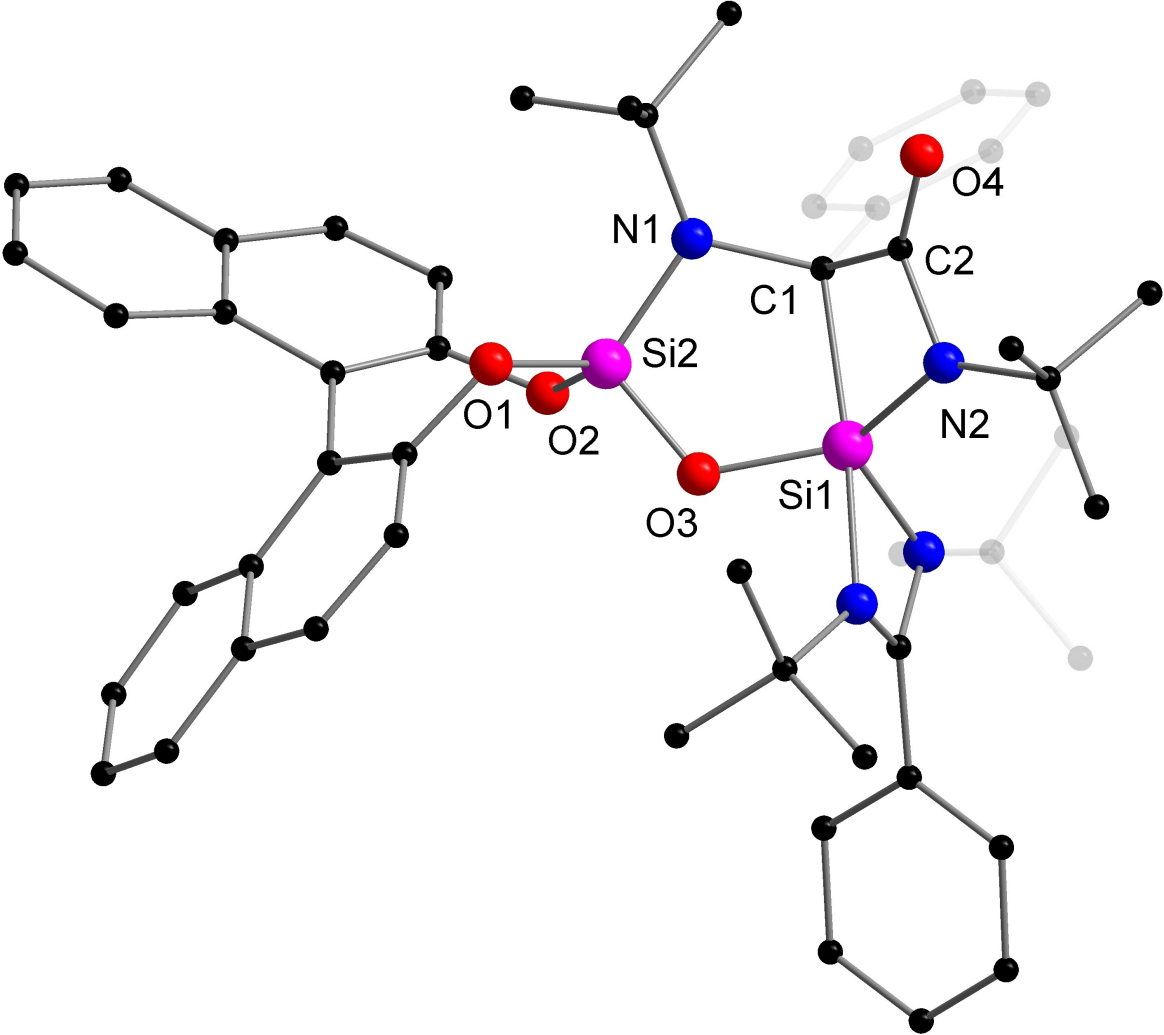
Molecular structure of compound **3** in the solid state, hydrogen atoms and non‐coordinating solvent molecules are omitted for clarity. Selected bond distances [Å] and angles [°]: Si1−C1 2.018(2), Si1−N2 1.788(2), Si1−O3 1.678(2), Si2−O1 1.644(2), Si2−O2 1.647(2), Si2−O3 1.608(2), Si2−N1 1.683(2), C1−C2 1.526(3), C1−N1 1.482(3), C2−O4 1.213(3), C2−N2 1.375(3); O1−Si2−O2 105.58(9), Si1−O3−Si2 119.85(10), Si2−N1−C1 110.76(15), N1−C1−C2 115.3(2), N1−C1−Si1 109.08(15), C1−C2−N2 103.1(2).

The reaction product **3** was structurally characterized by X‐ray diffraction analysis. It crystallizes in the non‐centrosymmetric monoclinic space group *P*2_1_ with a flack parameter showing the formation of just one enantiomer.^#^ The molecular structure depicted in Figure 3 shows that **3** consists of two fused rings (CNSi_2_O and C_2_NSi), which are twisted by 46.1°. During the reaction, one of the C=O bonds of CO_2_ was completely cleaved and the isolated oxygen atom O3 is now bridging the two silicon atoms Si1 and Si2. The remaining carbonyl (C=O) moiety is coordinated to C1 and N2, giving rise to a new amide functional group. The carbonyl C=O distance (1.213(3) Å) is well comparable with the usual carbonyl C=O bond distances in organic amides (1.22–1.23 Å).^[21]^ The Si1−C1 bond distance is elongated to 2.016(2) Å and longer than the typical Si−C single bond lengths of silanes (1.85–1.89 Å).^[21]^ The addition of the carbonyl function to the C1 atom derived a new stereogenic center at C1. The value of the flack parameter (x=−0.01(5)) indicated the correct assignment of the absolute configuration around the C1 atom ((*R*)‐configuration).^#^ We were able to show by NMR spectroscopy and XRD, that indeed only one of the two possible diastereomers ((*S*,*R*) or (*S*,*S*)) was formed. In the ^13^C{^1^H} NMR spectrum, the signal for the carbonyl carbon atom was observed at *δ* 179.4 ppm and the one for the chiral quaternary carbon nucleus at *δ* 78.2 ppm, the assignment of signals were based on ^13^C‐DEPT, ^1^H‐^13^C‐HMBC NMR experiments and DFT calculations. The ^29^Si{^1^H} NMR spectrum displays two sharp singlets at *δ* −59.9 and −97.7 ppm, assignable to the resonances for the SiC and SiO_2_ silicon nuclei, respectively. In the IR spectrum, the C=O stretching band appears at 1645 cm^−1^. The formation of **3** provided an elegant pathway to access an enantiopure heterocycle featuring a carbonylated quaternary stereocenter through reductive cleavage of CO_2_.

**Figure 4:**
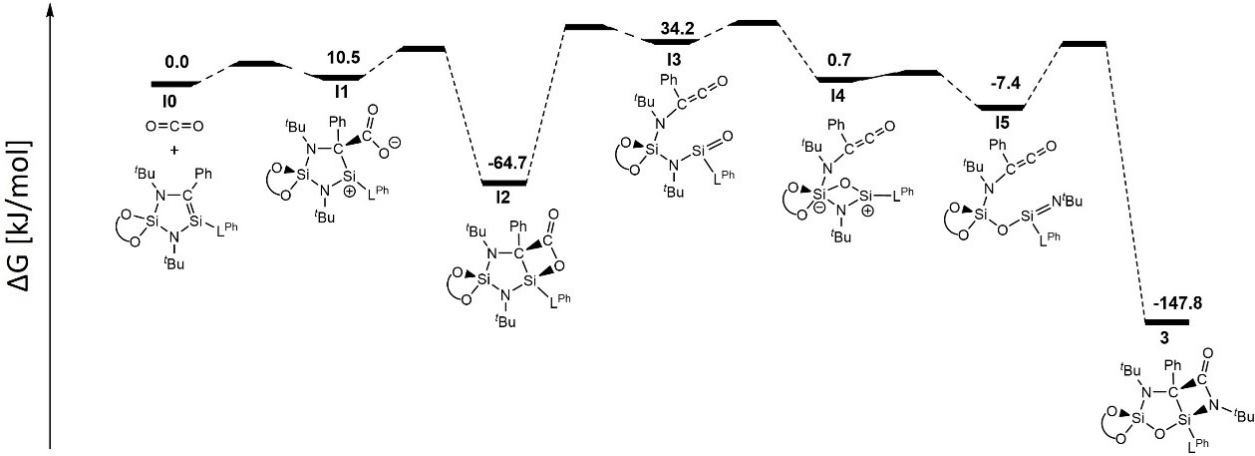
Calculated free energy diagram of the proposed mechanism of the formation of **3**.

Given the unusual structure of compound **3** and C=O double bond cleavage, DFT calculations were performed to shed light on the reaction mechanism. On a model compound with small substituents (**1^#^**, for details, see Figure S36), the whole sequence was calculated including activation barriers, while with the full molecule **1**, the transition states eluded localization and hence, only the intermediates are considered (Figure 4 and Figure S37). The likely reaction pathway proceeds via C−C bond formation between silene‐C and CO_2_ (**I1^#^**/**I1**). Then, the rotation of the CO_2_ moiety towards amidinate‐Si completes the [2+2] cycloaddition (**I2^#^**/**I2**). This bicylic species undergoes a cycloreversion reaction into a ketene and a silanone moiety (**I3^#^**/**I3**). The silanone‐O then attacks the BINOL−Si to give a Si_2_NO heterocycle (**I4^#^**/**I4**). This cycle opens up to the silaimine (**I5^#^**/**I5**), that then undergoes [2+2] cycloaddition with the ketene moiety again to give the final product **3^#^**/**3**. As no intermediates were observed spectroscopically, the reaction pathway solely rests on the plausibility of intermediates and activation barriers. With the model compound, the initial [2+2] addition product **I2^#^** is very stable and its cycloreversion reaction has a very high activation barrier, which would render this reaction impossible. However, when considering the whole molecule, **I2** is less stable due to steric strain resulting from the interaction of the N*t*Bu group with adjacent substituents. This steric strain can be relieved by the rearrangement reaction, that leaves the N*t*Bu group as an exocyclic moiety.

**Scheme 4:**
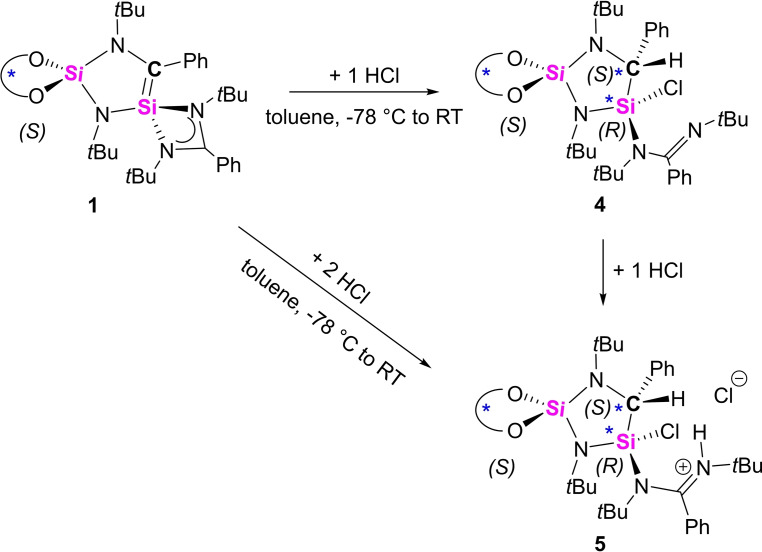
Stepwise reaction of **1** with HCl.

After adding a non‐polar molecule, we intended to further explore the reactivity of silene **1** with the polar HCl molecule. The addition of equimolar amount of HCl to silene **1** in toluene resulted in the completely regio‐ and stereoselective formation of the simple 1,2‐addition product **4** (Scheme 4). Analysis of the crude product by ^1^H NMR spectroscopy revealed only one set of the four *t*Bu signals (*δ* 1.53, 1.53, 1.16, 0.83 ppm), indicating only one diastereomer was formed. Thus, again the reaction proceeds with quantitative stereoselectivity. However, in this case even two new stereocenters were formed.

**Figure 5:**
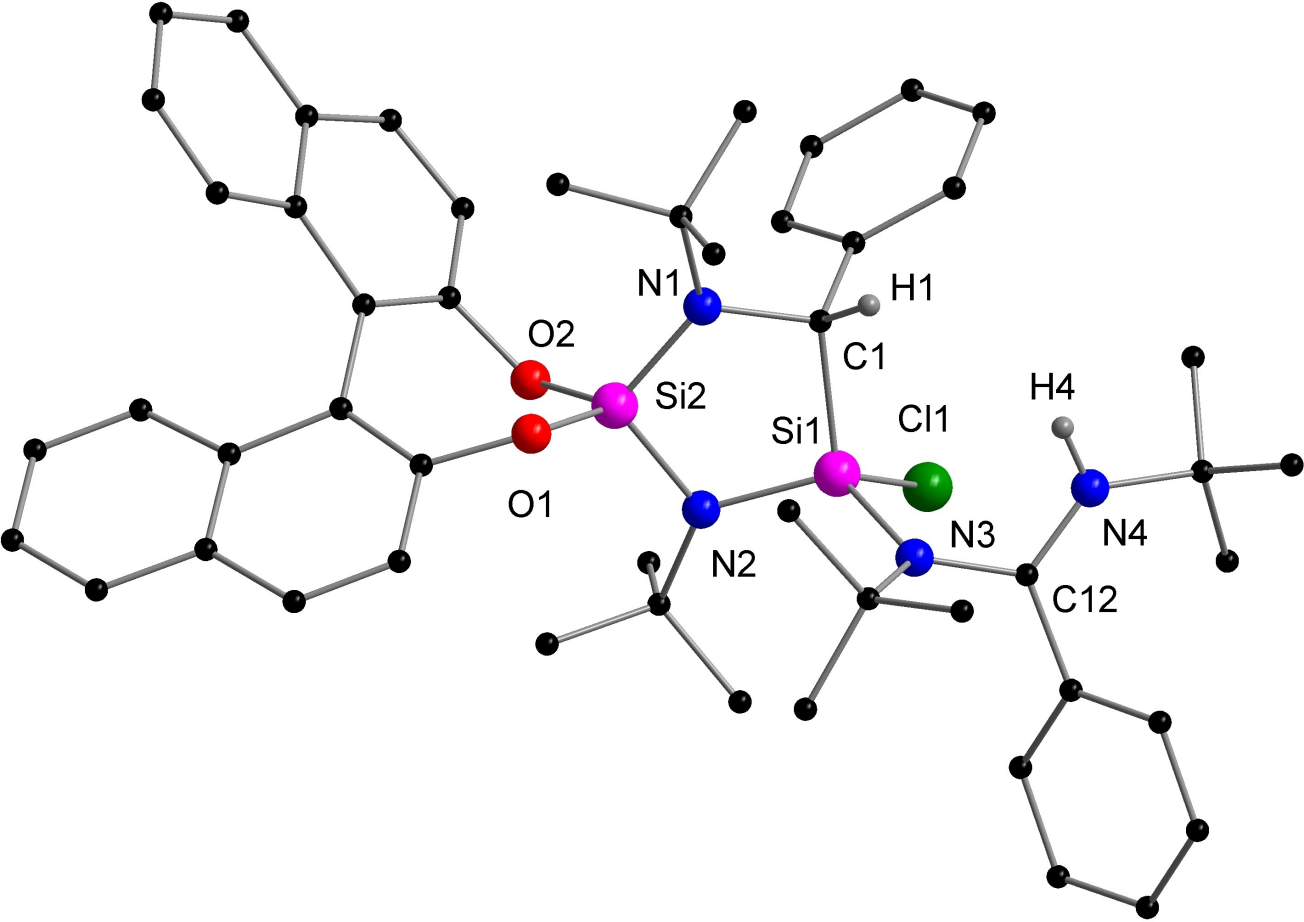
Molecular structure of the cation of compound **5** in the solid state, hydrogen atoms and non‐coordinating solvent molecules are omitted for clarity. Selected bond distances [Å] and angles [°]: Si1−Cl1 2.056(2), Si1−C1 1.900(6), Si1−N2 1.704(6), Si1−N3 1.770(6), Si2−N1 1.687(6), Si2−N2 1.718(6), Si2−O1 1.631(5), Si2−O2 1.641(5), N1−C1 1.479(8), N3−C12 1.372(9), N4−C12 1.283(9); C1−Si1−N2 100.5(3), C1−Si1−Cl1 109.2(2), C1−Si1−N3 117.3(3), Cl1−Si1−N2 110.5(2), Cl1−Si1−N3 102.5(2), N2−Si1−N3 116.9(3), Si2−N1−C1 114.5(4), N1−C1−Si1 106.1(4), Si1−N2‐Si2 109.7(3), O1−Si2−O2 103.9(3), N3−C12−N4 119.0(6).

The structure of the adduct was established by using a combination of 1D (^1^H, ^13^C, ^29^Si) and 2D NMR (^1^H‐^13^C‐HMQC/HMBC) spectroscopic methods as primary diagnostic tools. The ^1^H NMR signal at *δ* 4.50 ppm showed HMQC correlations to the ^13^C atom at 54.6 ppm. The latter signal could be assigned to the newly generated stereogenic carbon nucleus. In the ^29^Si{^1^H} NMR spectrum, two signals were detected at *δ* −21.8 and −49.7 ppm, slightly upfield shifted with respect to those of silene **1**. Using NMR spectroscopic methods, the regioselectivity of the reaction could be confirmed, but computations indicate that upon reaction with HCl, the amidinate acts only as monodentate substituent. For this species, excellent agreement of all NMR data with the predictions was found (Figure S36).^[22]^ However, NMR spectroscopy could not be used to distinguish between the different stereoisomers. Despite several attempts, no single crystals of **4** suitable for X‐ray diffraction analysis could be obtained. However, we found that the reaction between **1** and two equivalents of HCl in toluene resulted in the clean formation of the amidinium chloride salt **5** as colorless precipitate (Scheme 4). The ^1^H NMR spectrum shows the presence of four *t*Bu singlet signals (*δ* 1.65, 1.37, 1.36, 0.78 ppm) and features the two newly generated proton signals at *δ* 13.26 (NH) and 6.10 (CH) ppm. The molecular structure of the diastereoisomer was confirmed by X‐ray analysis on the single crystals of **5**. As shown in Figure 5, the five‐membered heterocycle is retained with the Si1−C1 bond length being 1.889(7) Å. The molecular structure reveals that one HCl molecule is added across the Si=C double bond, forming the saturated adduct with two stereogenic centers, C1 (*S*) and Si1 (*R*), while the amidinato functional group was protonated by the second equivalent of HCl molecule, forming the cationic amidinium moiety. The correct assignment of the absolute configurations around the stereogenic centers were supported by a low flack parameter and a non‐centrosymmetric space group (*P*2_1_).^#^ With **4** and **5** in hand, we expected that **5** could be formed directly from **4**. Indeed, treatment of **4** with 1 molar equiv. of HCl in toluene at ambient temperature furnished **5**. This gave us more detailed information of the molecular structure of the diastereoisomer **4**.

## Conclusion

In summary, a facile method of generating the first example of a chiral cyclic silene species **1** comprising an (*S*)‐BINOL ligand scaffold under mild reaction conditions was provided. The unique bonding feature and ring structure in **1** was used to demonstrate further reactivity with elemental sulfur, CO_2_ and HCl. The reaction with S_8_ led to the ring‐opening product **2** with imine and silanethione functional groups. Moreover, **1** is capable of cleaving the CO_2_ molecule into a carbonyl group and an isolated O atom, thus a new stereocenter was formed in a highly selective manner. The stepwise reaction with HCl molecule to the saturated silane **4** and the cationic compound **5** further shows the high reactivity of the double bond in **1**. In this case even two new stereocenters are formed fully selectively. The chiral (*S*)‐BINOL scaffold in the silene **1** enabled unprecedented complete control over the diastereoselectivity in the reactions with the respective small molecules.

Deposition Numbers 2164389, 2164390, 2164391, 2164392 contain the supplementary crystallographic data for this paper. These data are provided free of charge by the joint Cambridge Crystallographic Data Centre and Fachinformationszentrum Karlsruhe Access Structures service.

# Single crystal X‐ray diffraction is not a method of bulk analysis. In principle, both enantiomers could be formed. However, presuming the configurational stability of the BINOL backbone, only (*S*)‐BINOL should be present.

## Conflict of interest

The authors declare no conflict of interest.

1

## Supporting information

As a service to our authors and readers, this journal provides supporting information supplied by the authors. Such materials are peer reviewed and may be re‐organized for online delivery, but are not copy‐edited or typeset. Technical support issues arising from supporting information (other than missing files) should be addressed to the authors.

Supporting Information

## Data Availability

The data that support the findings of this study are available in the supplementary material of this article.
