# Supplementary material for: Stereoselective Activation of Small Molecules by a Stable Chiral Silene
Source: Chemistry. 2022 Aug 3;28(55):e202201963. doi: 10.1002/chem.202201963 (PMC9805157; doi:10.1002/chem.202201963)
Supplement: Supplementary file 1 — Supporting Information [file CHEM-28-0-s001.pdf]

Chemistry–A European Journal

Supporting Information

Stereoselective Activation of Small Molecules by a Stable Chiral Silene

Xiaofei Sun, Alexander Hinz, Hannes Kucher, Michael T. Gamer, and Peter W. Roesky*

Table of Contents

I. Synthesis and characterization S3

I.1 General procedures S3

I.2 Synthesis of **1** S4

1.3 Synthesis of **2** S5

1.3 Synthesis of **3** S6

1.4 Synthesis of **4** S7

1.5 Synthesis of **5** S8

II. NMR spectra S10

IV. IR spectra S23

V. X-ray crystallography S26

V.1 General methods S26

V.2. Summary of crystal data S27

V.3 Crystal structures S28

VI. Quantum chemical calculations S32

VI.1. Silene S32

VI.2. S reaction S33

VI.3. CO_2 reaction S34

VI.4. HCl reaction S36

VI.5. Optimized Structures S37

VI.5.1. Compound **1** S37

VI.5.2. Compound **2’** S38

VI.5.3. Compound **2** S40

VI.5.4. Compound **3** S42
VI.5.5. Compound **4** N off S43
VI.5.6. Compound **4** N on S45
VI.5.7. S_**Int 1 (2A)** S47
VI.5.8. S_**Int 1 (2B)** S49
VI.5.9. S_**Int 2 (2C-1)** S50
VI.5.10. S_ **Int 2 (2C-2)** S52
VI.5.11. S_ **Int 2 (2C-3)** S54
VI.5.12. CO_2_**Int1** S56
VI.5.13. CO_2_**Int2** S57
VI.5.14. CO_2_**Int3** S59
VI.5.15. CO_2_**Int4** S61
VI.5.16. CO_2_**Int5** S63
VI.5.17. Compound **5** S64
VI.5.18. CO_2_$\mathbf{1^M}$ S66
VI.5.19. CO_2_$\mathbf{TS_1^M}$ S67
VI.5.20. CO_2_$\mathbf{I_1^M}$ S67
VI.5.21. CO_2_$\mathbf{TS_2^M}$ S68
VI.5.22. CO_2_$\mathbf{I_2^M}$ S69
VI.5.23. CO_2_$\mathbf{TS_3^M}$ S70
VI.5.24. CO_2_$\mathbf{I_3^M}$ S70
VI.5.25. CO_2_$\mathbf{TS_4^M}$ S71
VI.5.26. CO_2_$\mathbf{I_4^M}$ S72
VI.5.27. CO_2_$\mathbf{TS_5^M}$ S72
VI.5.28. CO_2_$\mathbf{I_5^M}$ S73
VI.5.29. CO_2_$\mathbf{TS_6^M}$ S74
VI.5.30. CO_2_$\mathbf{3^M}$ S74
VII. References S76

I. Synthesis and characterization

I.1 General procedures

All air- and moisture-sensitive manipulations were performed under dry N_2 or Ar atmosphere using standard Schlenk techniques or in an argon-filled MBraun glovebox, unless otherwise stated. Et_2O, *n*-pentane, and toluene were dried using an MBraun solvent purification system (SPS-800) and degassed. Benzene and THF were distilled under nitrogen from potassium benzophenone ketyl. C_6D_6, toluene-d_8 and THF-d_8 were dried over Na-K alloy and degassed by freeze-pump-thaw cycles. L^{Ph}SiCl was prepared according to the literature procedures.[1] All other chemicals were obtained from commercial sources and used without further purification.

Elemental analyses were carried out with an Elementar vario MICRO cube.

NMR spectra were recorded on Bruker spectrometers (Avance III 300 MHz, Avance 400 MHz or Avance III 400 MHz). Chemical shifts are referenced internally using signals of the residual protio solvent (^{1}H) or the solvent (^{13}C{^{1}H}) and are reported relative to tetramethylsilane (^{1}H, ^{13}C{^{1}H}), or externally relative to tetramethylsilane (^{29}Si), H_3PO_4 (^{31}P). All NMR spectra were measured at 298 K, unless otherwise specified. The multiplicity of the signals is indicated as s = singlet, d = doublet, dd = doublet of doublets, t = triplet, q = quartet, m = multiplet and br = broad. Assignments were determined based on unambiguous chemical shifts, coupling patterns and ^{13}C-DEPT experiments or 2D correlations (^{1}H-^{1}H COSY, ^{1}H-^{13}C HMQC and ^{1}H-^{13}C HMBC).

Infrared (IR) spectra were recorded in the region 4000–400 cm^{-1} on a Bruker Tensor 37 FTIR spectrometer equipped with a room temperature DLaTGS detector, a diamond attenuated total reflection (ATR) unit and a nitrogen-flushed chamber. In terms of their intensity, the signals were classified into different categories (vs = very strong, s = strong, m = medium, w = weak, and sh = shoulder).

I.2 Synthesis of 1

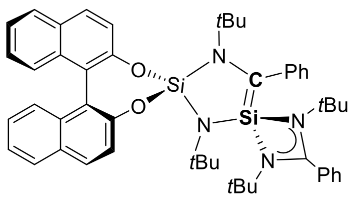

1

To a solution of 1.96 g (*S*)-BINOL (6.85 mmol, 1.00 eq) in 40 mL of THF was added dropwise 5.5 mL of a solution of *n*-BuLi (2.5 M in hexanes, 13.75 mmol, 2.01 eq.) at –88 °C. After warming to room temperature, the reaction mixture was stirred for 16 h, which resulted in a pale-yellow solution. The solution was cooled to –88 °C and a solution of 4.01 g $L^{Ph}SiCl$ (13.60 mmol, 2.00 eq) in 40 mL toluene was added dropwise. The reaction mixture was stirred for 1.5 h at –88 °C before it was allowed to warm up to room temperature. After stirring for 16 h at room temperature, the reaction mixture became dark brown. All volatiles were removed under reduced pressure and the residue was extracted with 150 mL of toluene. The solution was concentrated and kept at -30 °C for crystallization. Crystalline yield: 3.80 g (69%).

Orange needles suitable for X-ray diffraction analysis could be obtained at room temperature from toluene.

Anal. Calcd for $C_{50}H_{58}N_4O_2Si_2 \cdot 0.6\,(C_7H_8)$ (858.49): C 75.83; H 7.37; N 6.53. Found: C 75.95, H 7.27; N 6.57.

^{1}H NMR (400.30 MHz, C_6D_6): δ (ppm) = 8.02 (d, $^3J_{HH}$ = 8.6 Hz, 1H, $CH_{Ar,\mathbf{1}}$), 7.81-7.68 (m, 6H, $CH_{Ar,\mathbf{1}}$), 7.54 (ddd, $^3J_{HH}$ = 8.3 Hz, $^3J_{HH}$ = 7.0 Hz, $^4J_{HH}$ = 1.6 Hz, 1H, $CH_{Ar,\mathbf{1}}$), 7.45-7.37 (m, 4H, $CH_{Ar,\mathbf{1}}$), 7.16-7.10 (m, 6H, $CH_{Ar,\mathbf{1}}$), 7.06-7.01 (m, 1H, $CH_{Ar,\mathbf{1}}$), 6.97-6.84 (m, 8H, $CH_{Ar,\mathbf{1}}$), 2.11 (s, 1.8H, $CH_{3\,tol}$), 1.52 (s, 9H, $C(CH_3)_3$), 1.36 (s, 9H, $C(CH_3)_3$), 1.20 (s, 9H, $C(CH_3)_3$), 1.11 (s, 9H, $C(CH_3)_3$).

^{13}C{^{1}H} NMR (75.47 MHz, C_6D_6): δ (ppm) = 177.4 (N*C*N), 155.2 ($C_{q,\mathbf{1}}$), 152.8 ($C_{q,\mathbf{1}}$-OSi), 152.1 ($C_{q,\mathbf{1}}$-OSi), 137.9 ($C_{q,tol}$), 134.8 ($C_{q,\mathbf{1}}$), 134.4 ($C_{q,\mathbf{1}}$), 131.2 ($C_{q,\mathbf{1}}$), 130.9 ($C_{q,\mathbf{1}}$), 130.8 ($C_{Ar,\mathbf{1}}$), 130.3 ($C_{q,\mathbf{1}}$), 129.6 ($C_{Ar,\mathbf{1}}$), 129.6 ($C_{Ar,\mathbf{1}}$), 129.3 ($C_{Ar,tol}$), 128.6 ($C_{Ar,tol}$), 128.6 ($C_{Ar,\mathbf{1}}$), 128.4 ($C_{Ar,\mathbf{1}}$), 128.3 ($C_{Ar,\mathbf{1}}$), 128.2 ($2C_{Ar,\mathbf{1}}$), 127.9 ($C_{Ar,\mathbf{1}}$), 127.6 ($C_{Ar,\mathbf{1}}$), 127.4 ($C_{Ar,\mathbf{1}}$), 127.4 ($C_{Ar,\mathbf{1}}$), 127.0 ($C_{Ar,\mathbf{1}}$), 126.4 ($C_{Ar,\mathbf{1}}$), 126.2 ($C_{Ar,\mathbf{1}}$), 126.1 ($C_{Ar,\mathbf{1}}$), 125.7 ($C_{Ar,tol}$), 124.2 ($C_{Ar,\mathbf{1}}$), 124.1 ($C_{Ar,\mathbf{1}}$), 123.6 ($C_{Ar,\mathbf{1}}$), 123.3 ($C_{Ar,\mathbf{1}}$), 121.3 ($C_{q,\mathbf{1}}$), 121.2 ($C_{q,\mathbf{1}}$), 119.5 ($C_{Ar,\mathbf{1}}$), 114.5 ($C_{Ar,\mathbf{1}}$), 58.2 (Si=*C*), 57.6 ($C(CH_3)_3$), 55.7 ($C(CH_3)_3$), 55.1 ($C(CH_3)_3$), 52.7 ($C(CH_3)_3$), 32.8 ($C(CH_3)_3$), 31.8 ($C(CH_3)_3$), 31.1 ($C(CH_3)_3$), 30.7 ($C(CH_3)_3$), 21.5 ($CH_{3,tol}$).

^{29}Si{^{1}H} NMR (79.52 MHz, C_6D_6): δ (ppm) = -27.5 (*Si*=C), -61.3 (SiO_2).

IR (ATR): $\tilde{\nu}$ [cm^{-1}] = 3060 (w), 3035 (w), 2970 (s), 2931 (w), 2905 (w), 2868 (w), 2164 (w), 1698 (w), 1617 (w), 1586 (m), 1504 (w), 1468 (m), 1426 (w), 1394 (s), 1364 (m), 1330 (m), 1291 (w), 1273 (m), 1242 (m), 1220 (w), 1199 (s), 1170 (w), 1143 (w), 1093 (w), 1067 (s), 1034 (w), 1002 (m), 990 (w), 956 (s), 921 (w), 873 (s), 849 (w), 817 (m), 791 (w), 769 (w), 748 (s), 730 (m), 698 (m), 685 (w),

659 (m), 644 (w), 592, (w), 577 (w), 566 (w), 542 (w), 515 (w), 498 (w), 483 (w), 464 (w), 435 (w), 418 (w), 372 (w).

1.3 Synthesis of 2

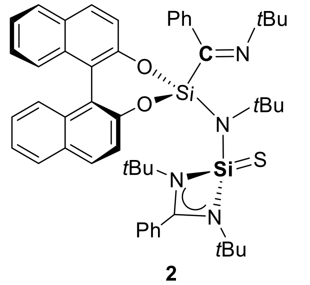

2

Toluene (10 mL) was added to a mixture of 214 mg (0.249 mmol, 1.00 eq) of compound **1** and 9 mg (0.033 mmol, 0.140 eq) of S_8 at room temperature. The reaction mixture turned from dark brown to light yellow within one minute. Then the mixture was heated up to 60 °C and stirred at 60 °C for 48 h, the mixture was then filtered over celite, and all solvents were removed under reduced pressure. The remaining sticky solid was washed with *n*-pentane (10 mL) and dried to yield the crude product as white powder. The crude product was redissolved in toluene (6 mL) and the solution was concentrated to ca 3 mL for crystallization. Crystalline yield: 79 mg (38%).

Colorless crystals suitable for X-ray diffraction analysis could be obtained at room temperature from toluene.

Anal. Calcd for $C_{50}H_{58}N_4O_2Si_2{\cdot}0.3\ (C_7H_8)$ (862.91): C 72.52; H 7.06; N 6.49, S 3.72. Found: C 72.68, H 6.78; N 6.39, S 3.36.

^{1}H NMR (400.30 MHz, C_6D_6): *δ* (ppm) = 7.81-6.55 (m, *ca.* 40H, CH_{Ar}), 6.04 (br s, 1H, CH_{Ar}), 5.11 (br s, 1H, CH_{Ar}), 2.39 (s, 9H, C(CH_3)$_3$ 1), 2.11 (s, ca. 1.6H, C$H_{3\,tol}$), 1.75 (s, 9H, C(CH_3)$_3$ 1), 1.65 (s, 6.7H, C(CH_3)$_3$ 2), 1.41 (s, 6.7H, C(CH_3)$_3$ 2), 1.35 (s, 6.7H, C(CH_3)$_3$ 2), 1.35 (s, 6.7H, C(CH_3)$_3$ 2), 1.19 (s, 9H, C(CH_3)$_3$ 1), 0.56 (s, 9H, C(CH_3)$_3$ 1).

^{1}H NMR (400.30 MHz, $CDCl_3$): *δ* (ppm) = 8.06-6.51 (m, *ca.* 34H, CH_{Ar}), 5.95 (br s, 1H, CH_{Ar}), 4.87 (br s, 1H, CH_{Ar}), 2.36 (s, ca. 0.8H, C$H_{3\,tol}$), 2.08 (s, 9H, C(CH_3)$_3$ 1), 1.59 (s, 9H, C(CH_3)$_3$ 1), 1.50 (s, 4H, C(CH_3)$_3$ 2), 1.43 (s, 4H, C(CH_3)$_3$ 2), 1.40 (s, 4H, C(CH_3)$_3$ 2), 1.12 (s, 4H, C(CH_3)$_3$ 2), 1.11 (s, 9H, C(CH_3)$_3$ 1), 0.32 (s, 9H, C(CH_3)$_3$ 1).

^{13}C{^{1}H} NMR (100.67 MHz, C_6D_6): *δ* (ppm) = 183.2 (N=*C*), 181.5 (N=*C*), 174.8 (N*C*N), 174.6 (N*C*N), 154.5-120.3 (*C*$_{Ar}$), 61.9 (*C*(CH$_3$)$_3$ 1), 59.6 (*C*(CH$_3$)$_3$ 2), 58.2 (*C*(CH$_3$)$_3$ 1), 56.8 (*C*(CH$_3$)$_3$ 2), 55.6 (*C*(CH$_3$)$_3$ 2), 55.5 (*C*(CH$_3$)$_3$ 2), 55.4 (*C*(CH$_3$)$_3$ 1), 54.4 (*C*(CH$_3$)$_3$ 1), 34.9 (C(*C*H$_3$)$_3$ 1), 32.3 (C(*C*H$_3$)$_3$ 1), 32.3 (C(*C*H$_3$)$_3$ 2), 32.0 (C(*C*H$_3$)$_3$ 2), 31.8 (C(*C*H$_3$)$_3$ 1), 31.8 (C(*C*H$_3$)$_3$ 2), 31.7 (C(*C*H$_3$)$_3$ 2), 30.3 (C(*C*H$_3$)$_3$ 1), 21.4 (*C*H$_{3,tol}$).

$^{13}C\{^{1}H\}$ **NMR** (100.67 MHz, $CDCl_3$): *δ* (ppm) = 182.0 (N=*C*), 180.7 (N=*C*), 175.1 (N*C*N), 172.8 (N*C*N), 154.4-120.0 (C_{Ar}), 61.9 (*C*(CH_3)$_3$ 1), 59.4 (*C*(CH_3)$_3$ 2), 58.0 (*C*(CH_3)$_3$ 1), 56.9 (*C*(CH_3)$_3$ 2), 55.8 (*C*(CH_3)$_3$ 2), 55.7 (*C*(CH_3)$_3$ 2), 55.3 (*C*(CH_3)$_3$ 1), 54.3 (*C*(CH_3)$_3$ 1), 34.6 (C(*C*H_3)$_3$ 1), 32.1 (C(*C*H_3)$_3$ 2), 32.0 (C(*C*H_3)$_3$ 1), 31.9 (C(*C*H_3)$_3$ 2), 31.8 (C(*C*H_3)$_3$ 2), 31.7 (C(*C*H_3)$_3$ 2), 31.7 (C(*C*H_3)$_3$ 1), 30.1 (C(*C*H_3)$_3$ 1), 21.6 ($CH_{3,tol}$).

$^{29}Si\{^{1}H\}$ **NMR** (79.52 MHz, C_6D_6): *δ* (ppm) = –13.7, –20.0, –55.8, –64.5.

$^{29}Si\{^{1}H\}$ **NMR** (79.52 MHz, $CDCl_3$): *δ* (ppm) = –13.1, –20.0, –56.5, –64.9.

IR (ATR): $\tilde{\nu}$ [cm^{-1}] = 3084 (w), 3060 (w), 3033 (w), 2965 (vs), 2905 (sh), 2872 (sh), 1640 (sh), 1616 (s), 1591 (s), 1504 (m), 1464 (s), 1410 (vs), 1360 (s), 1332 (s), 1273 (w), 1245 (s), 1233 (s), 1220 (s), 1201 (sh), 1175 (sh), 1143 (sh), 1110 (sh), 1093 (m), 1072 (m), 1045 (sh), 999 (w), 956 (s), 936 (vs), 895 (s), 865 (w), 846 (m), 831 (m), 816 (s), 789 (w), 773 (w), 750 (s), 708 (s), 678 (m), 659 (w), 631 (w), 586 (w), 574 (s), 554 (m), 523 (m) 501 (m), 488 (w), 435 (m), 418 (w).

1.3 Synthesis of 3

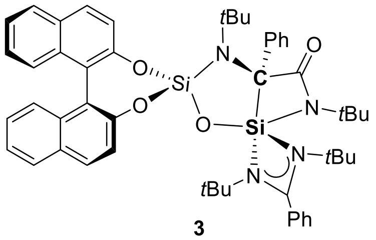

3

In a 250 mL Young Schlenk flask, 0.150 g of compound **1** (0.175 mmol) was dissolved in 15 mL of toluene and the solution was degassed by freeze-pump-thaw for three times. The Young Schlenk flask was then filled with CO_2 at room temperature and stirred for 48 h. During the reaction time, the color of the solution turned from dark brown to colorless. All volatiles were removed under reduced pressure and the remaining residue was extracted with *n*-pentane (200 mL) and dried. The crude product was recrystallized from benzene.

Crystals suitable for X-ray diffraction analysis were grown from a highly concentrated solution in benzene. Crystalline yield: 0.051 g (34%).

Anal. Calcd for $C_{51}H_{58}N_4O_4Si_2$·(0.5 C_6H_6) (925.33): C 73.18; H 6.94; N 6.32. Found: C 73.37, H 6.52; N 5.81.

(The presence of C_6H_6 in the lattice of the crystals was confirmed by XRD and NMR analysis)

^{1}H **NMR** (400.30 MHz, C_6D_6): *δ* (ppm) = 8.67 (d, $^{3}J_{HH}$ = 7.9 Hz, 1H, CH_{Ar}), 7.89 (d, $^{3}J_{HH}$ = 8.8 Hz, 1H, CH_{Ar}), 7.83 (d, $^{3}J_{HH}$ = 7.8 Hz, 1H, CH_{Ar}), 7.74-7.60 (m, 7H, CH_{Ar}), 7.41 (d, $^{3}J_{HH}$ = 8.6 Hz, 1H, CH_{Ar}), 7.28 (d, $^{3}J_{HH}$ = 8.4 Hz, 1H, CH_{Ar}), 7.24 (t, $^{3}J_{HH}$ = 7.6 Hz, 1H, CH_{Ar}), 7.15-7.03 (m, 5H, CH_{Ar}), 6.89 (t, $^{3}J_{HH}$ =

7.7 Hz, 1H, CH_{Ar}), 6.82-6.77 (m, 2H, CH_{Ar}), 6.64 (br s, 4H, CH_{Ar}), 1.82 (s, 9H, C(CH_3)$_3$), 1.41 (s, 9H, C(CH_3)$_3$), 1.11 (s, 9H, C(CH_3)$_3$), 0.60 (s, 9H, C(CH_3)$_3$).

^{13}C{^{1}H} NMR (100.67 MHz, C_6D_6): δ (ppm) = 179.4 (*C*=O), 173.2 (N*C*N), 151.8 (C_q), 151.8 (C_q), 149.4 (C_q), 134.3 (C_q), 134.2 (C_q), 132.9 (C_q), 130.9 (C_q), 130.6 (C_q), 130.6 (C_{Ar}), 129.9 (2C_{Ar}), 129.7 (C_{Ar}), 128.9 (C_{Ar}), 128.6 ($C_{Ar,C6H6}$), 128.5 (C_{Ar}), 128.4 (C_{Ar}), 128.2 (C_{Ar}), 128.1 (2C_{Ar}), 128.0 (C_{Ar}), 127.9 (2C_{Ar}), 127.6 (C_{Ar}), 127.4 (C_{Ar}), 126.4 (C_{Ar}), 126.3 (C_{Ar}), 124.9 (C_{Ar}), 124.5 (C_{Ar}), 124.3 (C_{Ar}), 123.7 (C_{Ar}), 122.9 (C_{Ar}), 121.5 (C_q), 120.9 (C_q), 78.2 (Si-C), 56.0 (C(CH$_3$)$_3$), 54.9 (*C*(CH$_3$)$_3$), 54.7 (*C*(CH$_3$)$_3$), 54.2 (*C*(CH$_3$)$_3$), 32.7 (C(*C*H$_3$)$_3$), 32.3 (C(*C*H$_3$)$_3$), 31.4 (C(*C*H$_3$)$_3$), 29.6 (C(*C*H$_3$)$_3$).

^{29}Si{^{1}H} NMR (79.52 MHz, C_6D_6): δ (ppm) = –59.9 (*Si*O$_2$), –97.7 (*Si*C).

IR (ATR): $\tilde{\nu}$ [cm^{-1}] = 3057 (w), 2964 (s), 2928 (w), 2869 (w), 2324 (w), 2164 (w), 1995 (w), 1645 (s), 1621 (w), 1621 (m), 1594 (s), 1506 (m), 1539 (w), 1467 (s), 1446 (m), 1429 (m), 1393 (s), 1360 (s), 1332 (s), 1271 (w), 1245 (vs), 1221 (m), 1201 (s), 1155 (w), 1109 (m), 1070 (m), 1042 (s), 996 (vs), 945 (w), 862 (s), 844 (w), 814 (s), 788 (w), 772 (w), 746 (s), 699 (s), 730 (w), 697 (s), 656 (w), 634 (m), 606 (w), 582 (w), 563 (m), 520 (w), 490 (w), 468 (w), 416 (w).

1.4 Synthesis of 4

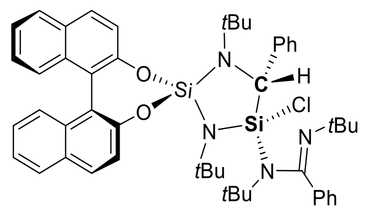

4

In a Schlenk flask, 0.310 g of compound **1** (0.361 mmol) was dissolved in 15 mL of toluene and the solution was cooled down to –88 °C. 3.6 mL of HCl (0.1 M in toluene/1,4-dioxane) was added slowly dropwise at –88 °C. The solution was stirred at this temperature for 30 min before it was allowed to warm up to room temperature. After stirring for *ca.* 30 min at room temperature, the solution turned colorless. After stirring for 16 h at room temperature, all volatiles were removed under reduced pressure and the residue was washed with *ca*. 5 mL of cold *n*-pentane before the residue was extracted with 50 mL of *n*-pentane to obtain the final product **4** as analytically pure colorless powder. Yield: 0.130 g (43%).

Anal. Calcd for $C_{50}H_{59}ClN_4O_2Si_2$ (839.67): C 71.52; H 7.08; N 6.67. Found: C 70.93, H 6.22; N 5.60.

^{1}H NMR (400.30 MHz, C_6D_6): δ (ppm) = 7.80 (d, $^3J_{HH}$ = 8.8 Hz, 1H, CH_{Ar}), 7.74-7.67 (m, 4H, CH_{Ar}), 7.57 (t, $^3J_{HH}$ = 8.3 Hz, 2H, CH_{Ar}), 7.50-7.31 (m, 6H, CH_{Ar}), 7.32 (d, $^3J_{HH}$ = 8.5 Hz, 1H, CH_{Ar}), 7.23 (t, $^3J_{HH}$ = 7.3 Hz, 1H, CH_{Ar}), 7.14-7.04 (m, 7H, CH_{Ar}), 6.87 (q, $^3J_{HH}$ = 8.2 Hz, 2H, CH_{Ar}), 4.50 (s, 1H, Si-C*H*), 1.53 (s, 18H, C(CH_3)$_3$), 1.16 (s, 9H, C(CH_3)$_3$), 0.83 (s, 9H, C(CH_3)$_3$).

^{13}C{^{1}H} NMR (100.67 MHz, C_6D_6): δ (ppm) = 152.2 (C_q), 152.0 (C_q), 147.9 (C_q), 142.3 (C_q), 134.6 (C_q), 134.5 (C_q), 130.9 (C_q), 130.8 (2C_{Ar}), 130.4 (C_q), 129.9 (C_{Ar}), 129.4 (2C_{Ar}), 128.7 (C_{Ar}), 128.5 (C_{Ar}), 128.3 (C_{Ar}), 128.3 (C_{Ar}), 128.0 (C_{Ar}), 127.8 (C_{Ar}), 127.7 (2C_{Ar}), 127.6 (C_{Ar}), 126.4 (C_{Ar}), 126.4 (C_{Ar}), 126.2 (C_{Ar}), 124.5 (C_{Ar}), 124.4 (C_{Ar}), 123.8 (C_{Ar}), 123.3 (C_{Ar}), 121.8 (C_q), 120.2 (C_q), 56.1 ($C(CH_3)_3$), 56.0 ($C(CH_3)_3$), 56.0 ($C(CH_3)_3$), 55.4 ($C(CH_3)_3$), 54.6 (Si-CH), 33.7 ($C(CH_3)_3$), 33.4 ($C(CH_3)_3$), 32.3 ($C(CH_3)_3$), 31.4 ($C(CH_3)_3$).

The signals of the N*C*N and one C_{Ar} could not be detected.

^{29}Si{^{1}H} NMR (79.52 MHz, C_6D_6): δ (ppm) = –21.8 (*Si*-C), –49.7 (*Si*O_2).

IR (ATR): $\tilde{\nu}$ [cm^{-1}] = 3085 (w), 3061 (w), 2970 (s), 2909 (sh), 1615 (s), 1594 (m), 1505 (m), 1466 (s), 1427 (m), 1390 (sh), 1366 (s), 1329 (s), 1272 (w), 1243 (vs), 1225 (sh), 1186 (s), 1102 (w), 1072 (m), 1051 (sh), 1005 (m), 964 (s), 931 (m), 914 (s), 847 (m), 815 (m), 772 (m), 748 (m), 730 (w), 706 (m), 679 (w), 657 (w), 597 (w), 566 (w), 545 (w), 504 (w), 464 (w), 417 (w).

1.5 Synthesis of 5

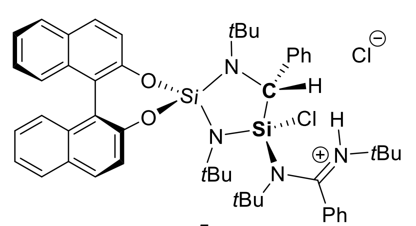

5

In a Schlenk flask, 0.140 g of compound **1** (0.163 mmol) was dissolved in 15 mL of toluene and the solution was cooled down to –88 °C. 3.3 mL of HCl (0.1 M in toluene/1,4-dioxane) was added slowly dropwise at –88 °C. The solution was warmed up to room temperature and turned colorless. Colorless precipitate formed immediately. After stirring for 16 h at room temperature, the colorless precipitate was collected by filtration and dried for ca. 2 h *in vacuo* obtain the final product **5** as analytically pure colorless powder. Yield: 0.95 g (67%).

Colorless crystals suitable for X-ray diffraction analysis could be obtained at room temperature from a mixture of toluene/dichloromethane.

Anal. Calcd for $C_{50}H_{60}N_4O_4Si_2Cl_2 \cdot 0.7\ (C_7H_8)$ (940.62): C 70.10; H 7.03; N 5.96. Found: C 70.53, H 6.60; N 6.27.

(The presence of C_7H_8 in the lattice of the crystals was confirmed by XRD and NMR analysis)

^{1}H NMR (400.30 MHz, CD_2Cl_2): δ (ppm) = 13.26 (N*H*), 8.26 (d, $^{3}J_{HH}$ = 5.6 Hz, 1H, CH_{Ar}), 8.00 (d, $^{3}J_{HH}$ = 8.0 Hz, 1H, CH_{Ar}), 8.00 (d, $^{3}J_{HH}$ = 8.9 Hz, 1H, CH_{Ar}), 7.88 (t, $^{3}J_{HH}$ = 9.1 Hz, 3H, CH_{Ar}), 7.77-7.66 (m,

4H, CH_{Ar}), 7.60-7.50 (m, 4H, CH_{Ar}), 7.37-7.33 (m, 2H, CH_{Ar}), 7.30 (d, $^3J_{HH}$ = 8.9 Hz, 1H, CH_{Ar}), 7.26-7.22 (m, 2.5H, CH_{Ar}), 7.18-7.10 (m, 6H, CH_{Ar}), 7.02 (d, $^3J_{HH}$ = 8.9 Hz, 1H, CH_{Ar}), 6.10 (s, 1H, Si-C*H*), 2.34 (s, 2.1H, C$H_{3\,tol}$), 1.65 (s, 9H, C(CH_3)$_3$), 1.37 (s, 9H, C(CH_3)$_3$), 1.36 (s, 9H, C(CH_3)$_3$), 0.78 (s, 9H, C(CH_3)$_3$).

^{13}C{^{1}H} NMR (100.67 MHz, CD_2Cl_2): δ (ppm) = 176.0 (N*C*N), 151.9 (C_q), 151.6 (C_q), 148.2 (C_q), 138.4 ($C_{q,tol}$), 134.4 (C_q), 134.2 (C_q), 134.2 (C_q), 134.0 (C_{Ar}), 131.8 (C_{Ar}), 131.2 (C_{Ar}), 131.2 (C_{Ar}), 130.8 (C_q), 130.8 (C_{Ar}), 130.5 (C_{Ar}), 130.4 (C_q), 130.3 (C_{Ar}), 129.4 (C_{tol}), 129.2 (C_{Ar}), 128.7 (C_{Ar}), 128.6 (C_{tol}), 128.5 (C_{Ar}), 128.4 (C_{Ar}), 128.2 (C_{Ar}), 128.1 (C_{Ar}), 127.6 (C_{Ar}), 127.3 (C_{Ar}), 126.3 (C_{Ar}), 126.2 (C_{Ar}), 126.0 (C_{Ar}), 125.6 (C_{tol}), 124.6 (C_{Ar}), 124.5 (C_{Ar}), 123.3 (C_{Ar}), 122.9 (C_q), 121.2 (C_q), 119.9 (C_{Ar}), 62.6 (C(CH_3)$_3$), 62.3 (C(CH_3)$_3$), 57.0 (*C*(CH_3)$_3$), 55.6 (*C*(CH_3)$_3$), 52.0 (Si-*C*H), 33.7 (C(*C*H_3)$_3$), 33.2 (C(*C*H_3)$_3$), 31.3 (C(*C*H_3)$_3$), 30.1 (C(*C*H_3)$_3$), 21.5 (*C*$H_{3\,tol}$).

^{29}Si{^{1}H} NMR (79.52 MHz, CD_2Cl_2): δ (ppm) = –18.0 (*Si*-C), –49.1 (*Si*O_2).

IR (ATR): $\tilde{\nu}$ [cm^{-1}] = 2980 (m), 2912 (w), 2888 (w), 2523 (w), 1699 (w), 1617 (w), 1596 (m), 1570 (m), 1505 (m), 1479 (w), 1462 (s), 1424 (m), 1396 (m), 1369 (s), 1325 (m), 1306 (m), 1269 (w), 1242 (vs), 1221 (m), 1188 (m), 1169 (m), 1141 (w), 1125 (m), 1076 (m), 1046 (m), 1003 (s), 965 (vs), 927 (m), 909 (vs), 850 (m), 814 (m), 773 (m), 759 (m), 728 (m), 712 (w), 697 (m), 677 (w), 654 (w), 616 (w), 616 (w), 567 (m), 539 (w), 527 (m), 508 (w), 465 (w), 408 (w).

II. NMR spectra

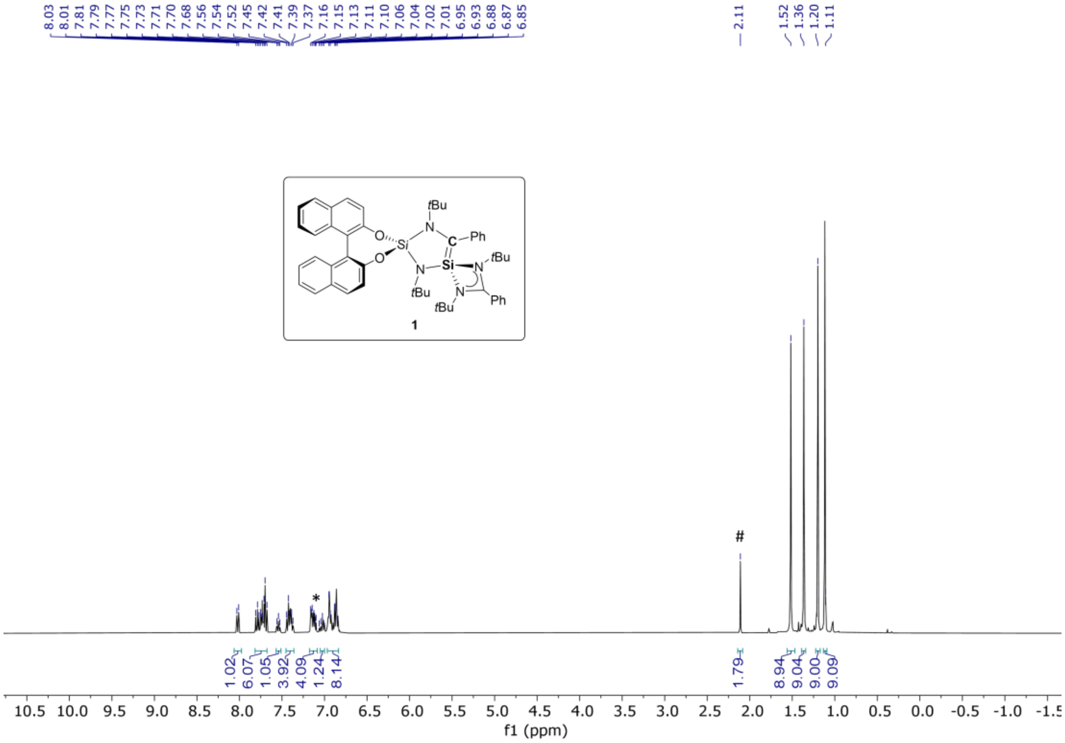

Figure S1. ^{1}H NMR spectrum of compound **1** in C_6D_6. *, residual protio solvent signal; #, CH_3 signals of toluene.

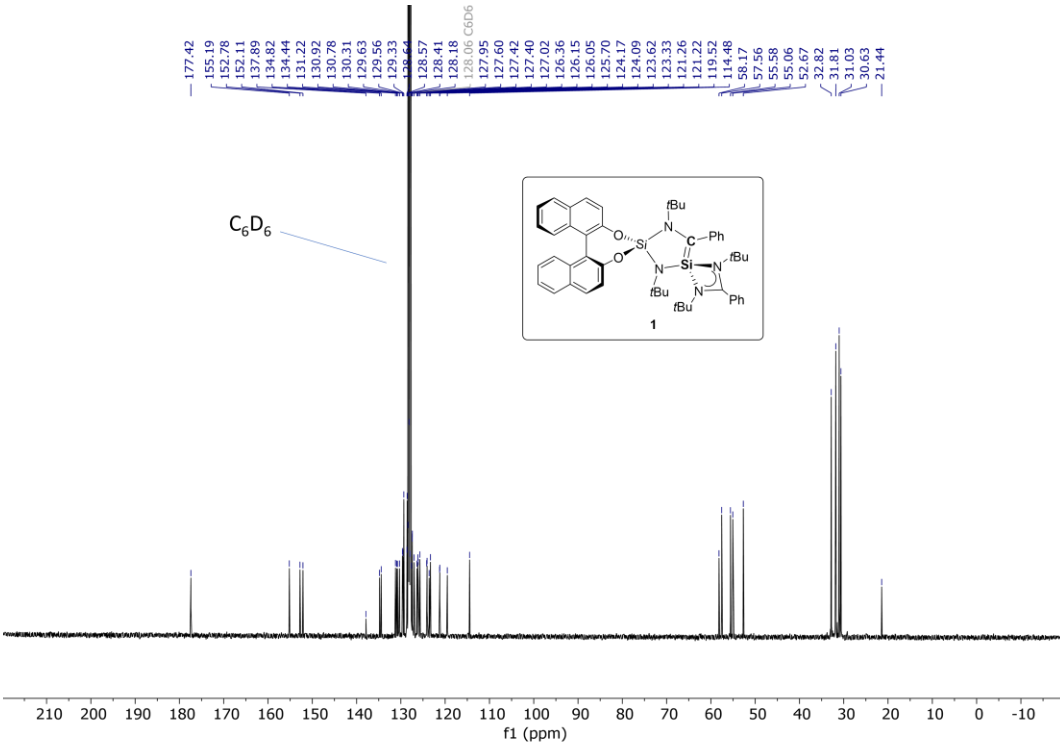

Figure S2. $^{13}C\{^{1}H\}$ NMR spectrum of compound **1** in C_6D_6.

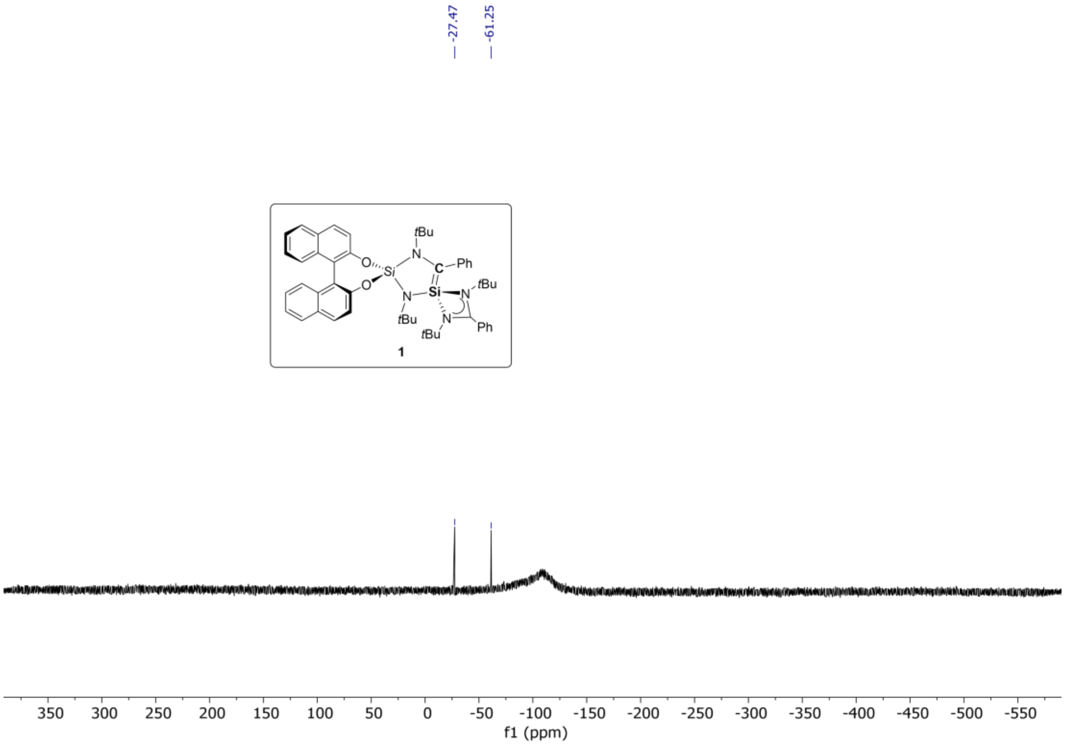

Figure S3. $^{29}Si\{^{1}H\}$ NMR spectrum of compound **1** in C_6D_6.

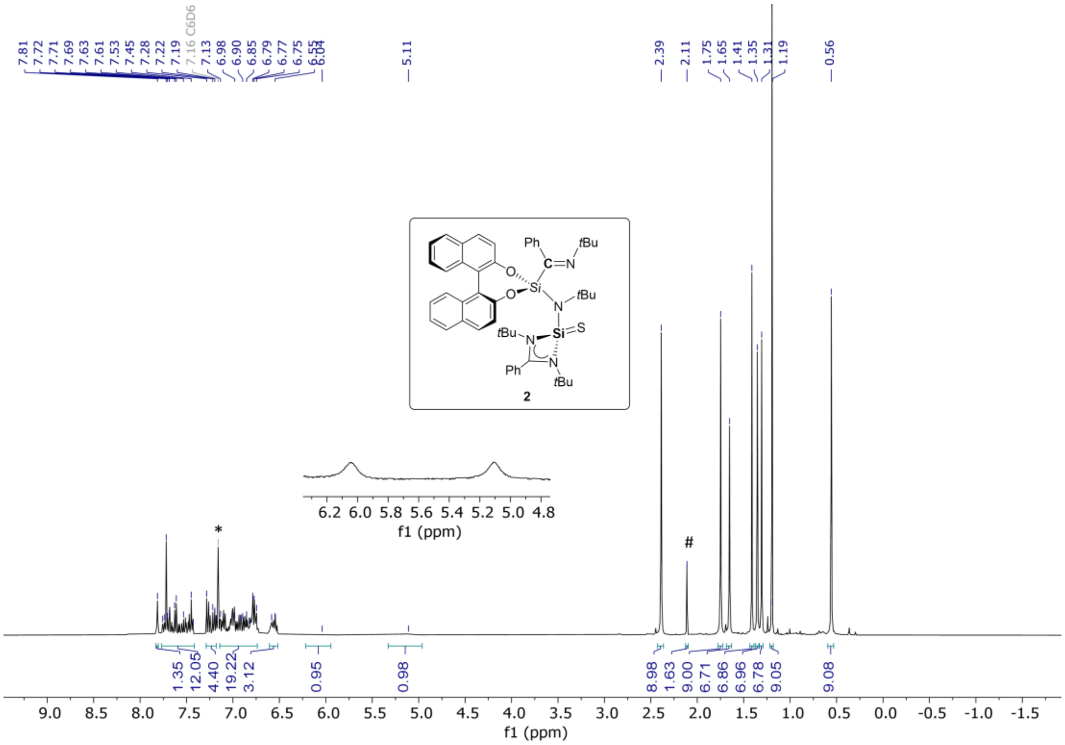

Figure S4. ^{1}H NMR spectrum of compound **2** in C_6D_6. *, residual protio solvent signal; #, CH_3 signals of toluene.

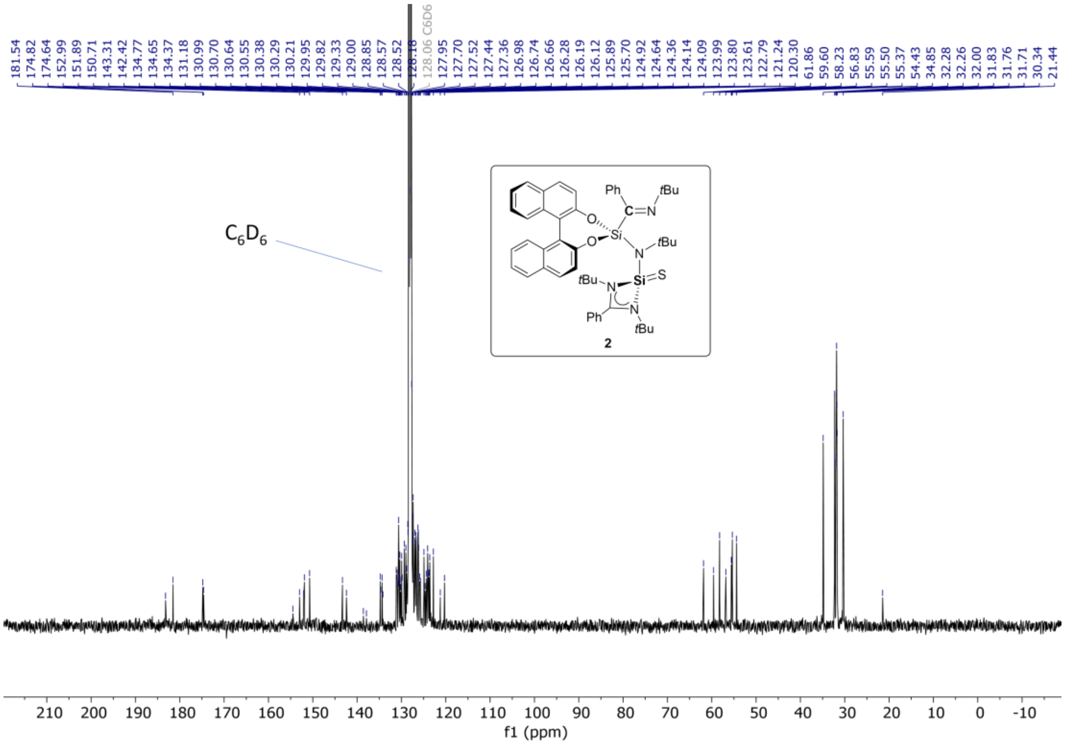

Figure S5. $^{13}C\{^1H\}$ NMR spectrum of compound **2** in C_6D_6.

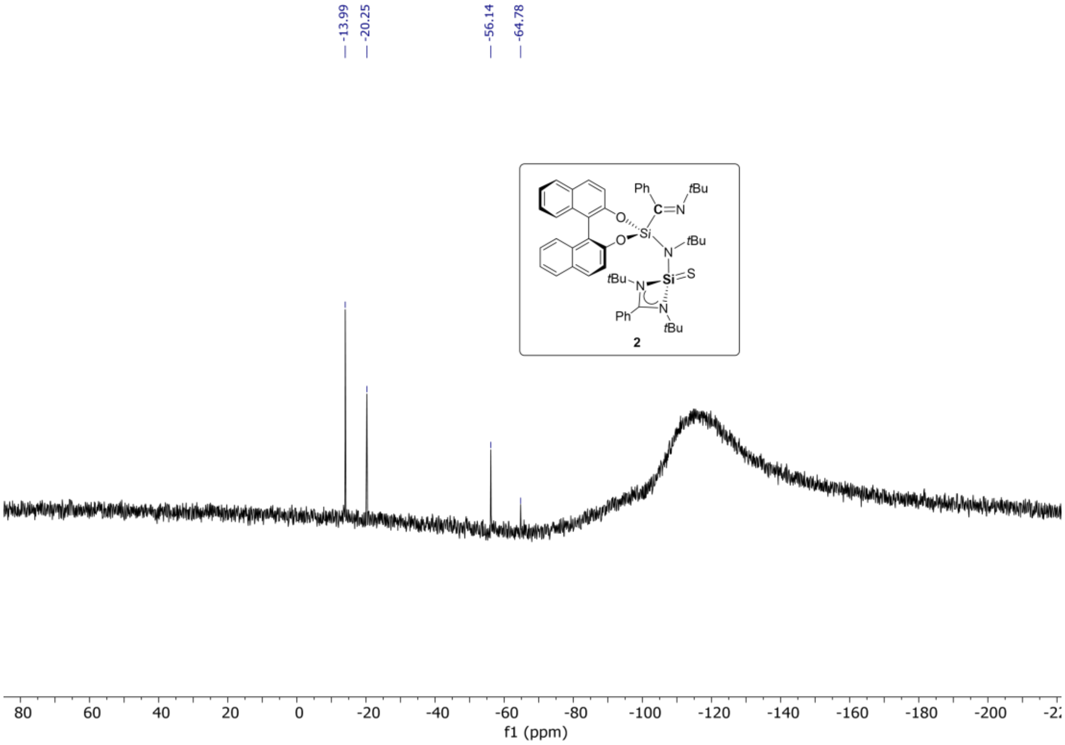

Figure S6. $^{29}Si\{^1H\}$ NMR spectrum of compound **2** in C_6D_6.

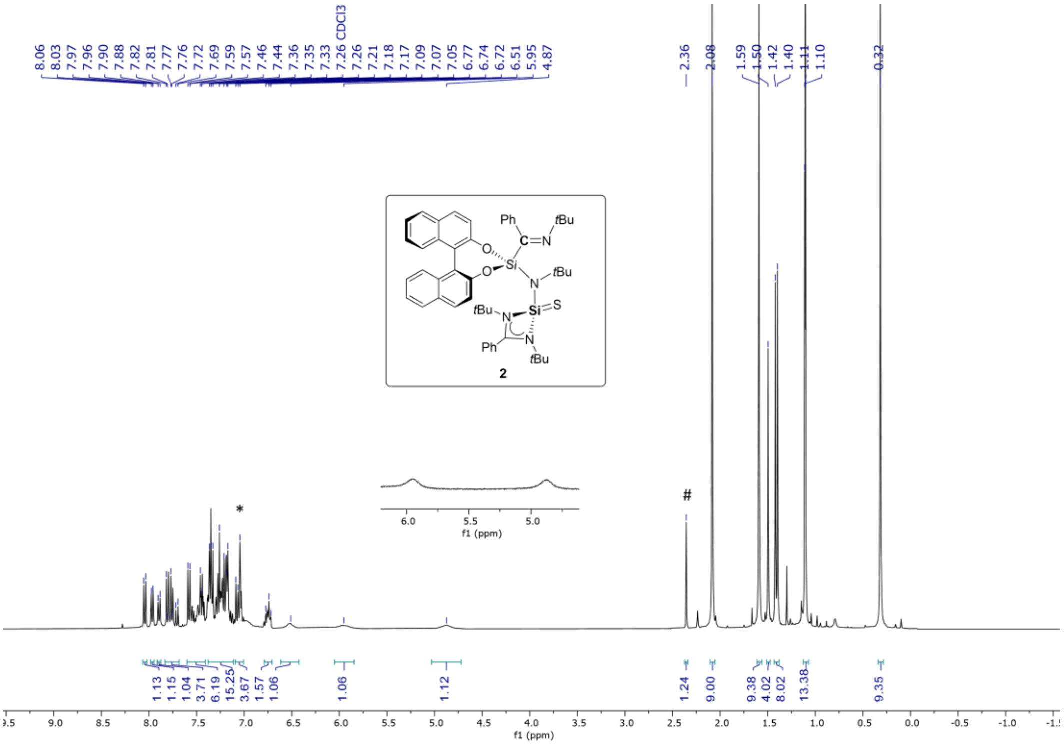

Figure S7. ^{1}H NMR spectrum of compound **2** in $CDCl_3$. *, residual protio solvent signal; #, CH_3 signals of toluene.

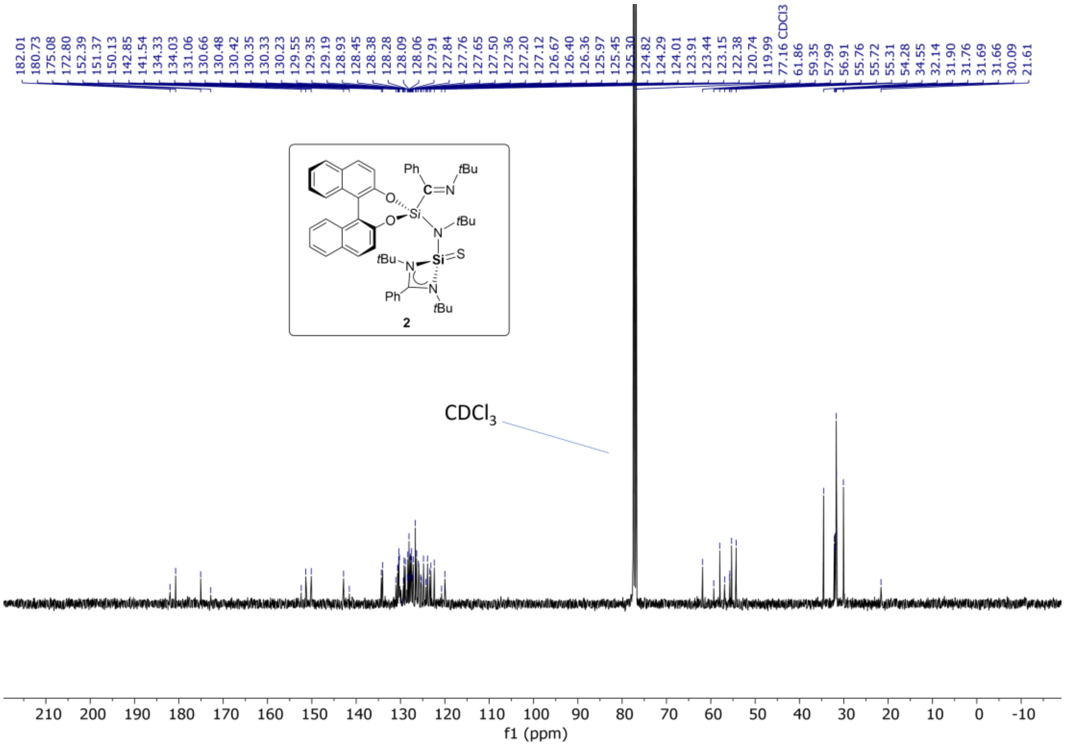

Figure S8. $^{13}C\{^{1}H\}$ NMR spectrum of compound **2** in $CDCl_3$.

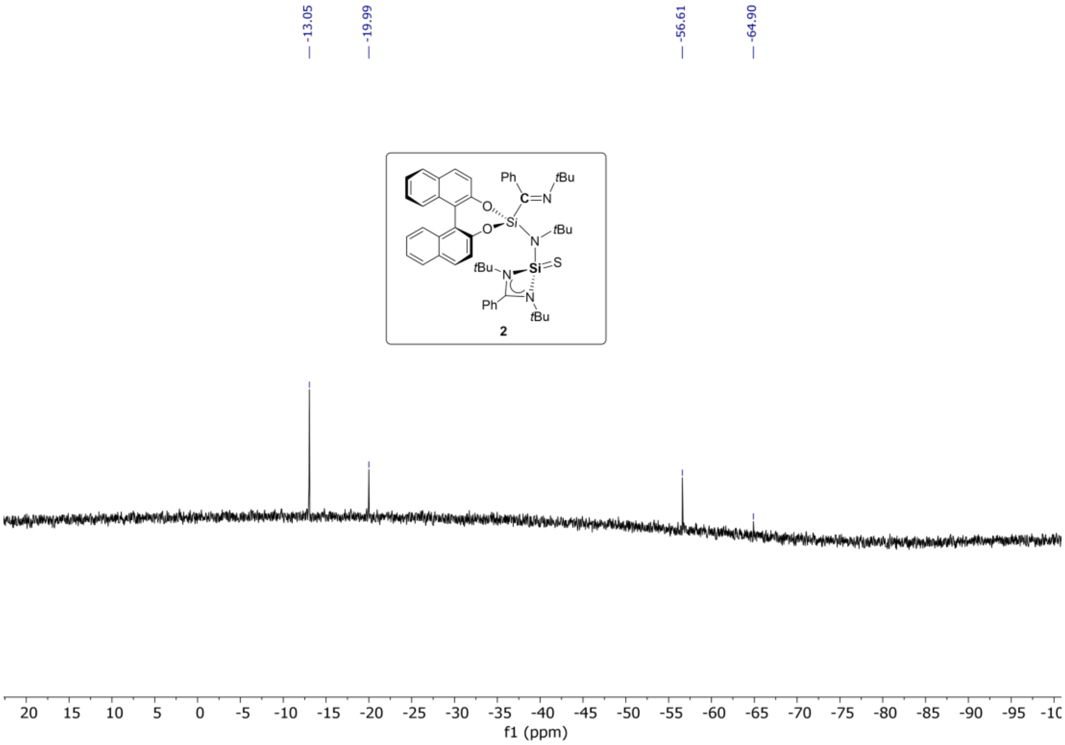

Figure S9. $^{29}Si\{^{1}H\}$ NMR spectrum of compound **2** in $CDCl_3$.

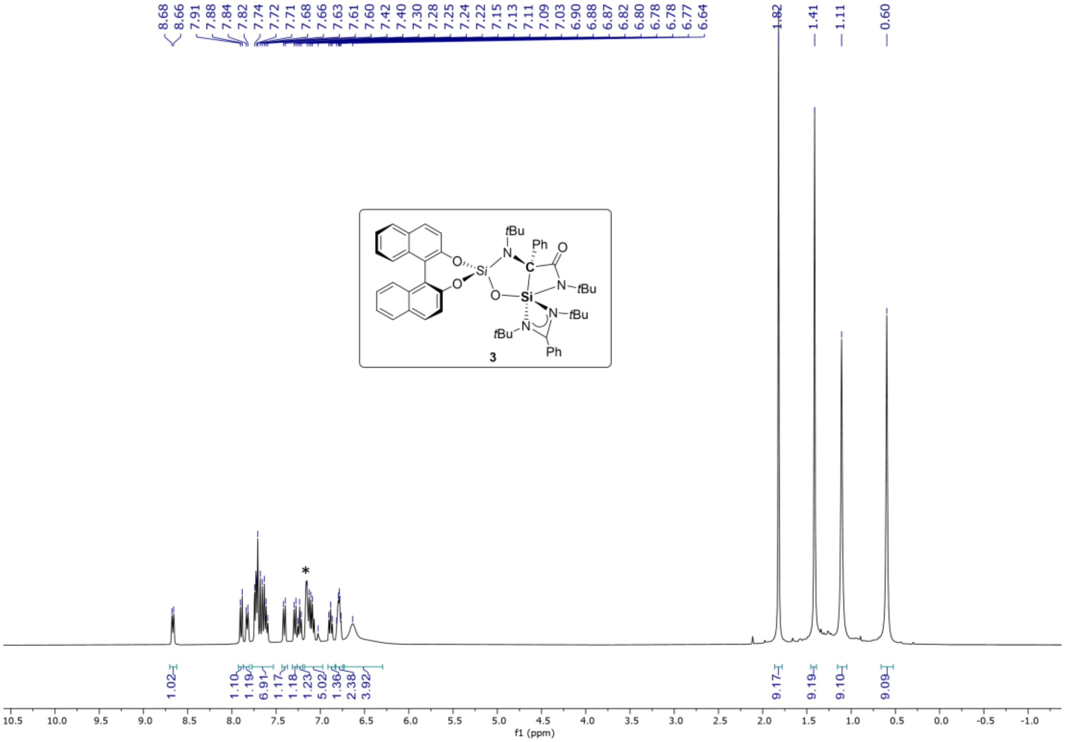

Figure S10. ^{1}H NMR spectrum of compound **3** in C_6D_6. *, residual protio solvent signal.

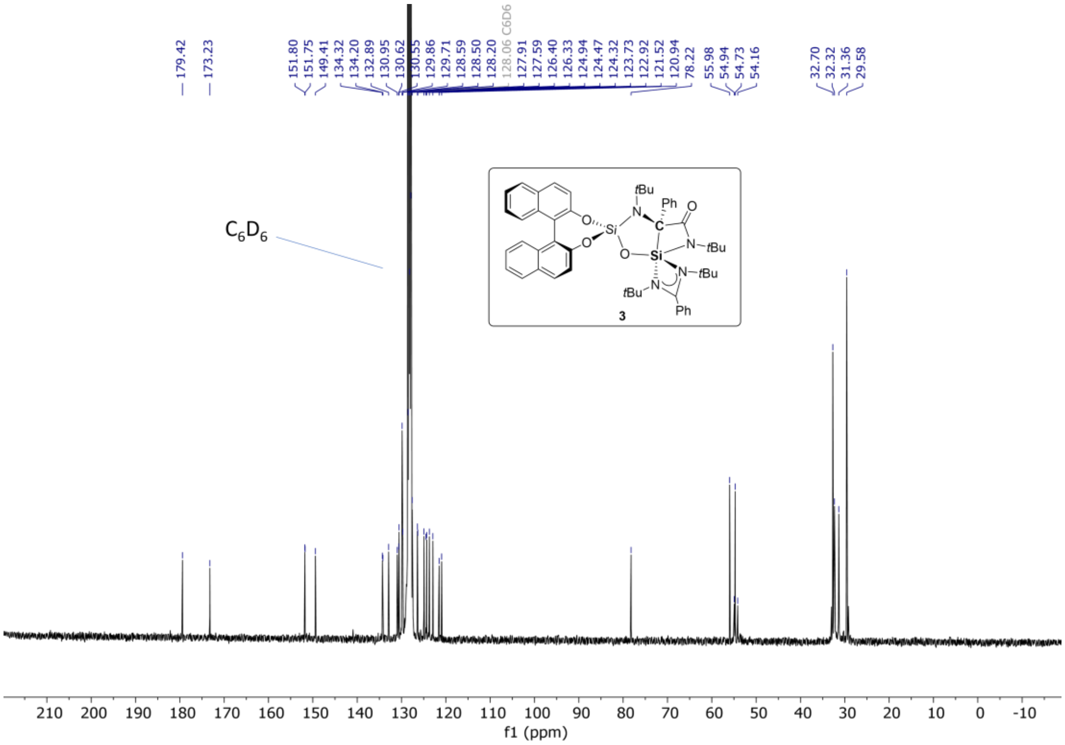

Figure S11. $^{13}C\{^1H\}$ NMR spectrum of compound **3** in C_6D_6. *, residual protio solvent signal.

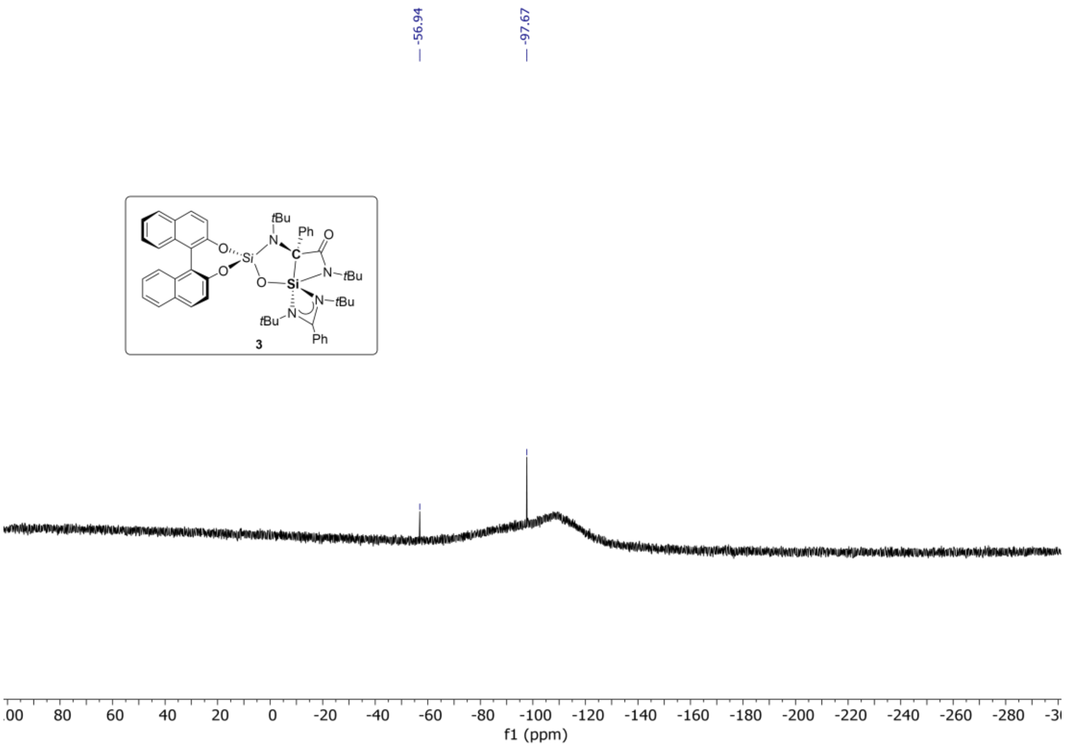

Figure S12. $^{29}Si\{^1H\}$ NMR spectrum of compound **3** in C_6D_6.

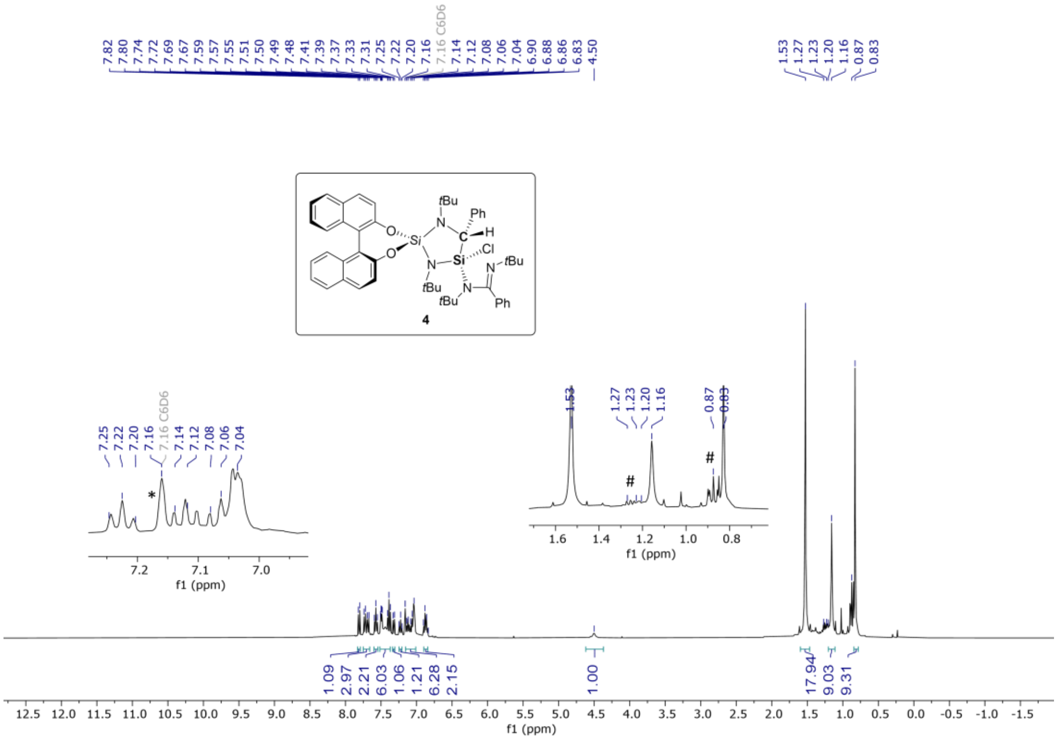

Figure S13. ^{1}H NMR spectrum of compound **4** in C_6D_6. *, residual protio solvent signal; #, signals of *n*-pentane.

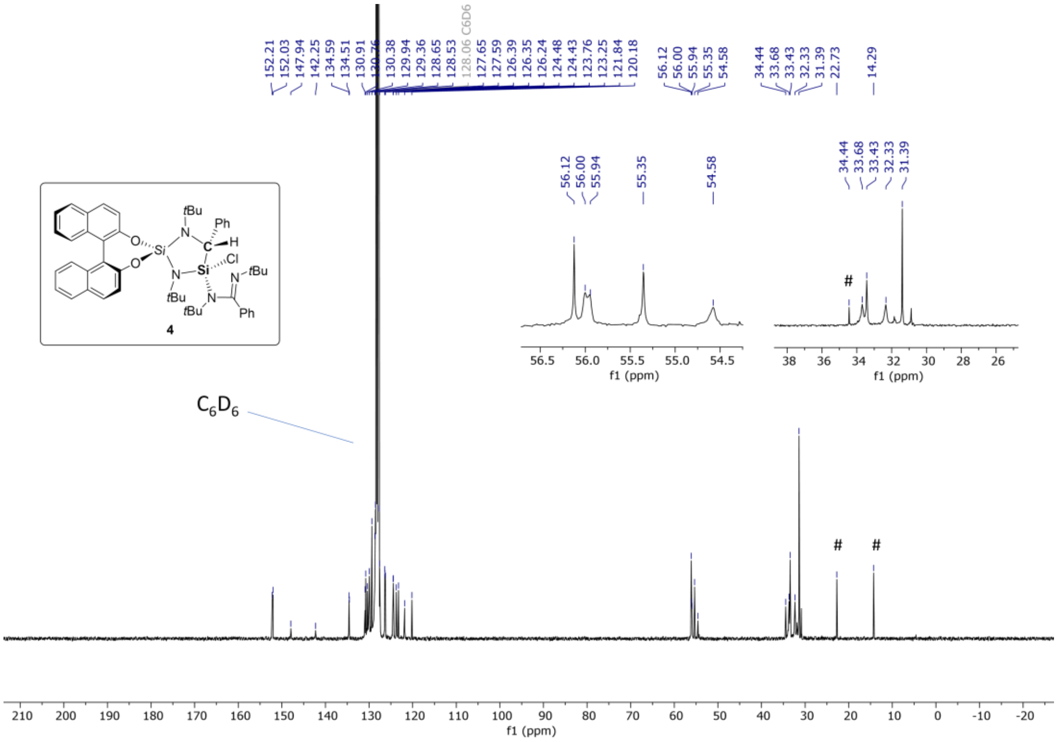

Figure S14. $^{13}C\{^{1}H\}$ NMR spectrum of compound **4** in C_6D_6. *, residual protio solvent signal; #, signals of *n*-pentane.

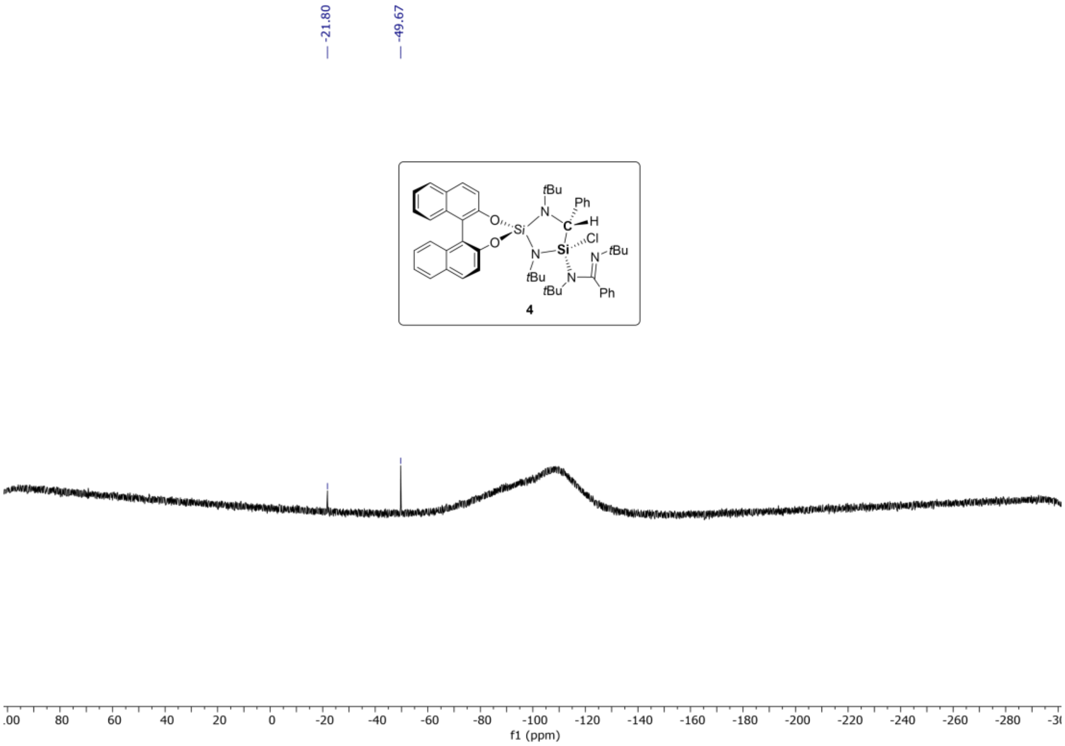

Figure S15. $^{29}Si\{^{1}H\}$ NMR spectrum of compound **4** in C_6D_6.

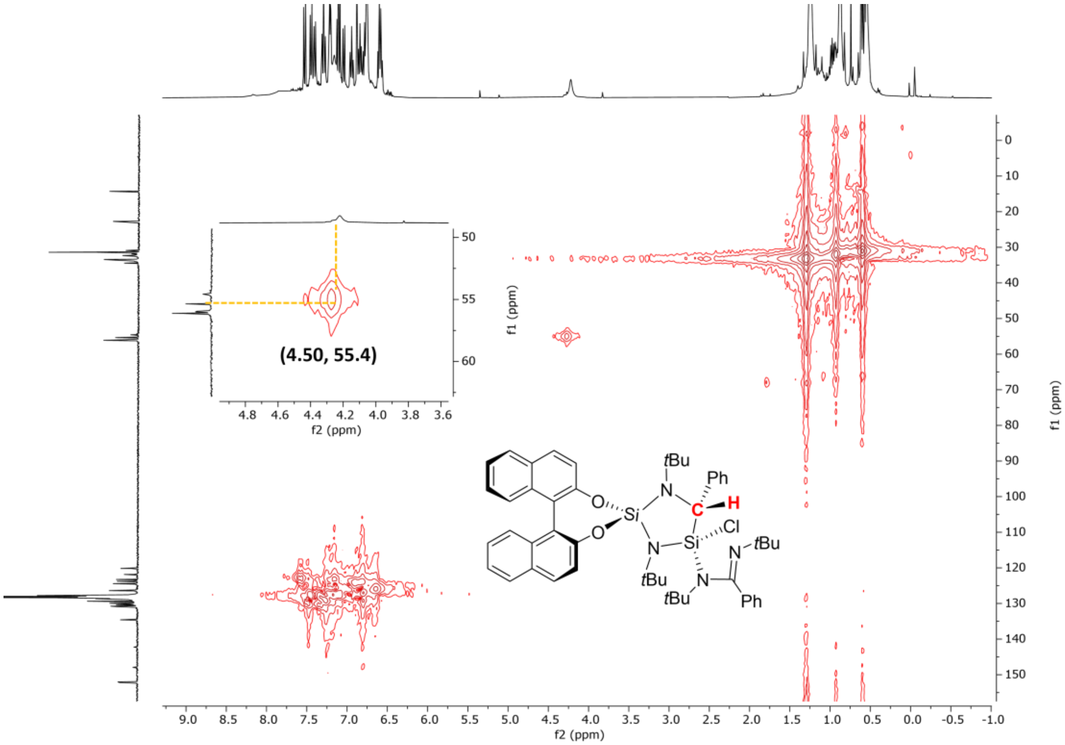

Figure S16. ^{1}H-^{13}C HMQC spectrum of compound **4** in CD_2Cl_2.

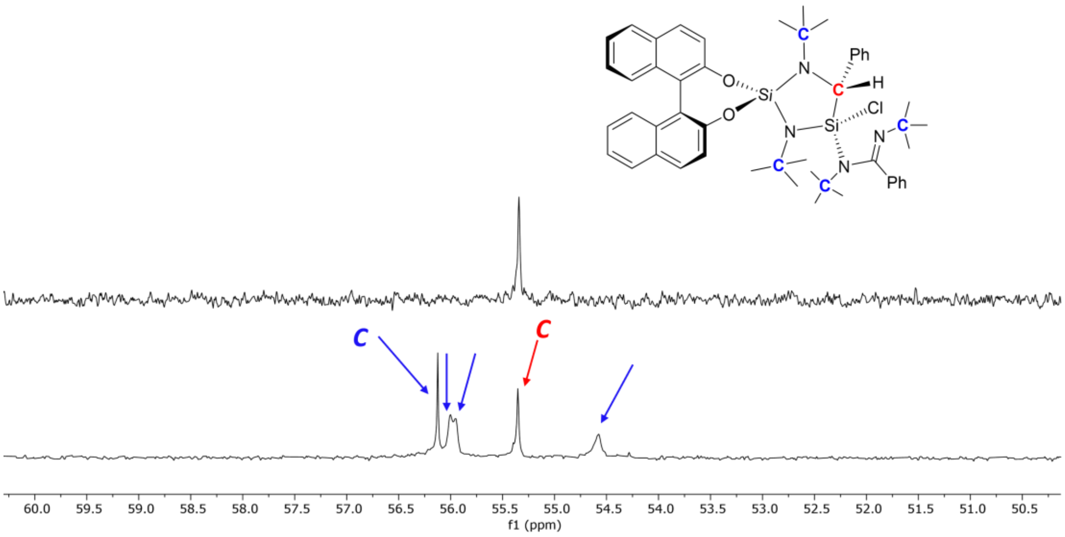

Figure S17. Selected regions of the ^{13}C-DEPT NMR (top) and ^{13}C{^{1}H} NMR of compound **4** in C_6D_6.

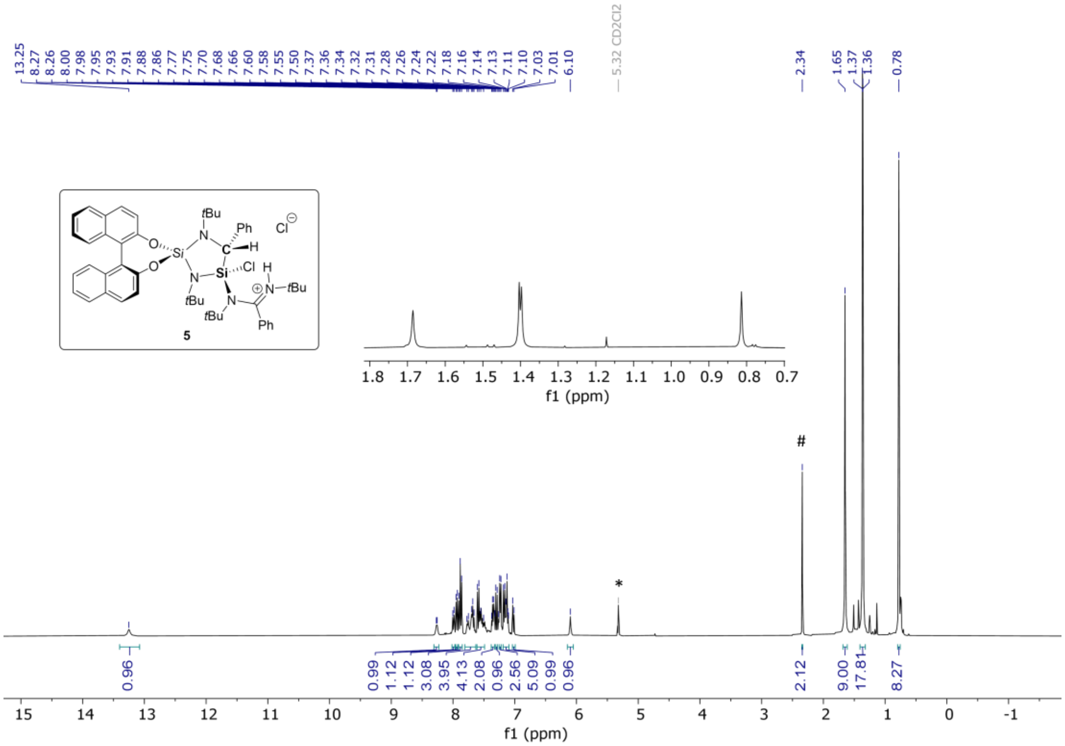

Figure S18. ^{1}H NMR spectrum of compound **5** in CD_2Cl_2. *, residual protio solvent signal; #, CH_3 signals of toluene.

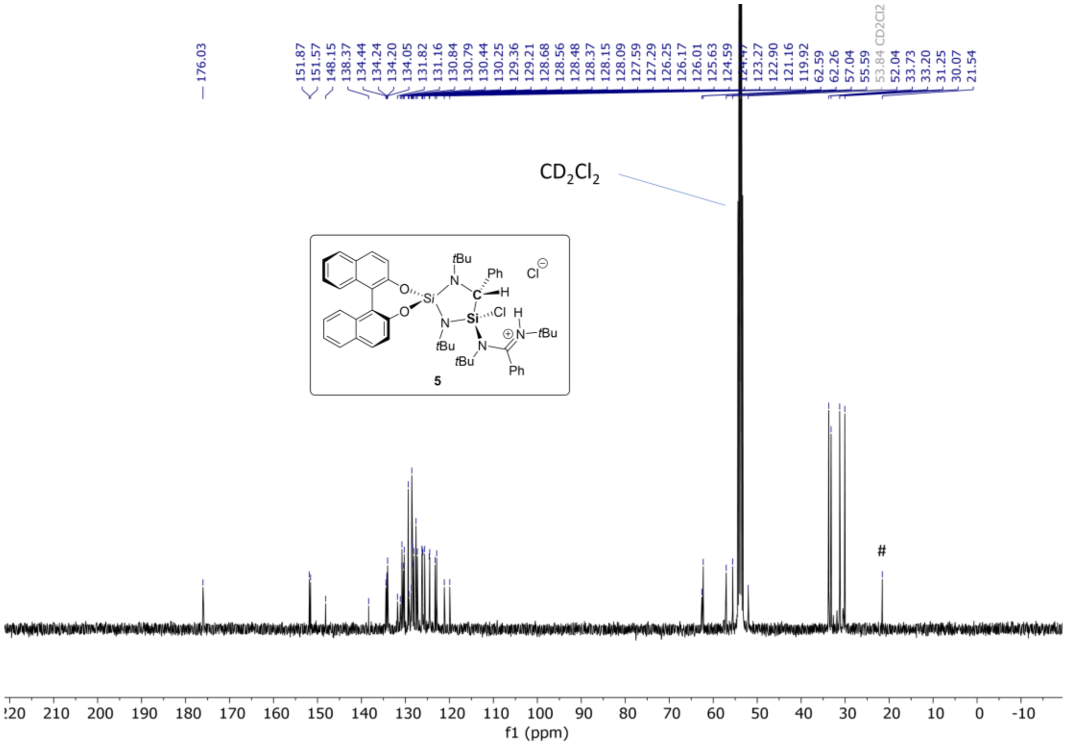

Figure S19. $^{13}C\{^1H\}$ NMR spectrum of compound **5** in CD_2Cl_2. *, residual protio solvent signal; #, signals of toluene.

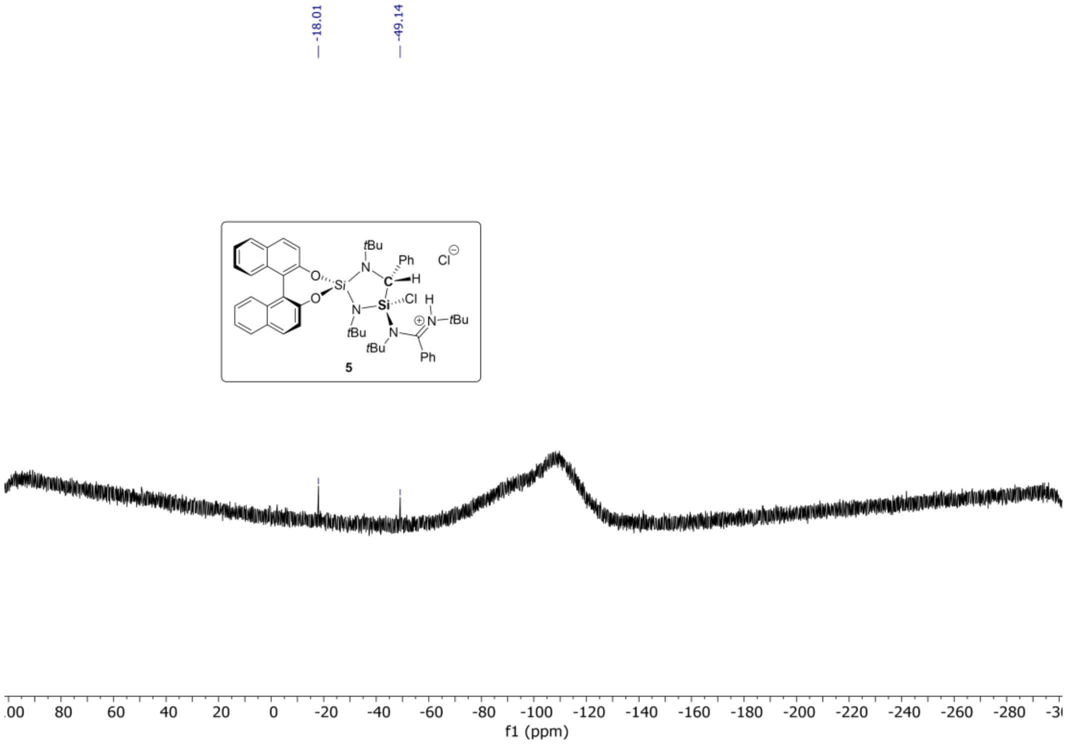

Figure S20. $^{29}Si\{^1H\}$ NMR spectrum of compound **5** in CD_2Cl_2.

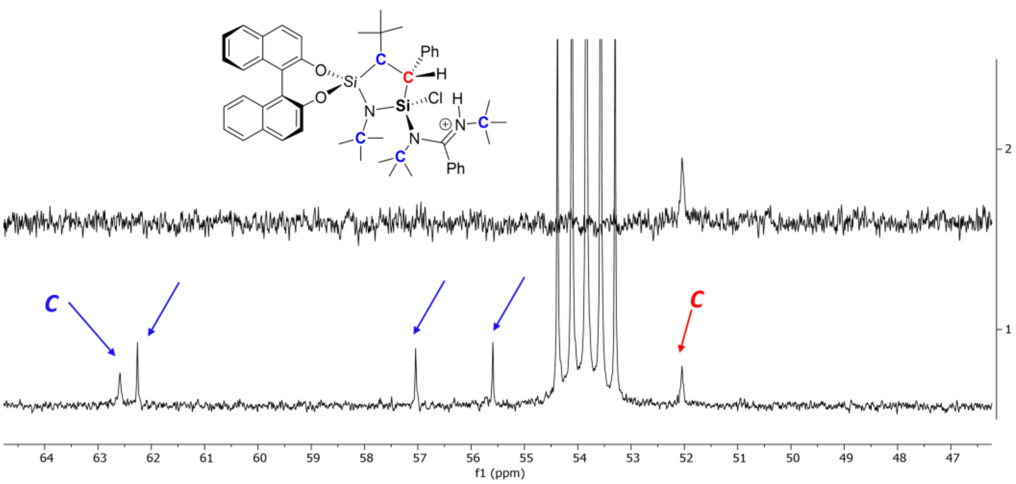

Figure S21. Selected regions of the ^{13}C-DEPT NMR (top) and ^{13}C{^{1}H} NMR of compound **5** in CD_2Cl_2.

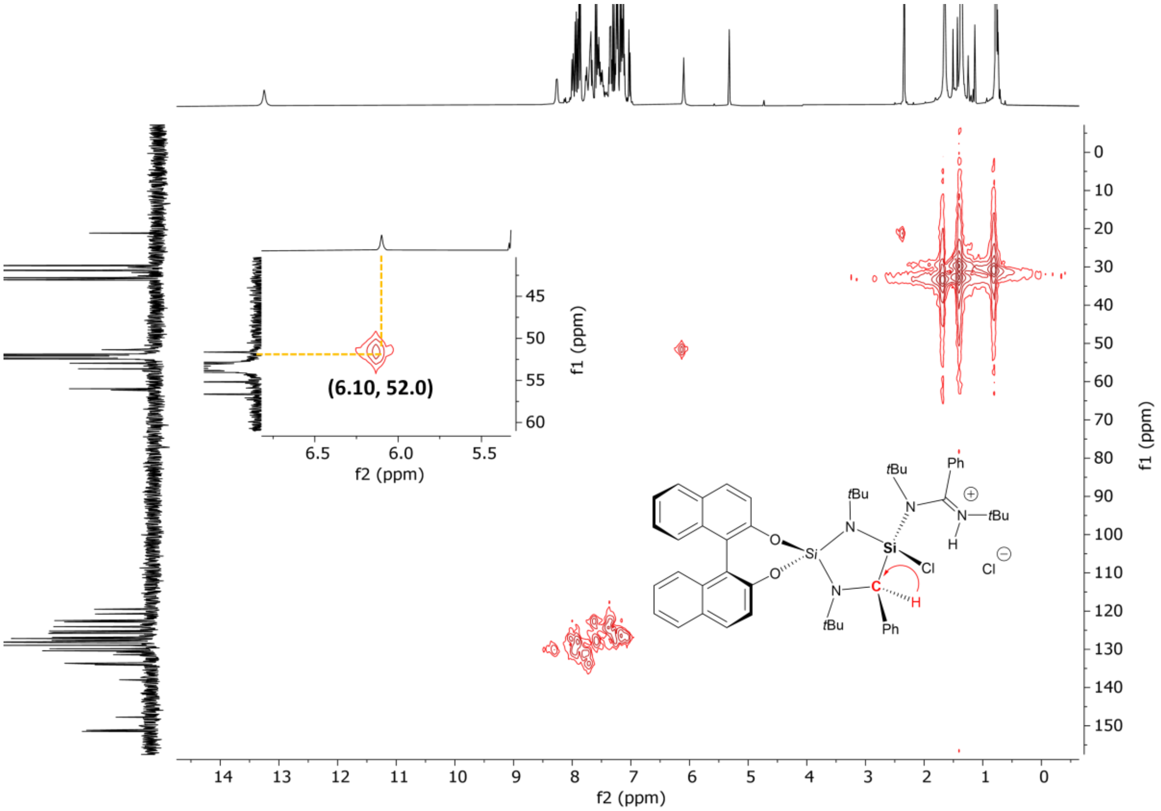

Figure S22. ^{1}H-^{13}C HMQC spectrum of compound **5** in C_6D_6.

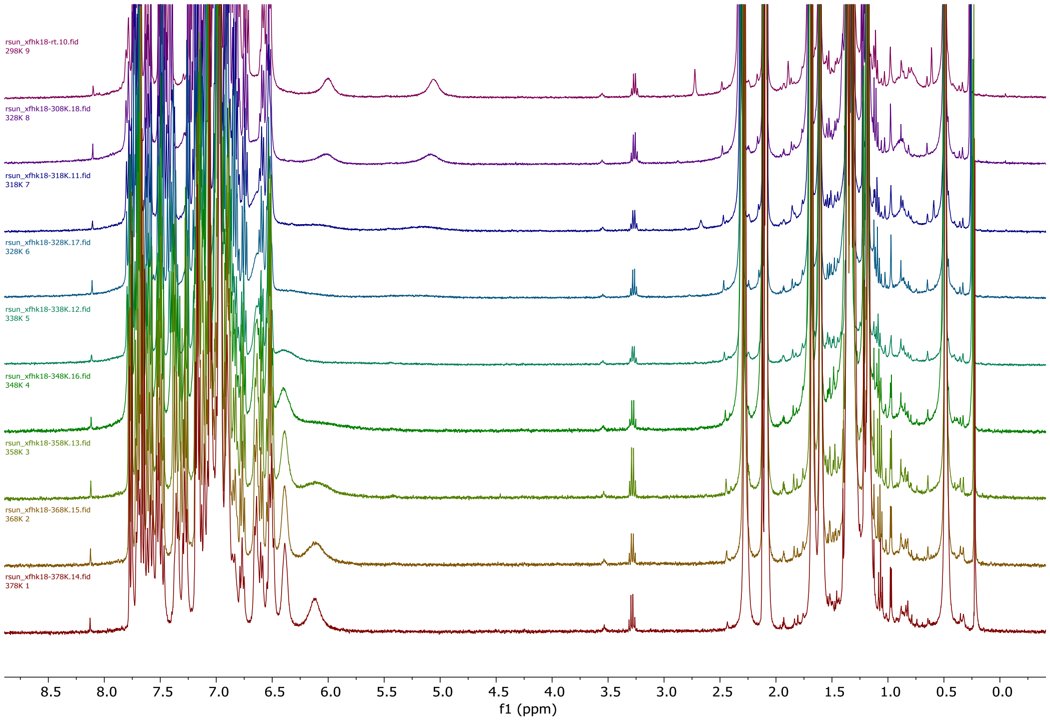

Figure S23. Variable temperature ^{1}H NMR studies (from 298 K to 378 K) of compound **2** in toluene-d_8.

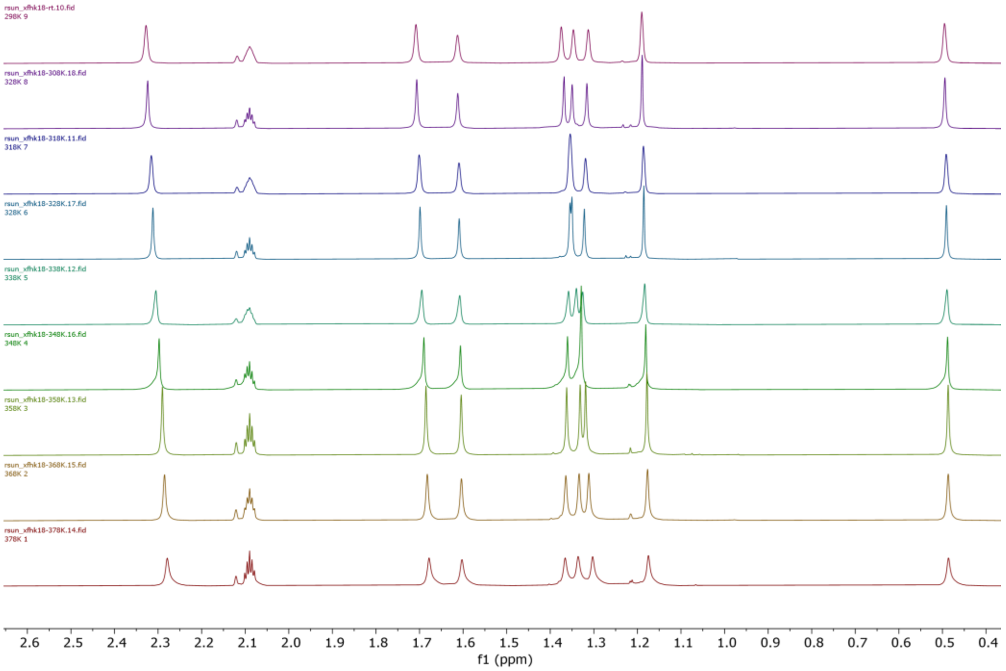

Figure S24. Selected region of the variable temperature ^{1}H NMR spectra of compound **2**.

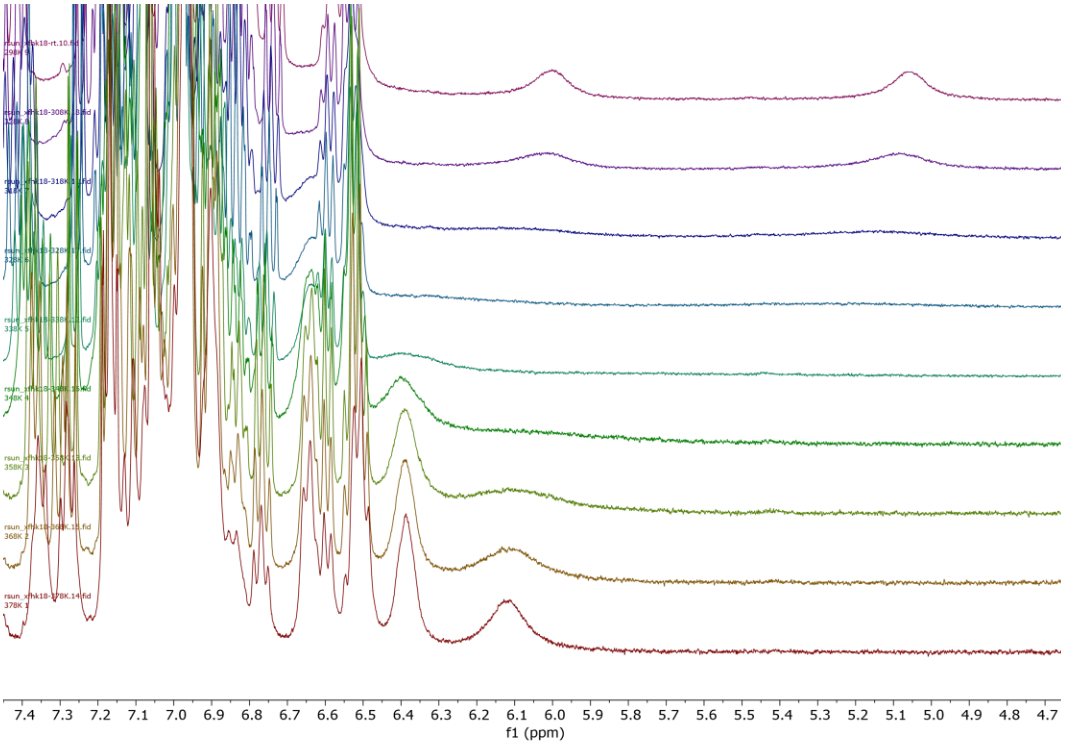

Figure S25. Selected region of the variable temperature ^{1}H NMR spectra of compound **2**.

IV. IR spectra

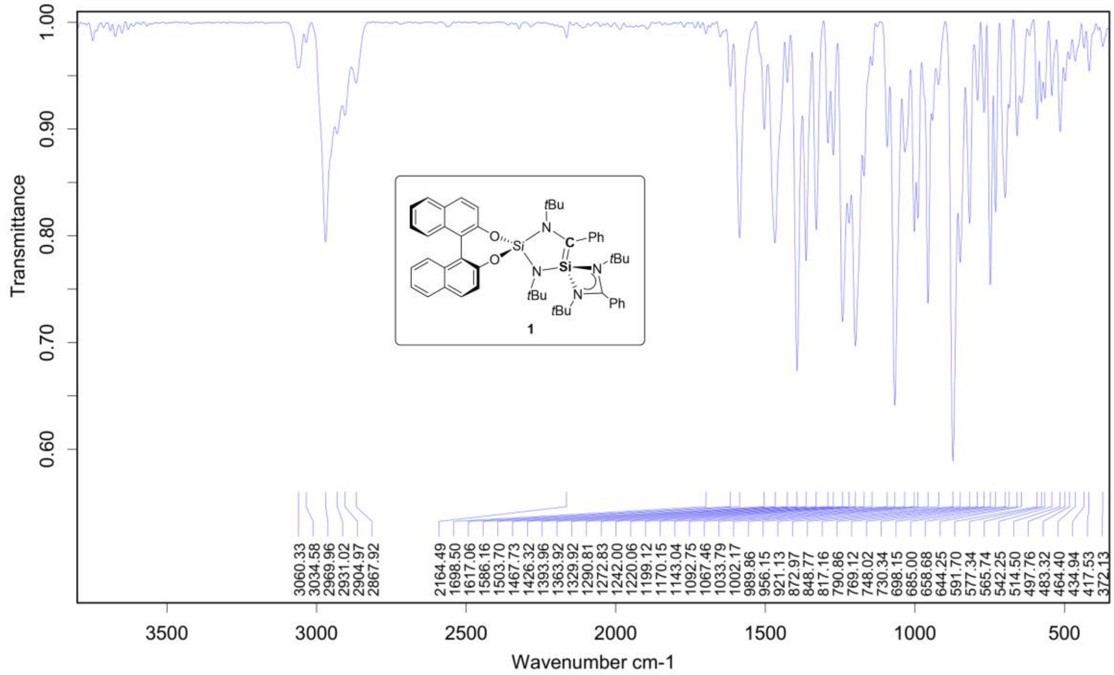

Figure S26. IR spectrum of compound **1**.

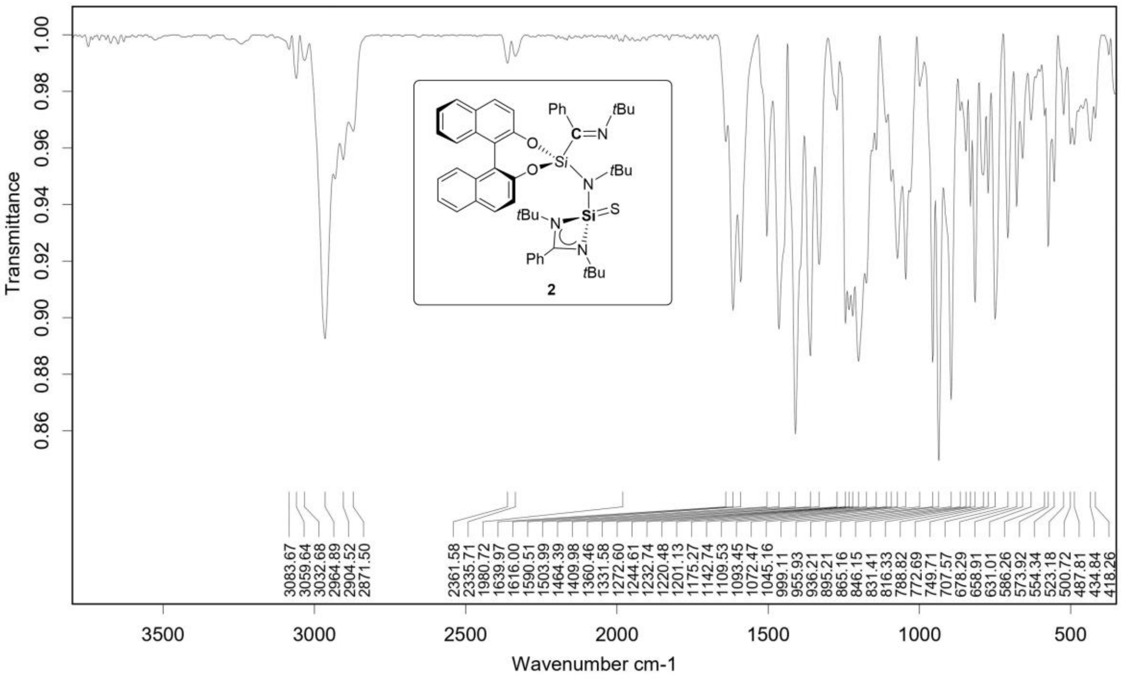

Figure S27. IR spectrum of compound **2**.

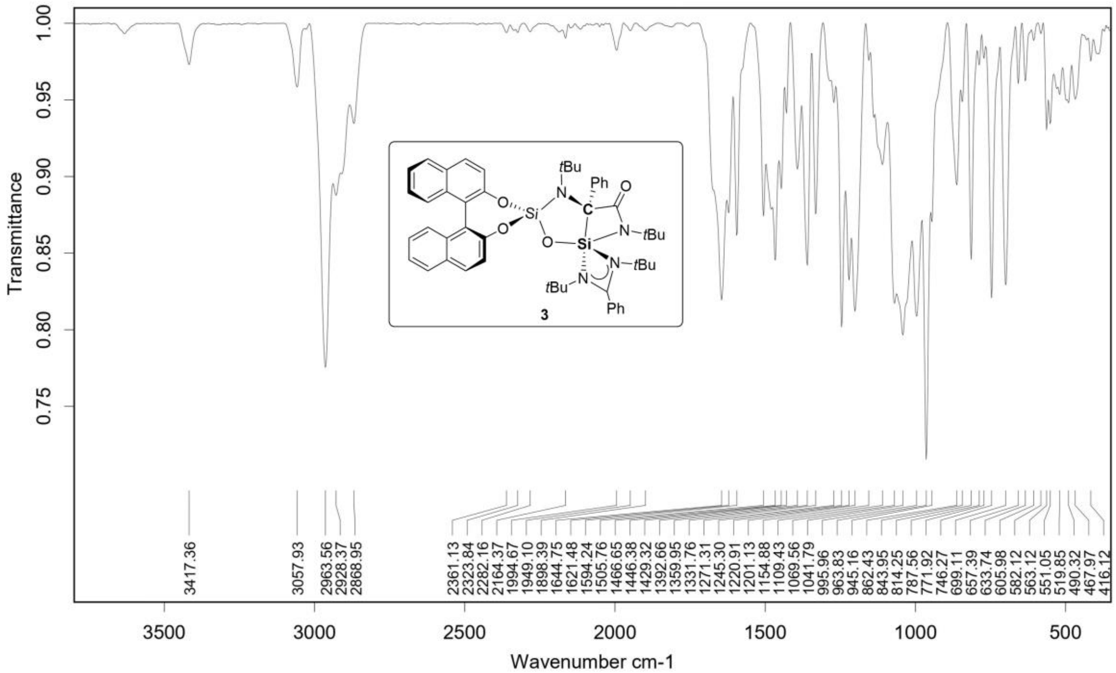

Figure S28. IR spectrum of compound **3**.

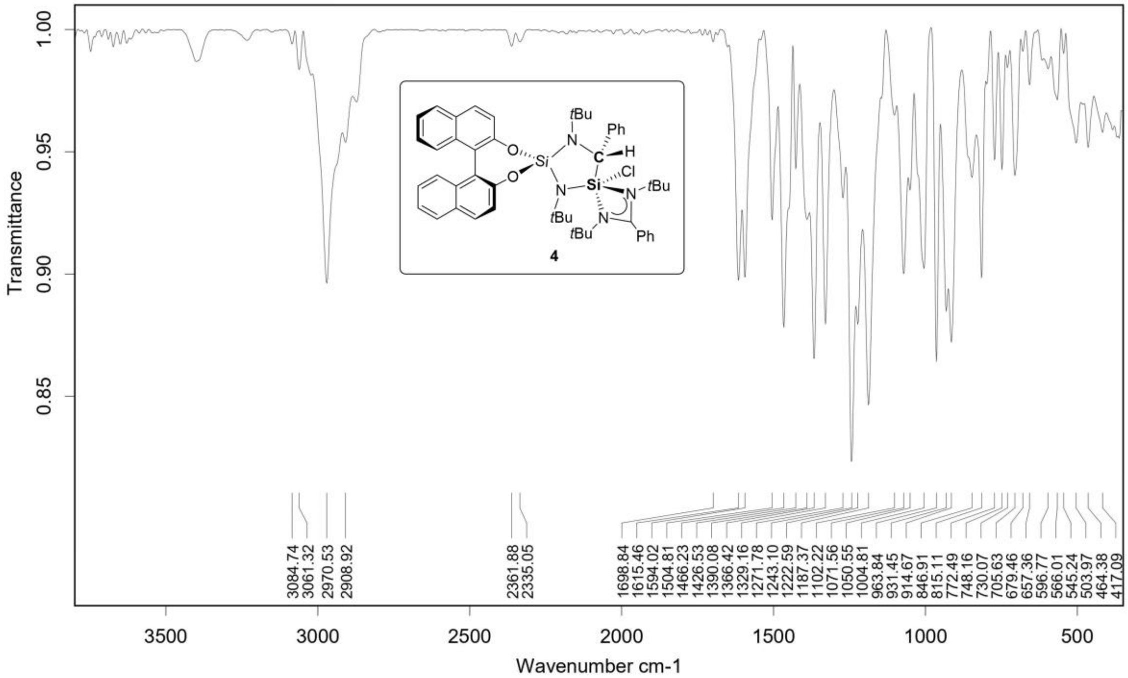

Figure S29. IR spectrum of compound **4**.

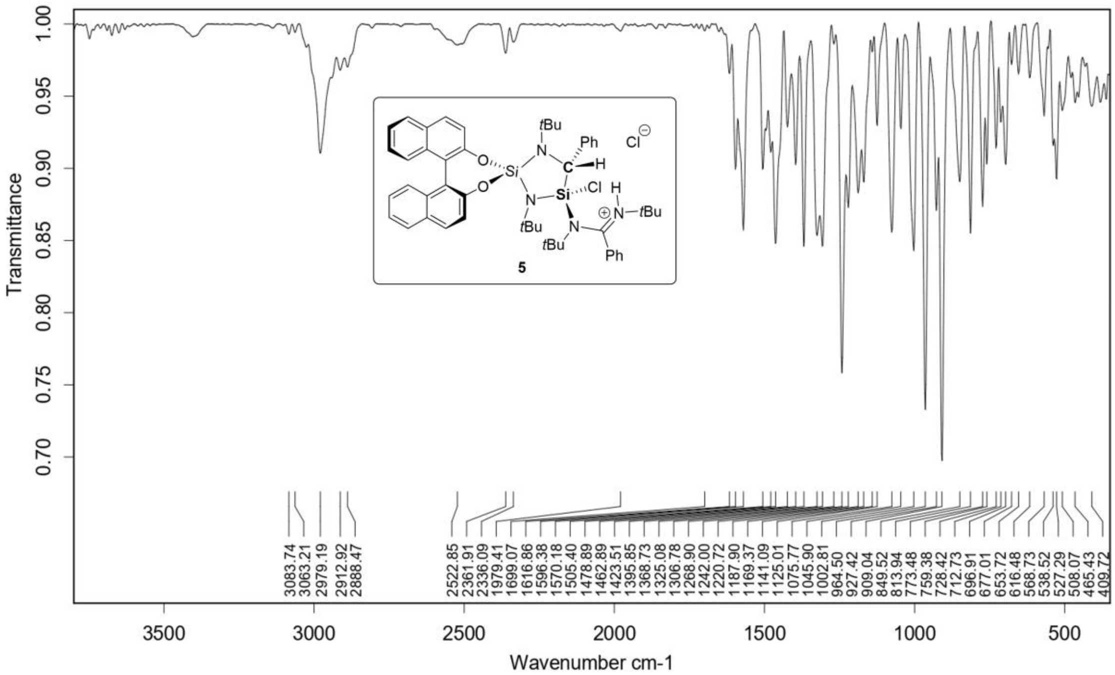

Figure S30. IR spectrum of compound **5**.

V. X-ray crystallography

V.1 General methods

Suitable crystals for the X-ray analysis of all compounds were obtained as described above. A suitable crystal was covered in mineral oil (Aldrich) and mounted on a glass fibre. The crystal was transferred directly to the cold stream of a STOE StadiVari (150 K) diffractometer. All structures were solved by using the program SHELXS/T[2,3] and Olex2.[3] The remaining non-hydrogen atoms were located from successive difference Fourier map calculations. The refinements were carried out by using full-matrix least-squares techniques on F^2 by using the program SHELXL.[3,4] The H-atoms were introduced into the geometrically calculated positions (SHELXL procedures) unless otherwise stated and refined riding on the corresponding parent atoms. In each case, the locations of the largest peaks in the final difference Fourier map calculations, as well as the magnitude of the residual electron densities, were of no chemical significance. Specific comments for each data set are given below. Summary of the crystal data, data collection and refinement for compounds are given in Table S1.

Crystallographic data for the structures reported in this paper have been deposited with the Cambridge Crystallographic Data Centre as a supplementary publication no. CCDC 2164389-2164392. Copies of the data can be obtained free of charge on application to CCDC, 12 Union Road, Cambridge CB21EZ, UK (fax: (+(44)1223-336-033; email: deposit@ccdc.cam.ac.uk).

<u>The following special comments apply to the models of the structures:</u>

- In the crystal structure of **1**, one co-crystallized toluene molecule (C108 – C121) is disordered over two positions with an occupancy ratio of 0.48/0.52.

- In the crystal structure of **3**, one Ph group (C18 – C23) is disordered over two positions with an occupancy ratio of 0.37/0.63, one co-crystallized C_6H_6 molecule (C58 – C63) is disordered over two positions with an occupancy ratio of 0.39/0.71.

- In the crystal structure of **5**, one *t*Bu group (C19 – C22) is disordered over two positions with an occupancy ratio of 0.5/0.5.

V.2. Summary of crystal data

Table S1. Crystal data, data collection and refinement for compounds **2** and **3**.

Compounds	**1**	**2**	**3**	**5**
Chemical formula	$C_{50}H_{58}N_4O_2Si_2$ (C_7H_8)	$C_{50}H_{58}N_4O_2SSi_2$	$C_{51}H_{58}N_4O_2Si_2$ $2(C_6H_6)$	$C_{50}H_{60}Cl_2N_4O_2Si_2$ (C_7H_8)
CCDC Number	2164389	2164390	2164391	2164392
Formula Mass	895.31	835.24	1003.41	968.23
Radiation type	GaKα	MoKα	MoKα	MoKα
Wavelength/Å	1.34143	0.71073	0.71073	0.71073
Crystal system	monoclinic	orthorhombic	monoclinic	monoclinic
a/Å	17.2697(3)	10.9530(3)	9.9464(5)	10.6167(11)
b/Å	17.0617(3)	13.6748(4)	16.8037(7)	19.3385(15)
c/Å	18.6755(3)	30.4373(9)	16.4798(10)	12.5092(12)
α/°	-	-	-	-
β/°	113.0460(10)	-	99.053(5)	98.792(8)
γ/°	-	-	-	-
Unit cell volume/Å^3	5063.57(14)	4558.9(2)	2720.1(2)	2538.1(4)
Temperature/K	150	100	100	100
Space group	$P2_1$	$P2_12_12_1$	$P2_1$	$P2_1$
Z	4	4	2	2
Absorption coefficient μ/mm^{-1}	0.633	0.167	0.117	0.222
No. of reflections measured	50074	27508	32418	22928
No. of independent reflections	16711	10144	14895	9861
R_{int}	0.0487	0.0328	0.0290	0.0831
Final R_1 values ($I > 2\sigma(I)$)	0.0420	0.0409	0.0417	0.0720
Final $wR(F^2)$ values ($I > 2\sigma(I)$)	0.1033	0.0956	0.0992	0.1855
Final R_1 values (all data)	0.0571	0.0517	0.0509	0.0799
Final $wR(F^2)$ values (all data)	0.1084	0.1005	0.1091	0.1928
Goodness of fit on F^2	0.976	1.042	1.052	1.034
Flack parameter	0.014(16)	0.10(5)	-0.04(4)	-0.05(11)

V.3 Crystal structures

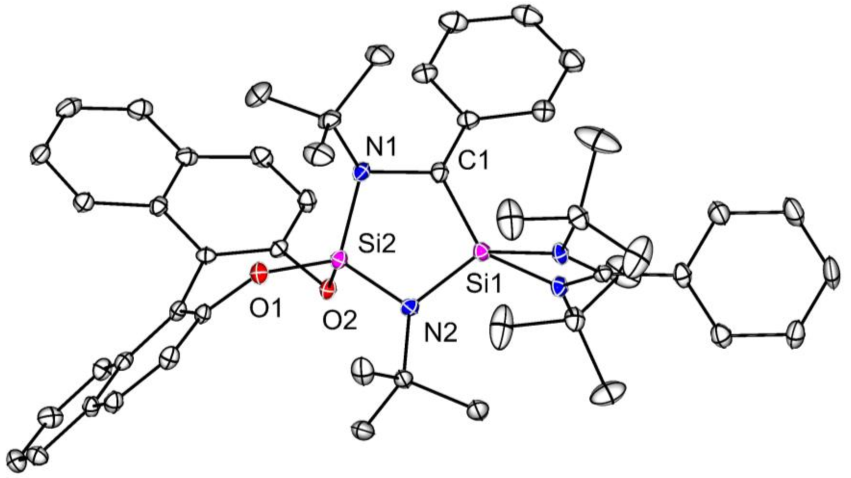

Figure S31. Molecular structure of the compound **1** in the solid state with thermal ellipsoids at the 25% probability level. Non-coordinating solvent molecules and H atoms are omitted for clarity. Selected bond distances [Å] and angles [°]: Si1-C1 1.759(3), Si1-N2 1.719(2), C1-N1 1.488(4), Si2-N1 1.699(2), Si2-N2 1.737(3), Si2-O1 1.655(2), Si2-O2 1.655(2); S1-C1-N1 114.4(2), C1-N1-Si2 102.7(2), N1-Si2-N2 107.43(13), Si2-N2-Si1 102.61(12), N2-Si1-C1 102.22(14), O1-Si2-O2 104.11(11).

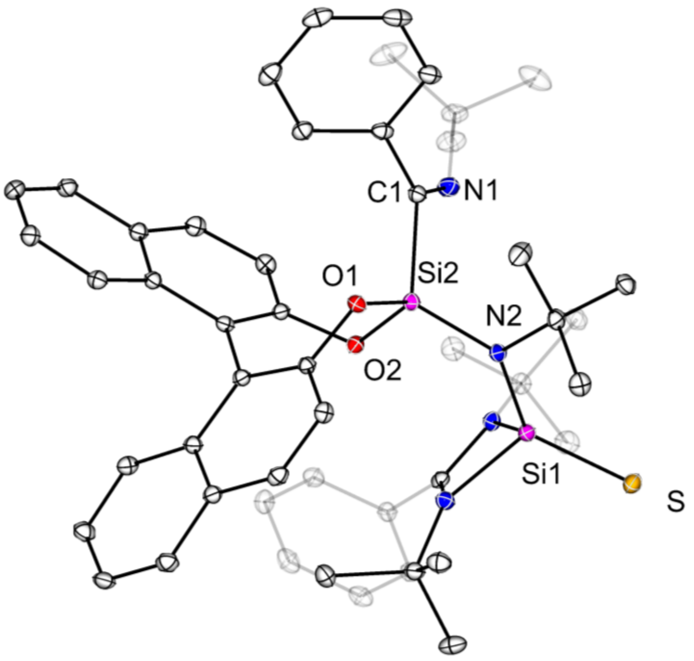

Figure S32. Molecular structure of the compound **2** in the solid state with thermal ellipsoids at the 25% probability level. H atoms are omitted for clarity. Selected bond distances [Å] and angles [°]: Si1-S 2.0006(10), Si1-N2 1.753(2), Si2-C1 1.900(3), Si2-N2 1.720(2), Si2-O1 1.654(2), Si2-O2 1.662(2), C1-N1 1.276(4); N1-C1-Si2 110.0(2), O1-Si2-O2 103.85(10), C1-Si2-N2 117.72(12), Si2-N2-Si1 125.66(13), N2-Si1-S 123.38(8).

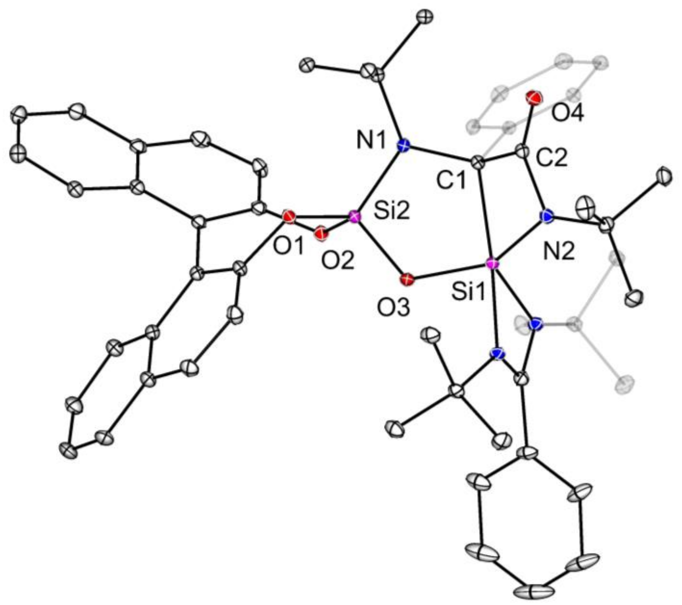

Figure S33. Molecular structure of the compound **3** in the solid state with thermal ellipsoids at the 25% probability level. Non-coordinating solvent molecules and H atoms are omitted for clarity. Selected bond distances [Å] and angles [°]: Si1-C1 2.018(2), Si1-N2 1.788(2), Si1-O3 1.678(2), Si2-O1 1.644(2), Si2-O2 1.647(2), Si2-O3 1.608(2), Si2-N1 1.683(2), C1-C2 1.526(3), C1-N1 1.482(3), C2-O4 1.213(3), C2-N2 1.375(3); O1-Si2-O2 105.58(9), Si1-O3-Si2 119.85(10), Si2-N1-C1 110.76(15), N1-C1-C2 115.3(2), N1-C1-Si1 109.08(15), C1-C2-N2 103.1(2).

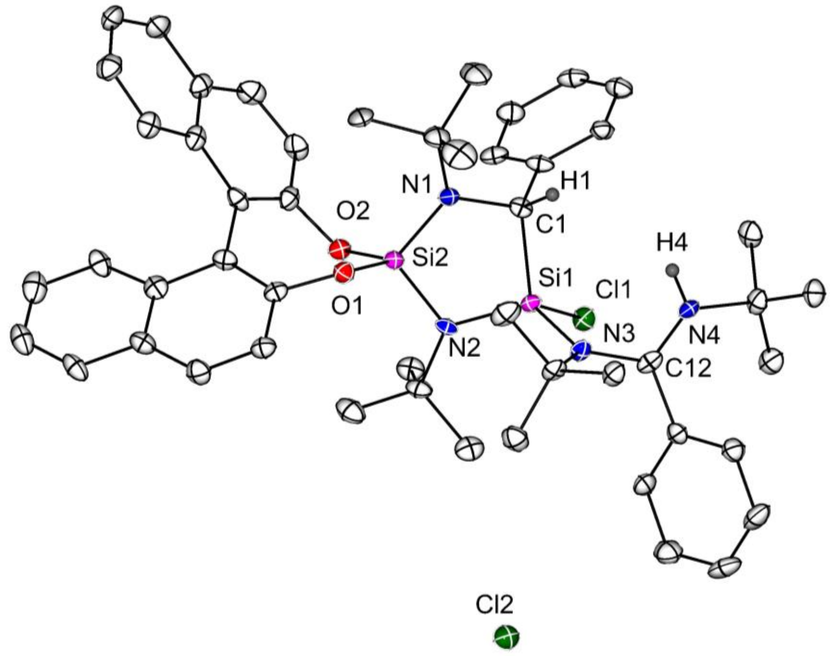

Figure S34. Molecular structure of the compound **5** in the solid state with thermal ellipsoids at the 25% probability level. Non-coordinating solvent molecules and H atoms are omitted for clarity. Selected bond distances [Å] and angles [°]: Si1-Cl1 2.056(2), Si1-C1 1.900(6), Si1-N2 1.704(6), Si1-N3 1.770(6), Si2-N1 1.687(6), Si2-N2 1.718(6), Si2-O1 1.631(5), Si2-O2 1.641(5), N1-C1 1.479(8), N3-C12 1.372(9), N4-C12 1.283(9); C1-Si1-N2 100.5(3), C1-Si1-Cl1 109.2(2), C1-Si1-N3 117.3(3), Cl1-Si1-N2 110.5(2), Cl1-Si1-N3 102.5(2), N2-Si1-N3 116.9(3), Si2-N1-C1 114.5(4), N1-C1-Si1 106.1(4), Si1-N2-Si2 109.7(3), O1-Si2-O2 103.9(3), N3-C12-N4 119.0(6).

VI. Quantum chemical calculations

All computations were performed using Gaussian16[5] utilizing the PBE1PBE level of theory, Def2SVP basis sets and empirical dispersion correction (GD3). No solvent corrections were applied. All optimized molecular structures were checked to be minima on the energy hypersurface and possess no imaginary vibrational frequencies.

VI.1. Silene

Table S2: Computed data for **1**.

	E [a.u.]	$\delta(^{1}H)$	$\delta(^{29}Si)$	$\delta(^{1}H$, phenyl)	$\delta(^{13}C)$
**1**	-2883.358683		-31, -62	6.48, 6.88, 6.26, 7.03, 7.44	172.4, 58.4

VI.2. S reaction

Table S3: Computed data of the reaction of **1** with sulfur.

	G [a.u.]	δ(^{29}Si)calc	δ(^{29}Si)obs	δ(^{1}H, phenyl)	δ(^{13}C)	
**1**	-2883.358683	-31, -62	-27, -61	6.48, 6.88, 6.26, 7.03, 7.44	172.4, 58.4	
**2A**	-3281.296438	-28, -104	-28, -107			114.5
**2B**	-3281.302510	-51, -52	-48, -52			90.7
**2C-1**	-3281.230678	-22, -40	-16, -40			287.1
**2C-2**	-3281.296704	-13, -55	-16, -40			
**2C-3**	-3281.230678	-29, -48	-16, -40			287.1
**2´**	-3281.331698	-19, -53	-20, -65	7.79, 7.10, 7.22, 7.24, 7.38	172.6, 171.5	21.9
**2**	-3281.340046	-17, -56	-14, -56	3.96, 5.89, 6.73, 6.98, 7.01	184.7, 171.5	0.0

The geometry optimization of the hypothetical isomers of **2** with the imine-Ph and imine-*t*Bu groups in *trans* position to each other was attempted but converged to the respective *cis* isomers **2** and **2’.**

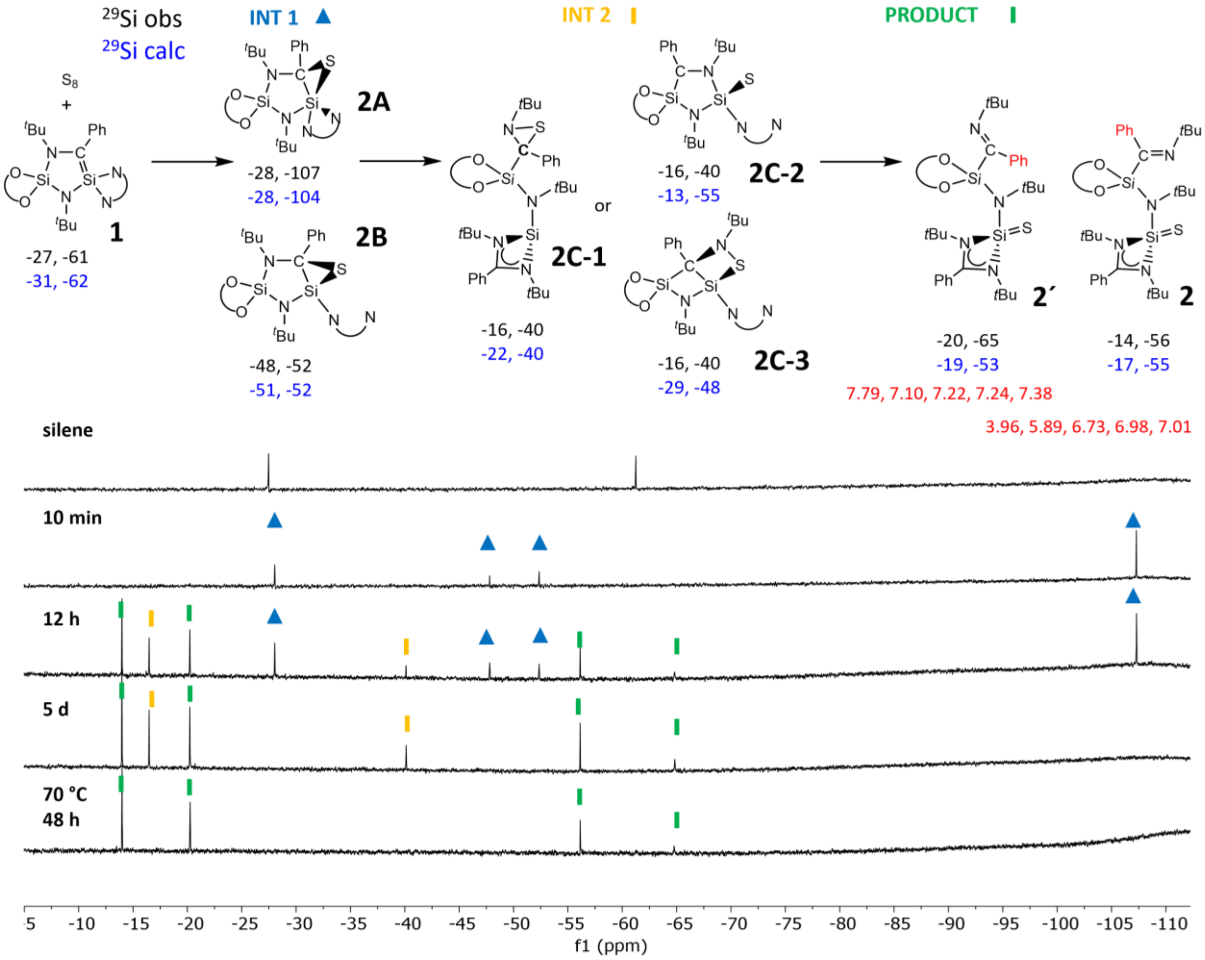

Figure S35. Computed (top) and ^{29}Si NMR data (bottom) of the reaction of **1** with sulfur.

VI.3. CO_2 reaction

Table S4: Computed data for **3**.

	E [a.u.]	δ(^{29}Si)	δ(^{1}H, phenyl)	δ(^{13}C)
**3**	-3071.667229	-56, -94	7.49, 7.12, 7.00, 7.50, 8.57	174.0, 169.8, 75.1

Table S5: Computed data for the methyl model (**3**$^{\#}$) of **3**.

methyl model	G [a.u.]	ΔG [a.u.]	ΔG [kJ/mol]
CO_2	-188.252		
1$^{\#}$	-1416.79		
**I$_0$$^{\#}$**	-1605.04	0	0.0
**TS$_1$$^{\#}$**	-1605.04	0.005927	15.6
**I$_1$$^{\#}$**	-1605.06	-0.02119	-55.6
**TS$_2$$^{\#}$**	-1605.06	-0.01915	-50.3
**I$_2$$^{\#}$**	-1605.11	-0.06835	-179.4
**TS$_3$$^{\#}$**	-1605.03	0.010766	28.3
**I$_3$$^{\#}$**	-1605.05	-0.01177	-30.9
**TS$_4$$^{\#}$**	-1605.03	0.007828	20.6
**I$_4$$^{\#}$**	-1605.05	-0.00859	-22.6
**TS$_5$$^{\#}$**	-1605.05	-0.00603	-15.8
**I$_5$$^{\#}$**	-1605.05	-0.00596	-15.6
**TS$_6$$^{\#}$**	-1605.03	0.014065	36.9
**3$^{\#}$**	-1605.14	-0.0965	-253.3

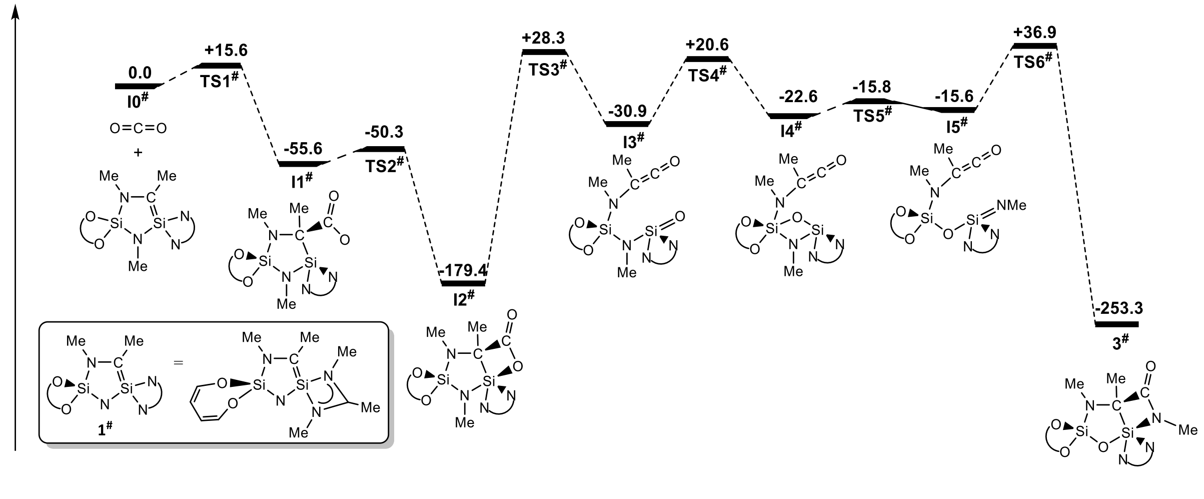

Figure S36. Computed data the reaction of **1**$^{\#}$ with CO_2.

Table S6: Computed data for the full molecule of **3**.

full molecule	G [a.u.]	ΔG [a.u.]	ΔG [kJ/mol]
	-188.252		
	-2883.36		
**I_0**	-3071.61	0	0.0
**I_1**	-3071.61	0.003982	10.5
**I_2**	-3071.64	-0.02462	-64.7
**I_3**	-3071.6	0.013009	34.2
**I_4**	-3071.61	-0.00028	-0.7
**I_5**	-3071.61	-0.00282	-7.4
**3^M**	-3071.67	-0.0563	-147.8

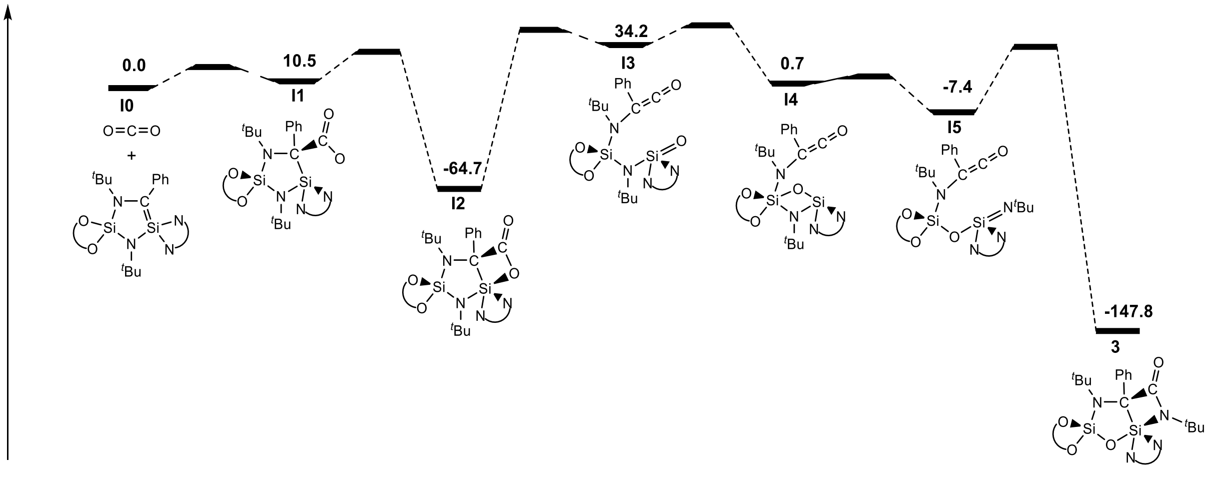

Figure S37. Computed data the reaction of **1** with CO_2.

VI.4. HCl reaction

Table S7: Computed data for **4** and **5**.

	E [a.u.]	$\delta(^1H)$	$\delta(^{29}Si)$	$\delta(^1H$, phenyl)	$\delta(^{13}C)$
**4**_N_on	-3343.922590	4.35	-45, -70	7.14, 7.04, 7.00, 7.38, 8.45	170.9, 56.2
**4**_N_off	-3343.931133	4.38	-15, -46	7.46, 7.15, 7.18, 7.59, 8.91	161.2, 53.9
**5**	-3804.467741	8.19, 15.67	-9, -45	9.11, 7.21, 7.11, 7.49, 8.35	171.1, 49.4

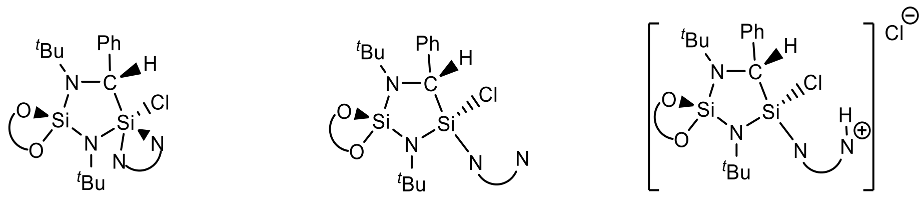

4_N_on **4_N_off** **5**

Figure S38. Computed data the reaction of **4** and **5**.

VI.5. Optimized Structures

VI.5.1. Compound 1

```
0 1
 Si                -0.78841900   -0.34164100    0.64088800
 Si                 1.88753900   -0.20299500    0.43856500
 O                 -2.07597000   -1.06859400    1.44712300
 O                 -1.40029700   -0.07107700   -0.90694500
 N                  3.60786000   -0.80223800    0.86002800
 N                  2.94323800   -0.15460500   -1.08280100
 N                 -0.27626000    1.06852700    1.45828700
 C                 -3.17605900   -1.63764900    0.89913000
 N                  0.61802900   -1.38203400    0.48284600
 C                 -3.52718800   -2.92124100    1.38857800
 H                 -2.92651800   -3.33943500    2.19762200
 C                 -4.49127300    2.71553900   -0.88130900
 C                 -3.94770300   -1.00425500   -0.06965200
 C                 -2.45392300    0.79092800   -0.95044500
 C                  1.42119900    2.58576500    0.38014500
 C                 -5.34112700   -3.05800600   -0.20626400
 C                 -0.60879000    1.43542600    2.86418300
 C                 -5.00792700   -1.75355000   -0.68838000
 C                 -5.51250200    3.69507700   -0.78520500
 H                 -5.31790900    4.69158800   -1.19125200
 C                 -3.69681300    0.40413800   -0.47018200
 C                 -2.22810100    2.08530700   -1.47428600
 H                 -1.23406800    2.31343700   -1.86110800
 C                 -4.72632400    1.40131600   -0.36750000
 C                  6.26441700    0.39994800   -0.87576300
 H                  5.91115300    1.36923900   -0.51694300
 C                 -6.71569700    3.40914200   -0.18379900
 H                 -7.49199300    4.17460000   -0.11118800
 C                  0.46239200    3.59053800    0.10440000
 H                 -0.58269900    3.38227500    0.34177700
 C                 -3.23076000    3.01794700   -1.45957800
 H                 -3.05701500    4.01919000   -1.86259900
 C                 -6.39955200   -3.78171700   -0.81294200
 H                 -6.64448700   -4.77285400   -0.42112300
 C                  4.00820100   -0.55341300   -0.39107200
 C                  5.39208100   -0.69287400   -0.89954400
 C                  2.74945300    0.12108800   -2.51761700
 C                 -5.97266000    1.14698700    0.26492700
 H                 -6.15712500    0.16125500    0.69435100
 C                  7.57378400    0.24986800   -1.32541400
 H                  8.25363500    1.10461000   -1.30588900
 C                 -4.59135600   -3.60526400    0.86527100
 H                 -4.85987600   -4.59056400    1.25511100
 C                  4.36072700   -0.91224600    2.11656300
 C                  2.76802000    2.90942200    0.08553400
 H                  3.55438100    2.20046300    0.36041800
 C                  0.59071800   -2.85118600    0.31925200
 C                 -5.73529400   -1.25514400   -1.80335100
 H                 -5.47416300   -0.27807900   -2.21159100
 C                 -6.93852700    2.12348300    0.35597000
 H                 -7.88466500    1.90145400    0.85572400
 C                 -2.08726800    1.82568300    2.90530700
 H                 -2.27721700    2.66782700    2.22282300
 H                 -2.38339800    2.12514400    3.92299200
 H                 -2.72919100    0.98894900    2.59606200
 C                  3.12202100    4.11999900   -0.50310500
 H                  4.17634000    4.32647300   -0.71305700
 C                  8.01307500   -0.98463600   -1.80434700
 H                  9.04041900   -1.09896900   -2.15793600
 C                  0.21225600   -3.50041100    1.65406700
 H                  0.96255300   -3.26028400    2.42174000
 H                  0.14564000   -4.59572000    1.55975400
 H                 -0.75515200   -3.11991300    2.00532600
 C                 -0.33955500    0.25713000    3.80820900
 H                 -0.97360000   -0.60633600    3.55976400
```

H	-0.53330100	0.53204600	4.85704400
H	0.71456300	-0.05001000	3.72953800
C	1.04548800	1.28716800	0.89881900
C	0.82240800	4.80126800	-0.47465100
H	0.04555600	5.54379200	-0.68154700
C	5.56471500	-1.84751700	1.99257900
H	5.26560000	-2.82050800	1.57419500
H	5.98754100	-2.02182100	2.99320100
H	6.36027400	-1.42869500	1.36274700
C	2.15386600	5.08071300	-0.79896300
H	2.43230900	6.03547400	-1.25054300
C	5.83127300	-1.93024900	-1.37925700
H	5.14831400	-2.78303000	-1.38415800
C	0.24153400	2.61962600	3.31789500
H	1.31351300	2.38229400	3.25270200
H	-0.00021700	2.86082600	4.36410000
H	0.05897600	3.51211500	2.70522900
C	-7.09647500	-3.25918700	-1.87654000
H	-7.90730100	-3.82740600	-2.33858600
C	7.14029400	-2.07204600	-1.83390600
H	7.48155800	-3.03956200	-2.20900200
C	-6.74767500	-1.98691300	-2.38082300
H	-7.28332800	-1.57929500	-3.24171200
C	-0.42808900	-3.22344200	-0.76309700
H	-1.44797000	-2.91422900	-0.49373400
H	-0.44588100	-4.31280700	-0.92032300
H	-0.17432300	-2.73334700	-1.71481100
C	1.96072100	-3.37644900	-0.11199300
H	2.27746800	-2.92135300	-1.06170000
H	1.91406000	-4.46582500	-0.25743200
H	2.72876100	-3.16642200	0.64458900
C	3.79938500	1.10134700	-3.04592000
H	3.81829200	2.00990800	-2.42623800
H	3.53135900	1.39499300	-4.07159200
H	4.80498900	0.66131500	-3.07858300
C	1.36533000	0.75358400	-2.66008800
H	0.57338000	0.09368800	-2.27828400
H	1.16242500	0.95262400	-3.72263600
H	1.31353400	1.70481700	-2.11040500
C	4.80528300	0.48545800	2.56136000
H	5.50444100	0.92427300	1.83411700
H	5.31617000	0.43692500	3.53506900
H	3.93444500	1.15241200	2.65327100
C	2.79881200	-1.18907300	-3.30843700
H	3.77358200	-1.68765300	-3.20083600
H	2.63995400	-0.98728800	-4.37831100
H	2.00811600	-1.87684100	-2.97392800
C	3.38842300	-1.48727600	3.14637300
H	2.48532500	-0.86338000	3.22045100
H	3.86138400	-1.52039900	4.13822500
H	3.08232500	-2.50815100	2.87603600

VI.5.2. Compound 2’

0 1

S	3.85231800	2.43908400	-2.04776400
Si	0.41695400	-0.95779600	-1.04522000
Si	2.40055800	1.40577600	-1.14225100
O	-0.95725400	-1.04670700	-2.01006100
O	-0.09509700	-0.06428400	0.29567000
N	1.72044100	-0.09641800	-1.79450000
N	1.10981400	2.48698700	-0.36587900
N	-0.41167300	-3.41791600	-0.59069000
N	2.54884300	1.29672800	0.69761300
C	-3.65201300	1.25461800	-1.02193100
C	-2.68566600	0.19599000	-0.91360700
C	-1.96647900	-0.15794400	-2.05035600
C	-2.45329300	-0.48207500	0.38743400
C	-2.03880700	-1.52414900	2.99542600
H	-1.88464200	-1.89069500	4.01358800

C	-0.99379700	-0.99994300	2.28063900
H	0.01040600	-0.93109500	2.70194800
C	1.24479600	2.93748800	2.09459200
C	-1.19058800	-0.51468700	0.96475100
C	-3.94593800	1.82320400	-2.30087600
C	-3.53055100	-1.12714300	1.08592400
C	-3.32301900	-1.63826400	2.40605600
C	-2.29191200	0.38087400	-3.32103900
H	-1.72977500	0.01837000	-4.18352700
C	-5.82193300	-1.95119000	1.17380200
H	-6.79004500	-2.09451800	0.68782200
C	1.97133400	-3.13320400	1.64719200
H	1.08333400	-2.82257000	2.20099000
C	-4.30497400	1.80720700	0.11319800
H	-4.07202800	1.41362100	1.10339600
C	0.66294400	-2.75178700	-0.44695200
C	-3.26737300	1.33276500	-3.44593700
H	-3.51050700	1.74869500	-4.42701800
C	1.89068200	4.11430000	2.48489100
H	2.68647500	4.52828300	1.86208300
C	1.92344000	-3.17057100	0.24751200
C	-4.80747900	-1.31804400	0.49277100
H	-4.97416100	-0.96698800	-0.52641300
C	0.21879300	2.40949200	2.88474200
H	-0.29355300	1.50080000	2.56744800
C	2.20879500	-0.61136400	-3.13841400
C	3.71518900	-0.88044500	-3.08315500
H	4.27751000	0.04568500	-2.90449600
H	4.05334800	-1.31431700	-4.03714700
H	3.94662900	-1.59076400	-2.27567400
C	-0.13733400	3.13347900	-2.29575100
H	-0.63368500	2.15478500	-2.32513900
H	0.77682400	3.08861300	-2.90785400
H	-0.81797900	3.87206000	-2.74287300
C	1.62238200	2.25024900	0.83563500
C	4.34459400	-0.27265100	0.83753200
H	4.78480000	0.12951700	-0.08838100
H	3.66283400	-1.09284500	0.57577500
H	5.15463000	-0.68586500	1.45524800
C	-5.62225200	-2.42311300	2.49019700
H	-6.43766000	-2.91843100	3.02252400
C	1.52192000	-1.93086700	-3.51730200
H	1.73105900	-2.73674100	-2.80002400
H	1.91729900	-2.26064500	-4.48878700
H	0.43283700	-1.83417900	-3.61804400
C	0.19982200	3.52734700	-0.85877100
C	-4.88825100	2.87865600	-2.39939200
H	-5.10278700	3.29418900	-3.38781900
C	3.60971900	0.83155400	1.59774200
C	-5.20353400	2.84200800	-0.01224600
H	-5.67984800	3.25315000	0.88119300
C	0.49475200	4.22507100	4.45461800
H	0.20113800	4.72970400	5.37821300
C	-5.50996800	3.38155300	-1.28077600
H	-6.22887100	4.19946000	-1.36865900
C	3.06637500	-3.57775300	-0.45110700
H	3.05036500	-3.62401900	-1.54107100
C	-0.15232400	3.05319400	4.06201400
H	-0.95648900	2.63644800	4.67291500
C	-1.08231400	3.58804700	-0.02909500
H	-0.90653800	3.99721500	0.97487600
H	-1.52441400	2.58743400	0.06908400
H	-1.81867800	4.23289800	-0.53158900
C	3.12750100	-3.50242700	2.33184000
H	3.14487600	-3.46699200	3.42393400
C	-0.71159100	-4.79173600	-0.19710300
C	1.87090100	0.41011200	-4.22717600
H	0.78576400	0.59456200	-4.24945500
H	2.17971900	0.03107700	-5.21391500
H	2.39110100	1.36130500	-4.04644000
C	1.51433500	4.75436400	3.66450000

H	2.02148200	5.67391100	3.96564300
C	4.22112100	-3.95127500	0.23291700
H	5.10080300	-4.27292500	-0.32969800
C	4.59171800	1.97026400	1.89440800
H	4.12942800	2.74467700	2.52244900
H	4.92633300	2.42654700	0.95015300
H	5.46953400	1.58260700	2.43378500
C	-4.39427100	-2.26982100	3.08878500
H	-4.21831200	-2.64681100	4.09999200
C	4.25712100	-3.91333500	1.62638700
H	5.16520300	-4.20217100	2.16076400
C	0.92106700	4.87949300	-0.86253300
H	0.29694200	5.63985000	-1.35664300
H	1.87712900	4.79246400	-1.40081300
H	1.12092200	5.22430400	0.16221400
C	-1.67768700	-5.29807600	-1.27567100
H	-1.17247500	-5.34304000	-2.25286300
H	-2.04841500	-6.30513900	-1.02991100
C	3.02073000	0.26574500	2.88983700
H	3.82315300	-0.16620900	3.50670500
H	2.30144200	-0.53253000	2.65864300
H	2.51508200	1.03984300	3.48357600
C	0.47082700	-5.76075500	-0.09073400
H	0.08631900	-6.78488300	0.03451100
H	1.12786800	-5.54389200	0.76087500
H	1.08035200	-5.74083200	-1.00684600
C	-1.45358700	-4.69869700	1.14145700
H	-2.28738500	-3.98453000	1.07496900
H	-0.77830100	-4.36152200	1.94170000
H	-1.85487000	-5.68552000	1.41882200
H	-2.53049400	-4.61076300	-1.36786200

VI.5.3. Compound 2

0 1

S	4.35993700	-1.30104400	-2.27109600
Si	-0.34547300	-1.09297300	-0.82171600
Si	2.71313700	-0.91022500	-1.21089600
O	-1.46519600	-0.22883600	-1.74948800
O	0.04802500	0.00333800	0.39994600
N	1.10644300	-1.52272100	-1.65267700
N	2.65524900	0.81959500	-0.54830100
N	-0.74953700	-3.15515700	0.80198500
N	2.89701700	-0.96323900	0.62462000
C	-1.43676100	3.39316500	-1.10774100
C	-1.57436700	1.98669900	-0.85679700
C	-1.45877100	1.10836900	-1.93008900
C	-1.84007800	1.47084200	0.51026100
C	-2.27200000	0.51223500	3.14803300
H	-2.42813200	0.15505500	4.16915200
C	-1.20702900	0.06139000	2.41248200
H	-0.50046200	-0.66800300	2.81063000
C	3.22354600	1.19951700	1.86521900
C	-1.00079100	0.52636800	1.09018500
C	-1.30272500	3.86543400	-2.45091500
C	-3.00675400	1.88134800	1.24314800
C	-3.21076000	1.41113100	2.57947700
C	-1.36747300	1.58424900	-3.26119600
H	-1.33600400	0.84504300	-4.06347500
C	-5.13160000	3.06655500	1.38271600
H	-5.89268800	3.69285300	0.91105800
C	-3.38165100	-3.51438000	-1.22311400
H	-2.71677100	-4.27664400	-1.63337300
C	-1.37936200	4.34926400	-0.05779000
H	-1.44903200	4.00644400	0.97562900
C	-1.41546800	-2.45489800	-0.03075400
C	-1.30788300	2.92854600	-3.51593200
H	-1.23080200	3.29218100	-4.54371300
C	4.53930300	1.53602700	2.19559600
H	5.36307900	1.17989200	1.57329900

C	-2.87199100	-2.52419900	-0.37431800
C	-4.01371200	2.70263300	0.66743400
H	-3.89844800	3.03654300	-0.36419800
C	2.16497400	1.65926100	2.65549900
H	1.14038000	1.40063800	2.38664700
C	0.96052900	-2.41406100	-2.87111900
C	1.85506000	-3.65055400	-2.74557600
H	2.91825500	-3.37869200	-2.71552300
H	1.69018300	-4.31523600	-3.60796400
H	1.60171400	-4.20522900	-1.82880500
C	2.19072500	2.04237900	-2.54449400
H	1.12839000	1.77320100	-2.47870300
H	2.71645300	1.28011200	-3.14068100
H	2.26577800	3.00564600	-3.06933200
C	2.92956100	0.36806300	0.67323500
C	2.88987800	-3.32936600	0.92912800
H	3.46316600	-3.45833800	-0.00239800
H	1.81440200	-3.36175200	0.69787900
H	3.13433600	-4.16666000	1.59974100
C	-5.30765200	2.63396800	2.71592300
H	-6.19600800	2.93589500	3.27560900
C	-0.47512200	-2.93376000	-2.99539200
H	-0.71239500	-3.62489100	-2.17474700
H	-0.56520700	-3.50334500	-3.93180700
H	-1.23193900	-2.13564400	-3.03305000
C	2.80867700	2.14926500	-1.15131800
C	-1.14567600	5.25470000	-2.69089700
H	-1.04877500	5.59692300	-3.72485200
C	3.25258500	-2.00060700	1.59525100
C	-1.21338600	5.68937100	-0.32184400
H	-1.16050600	6.39998300	0.50657900
C	3.73805500	2.78288700	4.10453100
H	3.93995600	3.40324600	4.98105700
C	-1.10232300	6.15358700	-1.65131000
H	-0.97400400	7.22035200	-1.84889500
C	-3.74564300	-1.54814400	0.12168100
H	-3.36357000	-0.76159400	0.77299000
C	2.42503800	2.44689400	3.77326100
H	1.59384800	2.80247300	4.38664400
C	2.07241700	3.21724500	-0.34316500
H	2.57220100	3.43271800	0.61085500
H	1.04156100	2.90012800	-0.13752600
H	2.02828900	4.15348900	-0.91954700
C	-4.73739100	-3.54014100	-1.54833800
H	-5.11858400	-4.32155300	-2.21048100
C	-1.25362600	-4.24325700	1.64977000
C	1.29413600	-1.60663600	-4.12705900
H	0.62685400	-0.73421500	-4.20467700
H	1.16391800	-2.22654100	-5.02790900
H	2.33565800	-1.25637100	-4.09750000
C	4.79283500	2.32812700	3.31432000
H	5.82234000	2.59108000	3.56816400
C	-5.09981100	-1.57933300	-0.19579100
H	-5.76300600	-0.81225100	0.21183300
C	4.75933700	-1.96462300	1.86977500
H	5.04380200	-1.05627900	2.41971100
H	5.31175900	-1.99290200	0.91820200
H	5.05563400	-2.83231200	2.47897300
C	-4.36551900	1.81848500	3.29629200
H	-4.49823500	1.45704000	4.31962700
C	-5.60182100	-2.57632500	-1.03261600
H	-6.66419400	-2.59832500	-1.28742300
C	4.29758100	2.48798400	-1.28110900
H	4.42512200	3.42700500	-1.84149700
H	4.82229300	1.67768300	-1.80964800
H	4.75852600	2.62303000	-0.29165200
C	-0.33892800	-4.26711400	2.87889800
H	-0.44614600	-3.33690400	3.45778700
H	-0.59598300	-5.11029500	3.53780100
C	2.45044800	-1.83695500	2.88567400
H	2.65783100	-2.67800100	3.56427800

H	1.37633500	-1.83703200	2.65467900
H	2.70507300	-0.90689400	3.41237300
C	-2.70305000	-4.10865600	2.13140200
H	-2.88993700	-4.85665700	2.91735000
H	-3.43995600	-4.26768800	1.33539000
H	-2.87845000	-3.11142900	2.56291300
C	-1.06227000	-5.54120500	0.85689500
H	-0.02556100	-5.62679500	0.49740700
H	-1.73579200	-5.57369500	-0.01155100
H	-1.28249300	-6.41104000	1.49464400
H	0.71307900	-4.35999500	2.57862400

VI.5.4. Compound 3

0 1

Si	1.98637100	-0.21013600	-0.34123600
Si	-0.84376300	-0.68726700	-0.39885000
O	0.38606100	0.37926900	-0.23073500
O	-1.92909600	-0.22418800	-1.58824000
O	-1.72111500	-0.57089000	1.02763300
O	2.52678700	-3.23812100	-2.25764800
N	2.94670600	0.48721800	1.08088700
N	2.98678900	-1.06551300	-1.58239600
N	2.48138600	1.64837200	-0.68638800
N	-0.06558100	-2.15477500	-0.76964700
C	-3.04063000	-0.85749500	1.13330400
C	0.92952200	-3.98215200	2.93024100
H	0.21456500	-3.99091900	3.75739900
C	-5.35738100	-0.61234900	0.45703700
C	2.97097600	1.71879700	0.53193700
C	0.72378400	-3.12528800	1.85363000
H	-0.15374800	-2.47449500	1.83877200
C	4.15746100	-0.87684600	-2.46967000
C	-3.40731100	-1.89107800	2.02891000
H	-2.61450800	-2.36961900	2.60713000
C	1.62233300	-3.08176300	0.77503300
C	-5.72124600	-1.66728600	1.35202100
C	-3.99455500	-0.15982100	0.40267700
C	-2.65138400	0.91829600	-1.41251400
C	-3.61437900	1.01935000	-0.41697300
C	2.72422600	-3.94682700	0.80754400
H	3.41254200	-3.97838300	-0.03918300
C	1.88480400	2.71502800	-1.51829700
C	4.55344400	-1.21114800	1.72572200
H	5.03920700	-1.73020200	2.56531800
H	5.33661300	-0.82065300	1.05808300
H	3.96630300	-1.95455300	1.17633400
C	-4.71692800	-2.27228200	2.15049000
H	-4.99831300	-3.06760200	2.84551100
C	-0.06397300	-4.64583100	-1.14073400
H	-0.13140400	-4.89843400	-0.07395500
H	0.98938100	-4.65124400	-1.44669000
H	-0.58762600	-5.42967000	-1.70922000
C	0.72154000	3.40252600	-0.79308000
H	1.07619200	3.96473100	0.08220300
H	-0.02117400	2.66423600	-0.46715300
H	0.23053500	4.11726400	-1.47072200
C	3.67507500	-0.06902700	2.24066900
C	-0.70735000	-3.06151200	-2.96601900
H	-1.19407700	-2.10446900	-3.20666900
H	-1.24217500	-3.86456700	-3.49773100
H	0.32801500	-3.04126700	-3.33336600
C	2.04207100	-4.82491400	2.95791600
H	2.20372900	-5.50141200	3.80061800
C	2.33725700	-2.27641800	-1.53604100
C	-0.73016100	-3.30531200	-1.45159700
C	2.93514200	-4.80211000	1.88944500
H	3.80064000	-5.46982400	1.88471800
C	-2.38520600	1.99028000	-2.29697800
H	-1.65536100	1.82484100	-3.09045700

C	1.35036700	-2.13705700	-0.37007200
C	-3.03182600	3.18861200	-2.14943900
H	-2.83162100	4.01295800	-2.83892200
C	-6.37223600	-0.07041000	-0.37707000
H	-6.10942700	0.70944400	-1.09300000
C	1.36072500	2.03177900	-2.78346100
H	0.87692100	2.77303700	-3.43589900
H	0.62097300	1.26016900	-2.53418100
H	2.17713500	1.56366600	-3.35034700
C	-3.94518000	3.38742700	-1.08283300
C	-7.07070100	-2.10009000	1.41195200
H	-7.33019900	-2.90160400	2.10906200
C	3.38549000	2.96091400	1.22889900
C	-4.22920800	2.29994600	-0.19790900
C	2.64064900	-0.57680300	3.24419700
H	2.02235700	0.25731600	3.60931600
H	3.13627900	-1.04901400	4.10566000
H	1.98009100	-1.32440800	2.78834500
C	-5.67625900	3.78065900	1.08813200
H	-6.33566200	3.94182200	1.94450300
C	3.71948400	-0.96172000	-3.93473300
H	4.58385400	-0.81195700	-4.59999200
H	2.97081000	-0.18945500	-4.16449500
H	3.27940200	-1.94540700	-4.14466700
C	-5.09901900	2.54586100	0.89859400
H	-5.30070500	1.73971900	1.60513200
C	4.82145400	0.46914900	-2.20028100
H	5.10041800	0.56829400	-1.13998600
H	4.16913300	1.30836900	-2.45992200
H	5.73826100	0.54465000	-2.80361600
C	4.59761900	0.92803300	2.94366700
H	5.14097500	0.37879200	3.72649900
H	4.05753600	1.74997300	3.42895500
H	5.34372200	1.35675800	2.26033800
C	-2.18098600	-3.37996700	-0.96557500
H	-2.74757400	-2.46412500	-1.19371700
H	-2.22165100	-3.55831200	0.11910600
H	-2.70245300	-4.20971300	-1.46549800
C	2.91705400	3.77203300	-1.92975500
H	3.23791700	4.38727600	-1.07977200
H	2.46388900	4.44581700	-2.67235700
H	3.80690200	3.31594900	-2.38763300
C	-8.03181000	-1.53824200	0.60517200
H	-9.06705000	-1.88360900	0.65689600
C	2.55965700	3.48631200	2.22800300
H	1.62853200	2.97325800	2.48077000
C	-7.67061100	-0.52126900	-0.30571000
H	-8.42768900	-0.09048900	-0.96558400
C	-5.42092200	4.84109900	0.19082700
H	-5.89129100	5.81479300	0.34724700
C	5.19083800	-1.97314600	-2.16694000
H	5.49094100	-1.93457000	-1.10874000
H	6.08839200	-1.81528500	-2.78395400
H	4.78909200	-2.97004700	-2.38410300
C	-4.56768400	4.64415700	-0.86900400
H	-4.34634400	5.45999000	-1.56269800
C	4.57872800	3.60776700	0.89811400
H	5.22001700	3.19154600	0.11779500
C	2.92637800	4.65657500	2.88798600
H	2.27544000	5.06898400	3.66228900
C	4.12308100	5.29623900	2.56400900
H	4.41301800	6.21037200	3.08750800
C	4.94939000	4.76931200	1.57137700
H	5.88801300	5.26731900	1.31771200

VI.5.5. Compound 4 N off

0 1

Cl	3.01514100	0.56100100	-2.12015000
Si	1.90230600	0.05679300	-0.41342000

Si	-0.90753700	-0.01216700	-0.07707900
O	-1.78948100	-1.00732100	0.94466800
O	-2.06051400	0.42981000	-1.22982000
N	0.41977200	-0.71475700	-0.93509600
N	2.96643400	-0.95410100	0.57333100
N	4.43250400	0.80942000	0.65276400
N	-0.16228400	1.21397600	0.87021400
C	-4.02462700	-1.17311700	0.06484300
C	-2.83806300	-3.11085200	0.97169700
H	-1.94201400	-3.47341800	1.47906700
C	-2.89121500	-1.73384100	0.64346000
C	0.28092900	3.81690000	-2.69304700
H	-0.34762500	3.75417600	-3.58538000
C	4.29295100	-0.45452400	0.58242300
C	-3.87576600	-3.94510300	0.65417900
H	-3.83185200	-5.00585300	0.91425200
C	0.30270500	2.75384400	-1.79334800
H	-0.30946500	1.87456900	-2.00053200
C	6.35586500	-1.76263500	1.29915300
H	6.35436100	-1.24992700	2.26287600
C	1.14563400	1.64014500	0.33422000
H	1.81929900	1.90673300	1.16196800
C	-4.16053200	0.29657500	-0.10026900
C	-6.18892800	-1.60228500	-1.11468000
H	-6.24414200	-0.54887900	-1.39275000
C	1.10062400	2.79848000	-0.64246800
C	-5.25691200	1.01013400	0.49596800
C	1.86778700	5.00406100	-1.32964200
H	2.48582400	5.88435400	-1.13515100
C	-3.19976100	1.02726500	-0.78505500
C	-5.39548600	2.41585700	0.26745400
C	1.88057900	3.93916700	-0.43060100
H	2.50361100	3.99779300	0.46408600
C	-5.07581700	-2.05828300	-0.35825400
C	6.41814800	1.70868100	1.75561700
H	6.88871200	0.75665800	2.03491500
H	5.73921600	2.01436800	2.56638600
H	7.21381300	2.46453500	1.66767000
C	5.36570700	-1.47448100	0.35518100
C	0.02263200	-0.90060800	-3.36907700
H	0.86398400	-0.22632200	-3.57913200
H	-0.89684800	-0.30259100	-3.29353600
H	-0.08635300	-1.59595200	-4.21622800
C	-5.00589600	-3.45231900	-0.04749000
C	5.16382600	2.99038800	0.03567200
H	6.01263800	3.66477100	-0.15526700
H	4.55488700	3.41896700	0.84434100
H	4.54082700	2.93773900	-0.86862000
C	1.50025200	-2.55725600	-2.20005200
H	2.39303600	-1.96954300	-2.44998300
H	1.34869300	-3.28984800	-3.00655800
H	1.69220800	-3.10885600	-1.26943500
C	0.25748300	-1.67537600	-2.06779900
C	7.33831900	-3.38590600	-0.19676400
H	8.10809800	-4.13100700	-0.41197000
C	-6.48180500	3.11619400	0.85243100
H	-6.57661400	4.18767300	0.65617300
C	5.36528300	-2.16286000	-0.86408200
H	4.58553700	-1.94112300	-1.59530600
C	6.56849300	1.07664400	-0.68435100
H	7.32192100	1.84400800	-0.91907800
H	5.98363500	0.88154500	-1.59542400
H	7.10351300	0.15819600	-0.41359800
C	-7.18451000	-2.46611100	-1.51079200
H	-8.02416500	-2.08756400	-2.09882300
C	6.35258500	-3.10227600	-1.14349100
H	6.35031200	-3.62227400	-2.10459100
C	-0.59931700	3.42845800	1.89928900
H	0.41892000	3.81557800	1.76614600
H	-1.03168100	3.92743300	2.77982400
H	-1.18736300	3.71122600	1.01390200

C	-0.62808400	1.90944600	2.10189300
C	-2.06152300	1.52340000	2.46669200
H	-2.76945500	1.80079800	1.67405900
H	-2.35399100	2.07285700	3.37328000
H	-2.16407100	0.45112100	2.66989600
C	-0.93876400	-2.59229300	-1.81079200
H	-1.04350600	-3.31120000	-2.63677200
H	-1.88120400	-2.02883700	-1.75242400
H	-0.81411600	-3.16470300	-0.88043500
C	0.27891400	1.52537000	3.27854400
H	0.19852500	0.44960700	3.49423700
H	-0.01522900	2.07708600	4.18424900
H	1.33636400	1.75948500	3.08255900
C	2.60527100	-1.95895800	1.62665700
C	1.09160000	-2.09043500	1.75612800
H	0.61368800	-1.11965000	1.94367800
H	0.86327800	-2.74042700	2.61335400
H	0.63441600	-2.53114200	0.86308100
C	3.13835900	-1.45641100	2.97390200
H	2.85337200	-2.15023700	3.77921800
H	2.72611500	-0.46298900	3.20385200
H	4.23403500	-1.37789000	2.97819500
C	3.16361200	-3.35780100	1.32804700
H	2.88302200	-3.68122800	0.31608500
H	2.74149100	-4.07695500	2.04689100
H	4.25535800	-3.41085700	1.40808800
C	-6.05505900	-4.31454100	-0.45664000
H	-5.98761000	-5.37543900	-0.20040000
C	-6.19713500	0.38251200	1.35720100
H	-6.08654900	-0.67935800	1.57943700
C	-3.33974100	2.41696500	-1.01441200
H	-2.55472600	2.92620100	-1.57596400
C	5.65222500	1.59371200	0.43293900
C	1.06427600	4.94855000	-2.46665600
H	1.04897300	5.78161400	-3.17372600
C	-4.42439400	3.09001000	-0.51694700
H	-4.53603600	4.16282200	-0.69388400
C	7.33354100	-2.71857500	1.02726800
H	8.09578000	-2.94298900	1.77736600
C	-7.38826000	2.47129100	1.65990700
H	-8.21795900	3.02258400	2.10861400
C	-7.23140000	1.09262300	1.92277600
H	-7.93492200	0.58360700	2.58614600
C	-7.12814000	-3.83552200	-1.17052100
H	-7.92794200	-4.51033000	-1.48452900

VI.5.6. Compound 4 N on

0 1

Si	-0.84259400	-0.03377100	0.15736100
Si	1.87496900	0.11827700	-0.45483300
O	-1.89247300	-0.82030000	1.21683500
O	-1.86133400	0.36331500	-1.12102900
N	2.98582900	-0.99637200	0.76104600
N	3.71112500	0.12718400	-0.93388600
N	-0.13324300	1.28646500	1.03127900
C	-2.96776400	-1.57028100	0.87557400
N	0.46206600	-0.96349100	-0.47759300
C	-3.00075000	-2.88509000	1.40529000
H	-2.19255800	-3.17403400	2.07990500
C	-5.38157900	2.46200300	-0.48096600
C	-4.00612200	-1.08701600	0.08461000
C	-3.04812900	0.97995300	-0.94822800
C	1.79117000	2.82174800	0.43054900
C	-5.02279900	-3.35563600	0.16582700
C	-0.74487800	1.91035200	2.25300500
C	-5.01177700	-2.02123800	-0.34848000
C	-6.54099400	3.22162100	-0.18033200
H	-6.61203400	4.23984000	-0.57277900
C	-4.10061000	0.34450900	-0.30327700

C	-3.16977100	2.29807900	-1.45149100
H	-2.31747300	2.70539200	-1.99904900
C	-5.26977900	1.12132100	0.00728100
C	6.34620600	-0.77511300	1.13412400
H	6.12956600	0.15924200	1.65634800
C	-7.54840200	2.70197900	0.59798900
H	-8.43386800	3.29969100	0.82695700
C	1.07019100	3.63472800	-0.45029300
H	0.11438400	3.27241900	-0.83241300
C	-4.31782500	3.01403800	-1.24161300
H	-4.41641600	4.03030500	-1.63213400
C	-6.03031800	-4.26136200	-0.25437700
H	-6.02661400	-5.27332900	0.16001200
C	4.05871300	-0.71184500	0.05880900
C	5.39724900	-1.32490300	0.27024900
C	4.55037900	0.71346100	-2.01981700
C	-6.31932400	0.62598900	0.82740700
H	-6.23748100	-0.37895100	1.24339700
C	7.57744500	-1.40372000	1.30761100
H	8.31793700	-0.96655000	1.98135500
C	-4.01198100	-3.74842700	1.07937700
H	-4.03493500	-4.75753400	1.49884100
C	2.86994400	-1.72493200	2.03631200
C	3.00460600	3.29913800	0.93703200
H	3.56993900	2.67975000	1.64148900
C	0.34481300	-2.14014500	-1.38988900
C	-5.99887200	-1.67547400	-1.31085500
H	-5.98626300	-0.67520400	-1.74547000
C	-7.42435200	1.39430500	1.11591800
H	-8.21086200	0.98766100	1.75648200
C	-2.23385600	2.17044400	2.01775300
H	-2.38356400	2.81137300	1.13720600
H	-2.66074900	2.68526100	2.89125000
H	-2.80416700	1.24552600	1.87506200
C	3.49305400	4.55311000	0.57361000
H	4.43946400	4.91046300	0.98798300
C	7.86652200	-2.58167200	0.61983900
H	8.83309400	-3.07205900	0.75705000
C	-0.14405900	-3.36480900	-0.61345200
H	0.56977400	-3.65654400	0.17070400
H	-0.26577400	-4.22091700	-1.29502300
H	-1.11850600	-3.18013200	-0.14347300
C	-0.57149300	0.99617600	3.47304700
H	-1.04283600	0.01920200	3.30289900
H	-1.03300000	1.45335500	4.36196700
H	0.49451800	0.84252900	3.70336700
C	1.30892800	1.44685800	0.82677300
C	1.55655900	4.88551700	-0.81993200
H	0.98059700	5.50622500	-1.51116000
C	3.43152200	-3.14911600	1.94574700
H	2.99149100	-3.68511600	1.09114000
H	3.17044600	-3.70127800	2.86114800
H	4.52412700	-3.16925700	1.84562300
C	2.77106200	5.35070900	-0.31232600
H	3.14797000	6.33513800	-0.60039000
C	5.68918300	-2.50586200	-0.42095900
H	4.94490200	-2.93163400	-1.09828800
C	-0.11266900	3.26383600	2.59484400
H	0.95813800	3.18965100	2.82638800
H	-0.61553100	3.65805000	3.49065900
H	-0.23469200	3.99466100	1.78559000
C	-6.98308800	-3.88567900	-1.17155800
H	-7.75108200	-4.59372600	-1.49189900
C	6.92055300	-3.13110700	-0.24596600
H	7.14250500	-4.05267600	-0.78889200
C	-6.95392900	-2.58143800	-1.71227100
H	-7.69358100	-2.28803800	-2.46125100
C	-0.61469300	-1.88748700	-2.55919400
H	-1.65267200	-1.78881700	-2.21340100
H	-0.57079100	-2.73305100	-3.26340400
H	-0.34635300	-0.96375600	-3.08774800

C	1.72878400	-2.46121200	-1.95877100
H	2.10841400	-1.62815900	-2.56759700
H	1.67859700	-3.34698900	-2.60854400
H	2.44214300	-2.67696500	-1.14877700
C	6.05775600	0.59955000	-1.75953500
H	6.34407300	1.04976200	-0.79934700
H	6.56578200	1.16752300	-2.55227400
H	6.43974900	-0.42727100	-1.79703800
C	4.26956300	2.21720600	-2.12524600
H	3.20993600	2.43892800	-2.28236800
H	4.83717200	2.62905500	-2.97330700
H	4.59601600	2.73178200	-1.21148500
C	3.58339200	-0.95023000	3.15293200
H	4.66867700	-0.91291600	2.99458700
H	3.40559000	-1.43822600	4.12312100
H	3.20039800	0.07988700	3.21741600
C	4.24745200	-0.00505700	-3.33897800
H	4.47102000	-1.07992200	-3.25126600
H	4.87579000	0.40778600	-4.14275300
H	3.19643800	0.11979500	-3.62673700
C	1.39060700	-1.82061500	2.40221600
H	0.92380800	-0.83191000	2.44431700
H	1.29126500	-2.28371900	3.39486100
H	0.82934500	-2.41431600	1.67548600
H	1.85252700	1.17064100	1.75004200
Cl	1.23517200	1.09855300	-2.33868600

VI.5.7. S_Int 1 (2A)

0 1

Si	-0.81099100	-0.25776900	0.57563600
Si	2.00371600	-0.20501100	0.65935400
O	-2.08128900	-0.94985600	1.43208400
O	-1.48040300	-0.01571400	-0.95990700
N	3.66416400	-1.24624300	0.69992100
N	3.02923200	0.11095700	-0.86107900
N	-0.34836800	1.18223600	1.39545100
C	-3.16289600	-1.60998400	0.95879700
N	0.60663800	-1.23848800	0.35618000
C	-3.44899200	-2.85656100	1.57079700
H	-2.82516800	-3.16288800	2.41189700
C	-4.80278300	2.47492000	-1.18574600
C	-3.97457200	-1.10181500	-0.05016500
C	-2.60746200	0.72705000	-1.07970300
C	1.41950700	2.74689200	0.58953400
C	-5.25396600	-3.23328300	0.00561100
C	-1.07314000	1.83853800	2.53065600
C	-4.99230800	-1.96201700	-0.59443000
C	-5.89833800	3.37547300	-1.16613700
H	-5.78854000	4.34046800	-1.66855900
C	-3.81942500	0.28147900	-0.56955800
C	-2.50289600	1.97139200	-1.74824900
H	-1.54352400	2.22437400	-2.20208300
C	-4.92485800	1.20044600	-0.54797100
C	6.22849700	0.33437200	-1.04180700
H	5.92519000	1.24078300	-0.51284900
C	-7.06799900	3.05453600	-0.51866200
H	-7.90266700	3.75937400	-0.50459800
C	0.50513800	3.42537200	-0.23455800
H	-0.48225700	2.98716700	-0.38495400
C	-3.57877300	2.81633000	-1.81656300
H	-3.49690700	3.77499700	-2.33543400
C	-6.26972700	-4.06665400	-0.52857400
H	-6.46030700	-5.02950900	-0.04656900
C	4.03633800	-0.65897800	-0.42464500
C	5.36099100	-0.76132800	-1.08382700
C	2.77556500	0.62808600	-2.22754500
C	-6.14068000	0.91089100	0.12782000
H	-6.24155300	-0.03990700	0.65279500
C	7.47362500	0.26093400	-1.66414300

H	8.15180400	1.11625000	-1.62138700
C	-4.47802600	-3.63871400	1.12104200
H	-4.69666800	-4.59485700	1.60347100
C	4.50933400	-1.83029500	1.76192900
C	2.66133200	3.35668900	0.81946500
H	3.37368000	2.86180700	1.48230200
C	0.49605400	-2.68415300	0.00100300
C	-5.74348100	-1.60938700	-1.74839900
H	-5.53623100	-0.66159000	-2.24649600
C	-7.18002200	1.81299500	0.14471300
H	-8.09912600	1.56616100	0.68178200
C	-2.50746100	2.15166300	2.09974400
H	-2.52000600	2.77818600	1.19483300
H	-3.02983700	2.69486000	2.90203400
H	-3.08125900	1.23948700	1.89170600
C	2.98048300	4.58266300	0.23630500
H	3.95410300	5.03596500	0.44138500
C	7.84829400	-0.89754600	-2.34281800
H	8.82127300	-0.95110900	-2.83697400
C	0.22126300	-3.49118600	1.27255200
H	1.03316300	-3.35374800	1.99887500
H	0.12428800	-4.56568300	1.05068500
H	-0.70774500	-3.14982700	1.74688000
C	-1.08598400	0.90866800	3.75332400
H	-1.59144300	-0.03802900	3.52072500
H	-1.62094200	1.38716300	4.58907300
H	-0.05936900	0.68641200	4.07613200
C	1.03952000	1.40907400	1.14188200
C	0.82641200	4.64365500	-0.82436200
H	0.08979700	5.14392800	-1.45907300
C	5.38112300	-2.98095900	1.24347400
H	4.78137500	-3.71272300	0.68129200
H	5.82936200	-3.50275400	2.10237500
H	6.20198100	-2.63340500	0.60447900
C	2.07145000	5.23230600	-0.59669000
H	2.32360900	6.19253300	-1.05295800
C	5.74277600	-1.92612300	-1.75787400
H	5.05879600	-2.77642400	-1.79429600
C	-0.39865800	3.15227800	2.93361800
H	0.65582600	3.00913800	3.20777300
H	-0.92187800	3.55085600	3.81571900
H	-0.44971700	3.90753100	2.13897200
C	-6.99194200	-3.68358500	-1.63393500
H	-7.76900900	-4.33576700	-2.03955500
C	6.97995200	-1.98940500	-2.39123600
H	7.26968700	-2.89715800	-2.92541800
C	-6.71210300	-2.44625000	-2.25405300
H	-7.26637600	-2.14998700	-3.14801600
C	-0.64390400	-2.90307800	-1.00143700
H	-1.62708100	-2.62535700	-0.60044900
H	-0.69806500	-3.96882100	-1.26904900
H	-0.48277700	-2.31793300	-1.91767800
C	1.76876000	-3.20333000	-0.66790200
H	2.01660600	-2.59882400	-1.55332200
H	1.61125600	-4.24080400	-0.99765400
H	2.62686500	-3.18513100	0.01179200
C	3.40687800	2.00942200	-2.40660900
H	3.02054400	2.71962000	-1.66541900
H	3.17466900	2.39695800	-3.41023100
H	4.50101300	1.96050300	-2.31079300
C	1.26136900	0.72953100	-2.39566200
H	0.77588000	-0.25040000	-2.30197100
H	1.02604500	1.14255800	-3.38749500
H	0.82463100	1.39644500	-1.64432300
C	5.40611200	-0.75106600	2.38014200
H	6.10639200	-0.34930900	1.63268700
H	6.00105600	-1.17801300	3.20200900
H	4.80227300	0.07825400	2.77444900
C	3.30372400	-0.31917000	-3.31081100
H	4.39815500	-0.31210600	-3.38617200
H	2.90497800	0.00247500	-4.28417800

H	2.96862600	-1.35323700	-3.13778600
C	3.57354800	-2.40605800	2.82703100
H	2.85738600	-1.65771200	3.19237100
H	4.16477900	-2.76478500	3.68196700
H	3.01079000	-3.26118000	2.42639600
S	2.22127500	0.80128700	2.53100500

VI.5.8. S_Int 1 (2B)

0 1

Si	0.67729300	-0.30511900	-0.89178800
Si	-2.07513700	-0.54465800	-1.23839300
O	2.08436100	-0.79750000	-1.66285900
O	1.16797700	-0.07775300	0.69981000
N	-3.60019500	-0.97941200	-0.48439200
N	-4.99855800	-0.70779000	1.34938900
N	0.08918400	1.11844600	-1.68184500
C	3.16812000	-1.39973400	-1.11066500
N	-0.63647200	-1.45371700	-0.88830000
C	3.62563700	-2.57969800	-1.74838000
H	3.12592800	-2.89123000	-2.66651900
C	4.19851500	2.72927700	1.24761200
C	3.81970700	-0.88692500	0.00476900
C	2.19845500	0.78331600	0.92009100
C	-1.95619400	2.40116000	-1.00649200
C	5.27733100	-2.89815100	-0.00865800
C	0.82387400	1.89884300	-2.73711300
C	4.84481600	-1.68768300	0.61718300
C	5.20428700	3.72397400	1.35177500
H	4.94935900	4.67491800	1.82752400
C	3.49350600	0.45729900	0.54319000
C	1.89919300	2.02464600	1.53016300
H	0.86984400	2.19449800	1.84118000
C	4.50892400	1.47087700	0.64263900
C	-1.85610200	-1.18969900	2.33817000
H	-2.05369300	-2.25019700	2.17318800
C	6.46744200	3.50891500	0.85366800
H	7.23238300	4.28478600	0.93428000
C	-1.17905800	3.22963800	-0.18374800
H	-0.12154900	2.99512000	-0.05922800
C	2.87870000	2.96485900	1.71018500
H	2.64523000	3.92102300	2.18598700
C	6.29881100	-3.67401900	0.59675100
H	6.62254900	-4.59105800	0.09691700
C	-3.82678000	-0.64155700	0.86714200
C	-2.61956700	-0.22915200	1.66202000
C	-5.48186900	-0.58459500	2.71797800
C	5.81747600	1.29303600	0.11796500
H	6.06600900	0.35730800	-0.38397700
C	-0.87349200	-0.80574600	3.24486400
H	-0.28372600	-1.56592100	3.76219200
C	4.66448100	-3.29998000	-1.22255100
H	5.01618300	-4.20581200	-1.72293700
C	-4.74121500	-1.56592200	-1.29164000
C	-3.31270200	2.71351300	-1.16560500
H	-3.92536600	2.09521300	-1.82347400
C	-0.41792400	-2.92465600	-0.81143400
C	5.43781500	-1.33378200	1.85910400
H	5.09879900	-0.43119100	2.36936200
C	6.76750400	2.28379100	0.21893900
H	7.76273100	2.12054900	-0.20168600
C	2.15586400	2.38256700	-2.16139900
H	2.00074700	2.99457900	-1.26031100
H	2.68398600	2.99576000	-2.90719500
H	2.81541500	1.54611000	-1.89592000
C	-3.87959100	3.79579600	-0.49481900
H	-4.94279800	4.01106800	-0.62783100
C	-0.64879600	0.54691200	3.49692700
H	0.11767500	0.84979200	4.21431100
C	0.05512200	-3.42327800	-2.18065200

H	-0.72064900	-3.24063100	-2.94050500
H	0.27236900	-4.50252200	-2.15681000
H	0.95992900	-2.89042600	-2.49807200
C	1.07864100	1.01821500	-3.97026800
H	1.69279400	0.14563600	-3.71367800
H	1.61280800	1.59662300	-4.74027200
H	0.12931700	0.66560600	-4.39598800
C	-1.33632800	1.16619900	-1.58125100
C	-1.74137400	4.32048200	0.47252200
H	-1.11081500	4.95169600	1.10469800
C	-5.30468100	-2.80941100	-0.58463300
H	-4.53018700	-3.58151100	-0.47071500
H	-6.11330900	-3.23041300	-1.20062000
H	-5.70824600	-2.56137000	0.40056700
C	-3.10015000	4.60479900	0.33202700
H	-3.54540000	5.45591900	0.85264200
C	-2.37154300	1.12607500	1.90373300
H	-2.98154200	1.88339600	1.41031800
C	0.02323300	3.12340000	-3.18863700
H	-0.97450000	2.85457300	-3.56297500
H	0.56873600	3.60113400	-4.01578000
H	-0.09633100	3.86622900	-2.39042200
C	6.86379200	-3.29222500	1.79053500
H	7.64679900	-3.89998000	2.25005000
C	-1.39602100	1.50871100	2.82088900
H	-1.23500000	2.57242900	3.01241700
C	6.41643100	-2.11503200	2.42997300
H	6.84818000	-1.82225700	3.39015800
C	0.61637000	-3.23073000	0.27482000
H	1.59968700	-2.79346800	0.05807700
H	0.76039400	-4.31766900	0.36423800
H	0.28403800	-2.83361200	1.24392900
C	-1.70507700	-3.66123800	-0.45154500
H	-2.13060100	-3.30932700	0.49570000
H	-1.49494000	-4.73675400	-0.35779000
H	-2.47136000	-3.54572800	-1.22731700
C	-5.25560900	-1.93103000	3.41921800
H	-5.69826600	-2.75415600	2.83807600
H	-5.71702900	-1.92723400	4.41909400
H	-4.18027000	-2.12964100	3.54003200
C	-6.99057000	-0.33596500	2.58339600
H	-7.17361600	0.62376200	2.07570500
H	-7.48083900	-0.30557600	3.56885600
H	-7.45629000	-1.12972300	1.98076300
C	-5.83031100	-0.51154800	-1.50658400
H	-6.24324100	-0.17635700	-0.54847600
H	-6.64129200	-0.93505500	-2.11941900
H	-5.41152800	0.35087500	-2.04645200
C	-4.88998300	0.53939300	3.57900400
H	-3.85468700	0.34276800	3.88474700
H	-5.49227000	0.64321600	4.49497000
H	-4.91871000	1.50131900	3.04610600
C	-4.27915700	-2.03760900	-2.68019500
H	-4.15141000	-1.21247300	-3.38963100
H	-5.05354400	-2.70416700	-3.08622200
H	-3.34344000	-2.61788300	-2.65513900
S	-2.26323500	0.41598000	-3.10460500

VI.5.9. S_Int 2 (2C-1)

0 1

S	-0.25442500	2.30982400	-1.87958900
Si	-0.46276300	0.10887500	0.30004200
Si	2.25841200	0.13258300	-0.27651100
O	-1.68563800	0.14880700	1.47343900
O	-1.12722100	-0.81932700	-0.93528600
N	1.02909500	-0.61852300	0.79201300
N	3.97153500	0.04146700	0.54185400
N	-1.63578500	2.30214000	-0.76015000
N	3.31953400	-1.23998200	-1.04568400

C	-4.40799000	-2.28649900	0.96255400
C	-3.50833000	-1.20340500	0.65777700
C	-2.68895600	-0.73441500	1.68049400
C	-3.50109300	-0.62282200	-0.71038200
C	-3.55408000	0.18629300	-3.42872400
H	-3.58130000	0.46189000	-4.48617900
C	-2.37592400	-0.18058300	-2.83817700
H	-1.43182500	-0.18596300	-3.38354500
C	5.81602700	-1.09949700	-0.74076000
C	-2.34277500	-0.53899200	-1.46939000
C	-4.57112500	-2.73102200	2.31178200
C	-4.71869000	-0.12025000	-1.28769100
C	-4.74621000	0.27006500	-2.66349400
C	-2.88192900	-1.15475300	3.02154500
H	-2.26471300	-0.69097500	3.79239100
C	-7.05328000	0.56000100	-1.09584300
H	-7.94572200	0.69596500	-0.47996700
C	1.36006700	3.70751100	0.48551300
H	1.63892500	3.75100700	-0.56858200
C	-5.13555600	-2.97108300	-0.04881800
H	-4.99884500	-2.67663800	-1.08990800
C	-0.39023700	1.96275800	-0.11736500
C	-3.81128000	-2.10924800	3.33432200
H	-3.95665900	-2.42040900	4.37205700
C	6.53342300	-0.29999300	-1.63598700
H	6.04627000	0.55708900	-2.10668700
C	0.33018100	2.83990300	0.86866100
C	-5.90606800	0.05483000	-0.52656600
H	-5.89826800	-0.20200100	0.53364400
C	6.44103900	-2.19874800	-0.14151700
H	5.87708300	-2.82108300	0.55736400
C	1.06687600	-1.76640400	1.75141000
C	0.79506200	-1.23262500	3.16114300
H	1.57341300	-0.51285800	3.45155700
H	0.78172000	-2.05196500	3.89721800
H	-0.17028900	-0.71410200	3.20327600
C	3.70245800	1.36738900	2.51751600
H	3.44139100	0.50082000	3.14170200
H	2.77495500	1.77539200	2.08857500
H	4.14067700	2.14062400	3.16506900
C	4.40070200	-0.79343300	-0.40793700
C	1.67672900	-2.37658400	-2.36401000
H	1.04542500	-1.47497300	-2.33733500
H	1.37362000	-3.01666500	-1.52336200
H	1.46925400	-2.91474600	-3.30055800
C	-7.08532200	0.90813500	-2.46367100
H	-8.00341100	1.30047000	-2.90749700
C	0.02197100	-2.81989100	1.36805600
H	-1.00548100	-2.43544500	1.41397800
H	0.07555400	-3.67827800	2.05524200
H	0.19461700	-3.17998600	0.34412600
C	4.69140200	0.96490900	1.42214000
C	-5.47413000	-3.78584100	2.60115000
H	-5.59093300	-4.10031500	3.64198700
C	3.16269700	-2.02348300	-2.27289900
C	-5.99156900	-4.00356900	0.25987400
H	-6.52853900	-4.51559100	-0.54244000
C	8.48415200	-1.69170400	-1.32556800
H	9.52742700	-1.92267500	-1.55369900
C	-6.17788600	-4.41075700	1.59909700
H	-6.86703900	-5.22575400	1.83287900
C	-0.00963000	2.75999700	2.22776900
H	-0.78781800	2.06304500	2.54654900
C	7.77022300	-2.49242600	-0.43326900
H	8.25251700	-3.35147400	0.03900100
C	5.91140200	0.30852900	2.07239200
H	6.71675300	0.11307500	1.35211600
H	5.63491700	-0.64298300	2.55192700
H	6.31174200	0.97766800	2.84888700
C	2.00113100	4.50984000	1.42685200
H	2.79749200	5.18628000	1.10636700

C	-2.46468800	3.50173500	-0.52703400
C	2.43389200	-2.44840000	1.75033900
H	2.66722700	-2.86883100	0.76299700
H	2.42904200	-3.26892400	2.48340300
H	3.23191900	-1.74608300	2.02412300
C	7.86417400	-0.59701400	-1.92652200
H	8.41924400	0.03140200	-2.62703600
C	0.63275600	3.56312900	3.16830200
H	0.34695500	3.49435500	4.22081600
C	3.55865500	-1.18273900	-3.49228700
H	4.62934800	-0.93233400	-3.47021300
H	2.97814100	-0.24783900	-3.51048800
H	3.36009700	-1.73595300	-4.42328300
C	-5.94984300	0.76537000	-3.22644100
H	-5.95145700	1.04976400	-4.28239900
C	1.63531800	4.44705800	2.77097600
H	2.13857300	5.07645600	3.50910000
C	5.11632700	2.21178200	0.63738900
H	5.59173500	2.94629100	1.30590900
H	4.23810100	2.68241600	0.17096600
H	5.83837600	1.95265800	-0.15090000
C	-3.34171800	3.14237500	0.67497000
H	-2.73362100	3.00141700	1.57962600
H	-4.06017500	3.95353100	0.86783000
C	3.98324500	-3.31444000	-2.22775200
H	3.72723000	-3.94284800	-3.09408000
H	3.75952600	-3.88632000	-1.31416300
H	5.06378500	-3.12091300	-2.26211700
C	-1.71988700	4.81597500	-0.28423900
H	-2.43583700	5.65059100	-0.33385400
H	-0.95258300	4.97590200	-1.05673000
H	-1.23330900	4.84870800	0.69893000
C	-3.33295300	3.63003700	-1.77766600
H	-3.85816900	2.68676100	-1.97975800
H	-2.71102300	3.87557300	-2.65329500
H	-4.07639700	4.43054000	-1.64798900
H	-3.89824600	2.21409600	0.48090600

VI.5.10. S_ Int 2 (2C-2)

0 1

S	2.98492600	1.97651000	-2.97622800
Si	-0.26969200	0.69048400	-0.38393500
Si	2.51376600	1.05497400	-1.25994500
O	-1.54054200	1.04010400	-1.41674700
O	-1.00501300	0.41883000	1.09805300
N	1.08424900	1.69487000	-0.37877300
N	3.78902300	-1.46720900	0.83914300
N	1.62496000	-0.72557400	-1.67774900
N	3.80307700	0.63812000	-0.12221900
C	-4.77679900	1.69467900	0.22342100
C	-3.56927400	0.99467600	-0.13133500
C	-2.75586100	1.55400200	-1.10820400
C	-3.23382300	-0.28373700	0.54710700
C	-2.78738300	-2.54233100	2.20470300
H	-2.63515600	-3.38577200	2.88302200
C	-1.84006400	-1.55947400	2.09856500
H	-0.91464300	-1.60213800	2.67424200
C	2.79613100	0.40453000	2.13195800
C	-2.03634700	-0.46125900	1.22605800
C	-5.18093600	2.84385100	-0.52604400
C	-4.16581200	-1.37889400	0.54205800
C	-3.94389300	-2.51114800	1.38598600
C	-3.17126900	2.67746800	-1.86408900
H	-2.51565000	3.02016200	-2.66614900
C	-6.15427600	-2.47528800	-0.33841800
H	-7.00521400	-2.47321400	-1.02394600
C	-1.50301800	-1.78859600	-2.51857500
H	-1.44588300	-0.92484200	-3.18285100
C	-5.58540600	1.30759100	1.32606500

H	-5.27893500	0.45944100	1.93912700
C	0.42020300	-0.84711900	-1.23628600
C	-4.36286700	3.29431800	-1.59338800
H	-4.68424900	4.15606500	-2.18384800
C	1.56640000	-0.07083200	2.59289600
H	1.08406000	-0.88803200	2.05853200
C	-0.57127800	-1.92804400	-1.48146900
C	-5.29433700	-1.40093200	-0.31938600
H	-5.46347300	-0.55745900	-0.99023600
C	3.36955800	1.49792200	2.79459700
H	4.30976300	1.91469500	2.43452800
C	0.93371400	3.16149100	-0.07477400
C	0.34964800	3.88392500	-1.29144700
H	0.98852300	3.71797600	-2.17106000
H	0.27874900	4.96495200	-1.09373200
H	-0.65970100	3.51541200	-1.51790000
C	2.53478100	-3.35650300	1.76858400
H	2.34757300	-3.68645000	0.73650200
H	1.66338100	-2.78581200	2.11727100
H	2.62440600	-4.25272500	2.40083800
C	3.47119700	-0.23981400	0.95420000
C	5.40982200	2.52819700	-0.23080100
H	5.00611700	2.95124200	0.69913200
H	4.87902000	2.97575300	-1.08244800
H	6.47055700	2.81326400	-0.30486700
C	-5.94606100	-3.58045000	0.51632100
H	-6.64127900	-4.42312400	0.49890800
C	-0.00045500	3.32183400	1.12582500
H	-1.01844600	2.95501600	0.91335100
H	-0.09330000	4.38734700	1.38374400
H	0.38284300	2.77753700	2.00099300
C	3.82277000	-2.52924000	1.84428400
C	-6.38015900	3.51932400	-0.18347800
H	-6.67500300	4.38935200	-0.77645500
C	5.27569800	1.00257700	-0.26849100
C	-6.73626700	1.99251900	1.64376000
H	-7.33153800	1.67515200	2.50342000
C	1.53572800	1.56278300	4.36697300
H	1.04624400	2.01310400	5.23375500
C	-7.15027600	3.10286900	0.87643000
H	-8.06953800	3.63408000	1.13393000
C	-0.69790500	-2.99361900	-0.58285000
H	0.00507500	-3.09086900	0.24611500
C	2.75791500	2.05835000	3.91224100
H	3.23309300	2.89946700	4.42278400
C	4.98845800	-3.43089700	1.40542900
H	5.94083300	-2.88198900	1.45507900
H	4.84291100	-3.75873800	0.36585400
H	5.07000500	-4.31783600	2.05276900
C	-2.51497500	-2.73240300	-2.67903200
H	-3.23677500	-2.61364100	-3.49018300
C	2.24712800	-1.70027700	-2.65598300
C	2.28231400	3.77866200	0.26862900
H	2.74833500	3.25447200	1.11136600
H	2.14315300	4.83379100	0.54754200
H	2.95461700	3.74652000	-0.59840900
C	0.93327700	0.50407700	3.69164700
H	-0.04442600	0.13646900	4.01099700
C	-1.71197200	-3.93150100	-0.74967400
H	-1.80153800	-4.75834400	-0.04188500
C	6.10638600	0.41279400	0.87810300
H	6.14848800	-0.68172700	0.82377000
H	5.73850400	0.68871500	1.87580400
H	7.13145800	0.80003700	0.78856800
C	-4.86180200	-3.59180600	1.36216000
H	-4.68053700	-4.44540700	2.02118400
C	-2.62112700	-3.80731600	-1.79889900
H	-3.42806800	-4.53420400	-1.91328100
C	4.08561400	-2.07929900	3.28786500
H	4.33358300	-2.95786600	3.90303600
H	3.22540500	-1.57905600	3.74854400

H	4.94059700	-1.38763500	3.32575500
C	1.93208700	-3.16426000	-2.34073100
H	0.90810200	-3.46197000	-2.58716600
H	2.61074100	-3.78217100	-2.94588000
C	5.92747700	0.50939700	-1.56271700
H	6.96297700	0.88193500	-1.59733600
H	5.40050200	0.89675100	-2.44442300
H	5.96705700	-0.58620900	-1.60092000
C	1.72049100	-1.31883000	-4.04368400
H	2.22764900	-1.94167400	-4.79592700
H	1.93507500	-0.26062600	-4.25297800
H	0.63939000	-1.49879100	-4.12509900
C	3.75445200	-1.53244600	-2.60664500
H	4.12264200	-1.70837000	-1.58604400
H	4.03619400	-0.52751800	-2.93633800
H	4.21814300	-2.25582500	-3.29266400
H	2.13139400	-3.39131000	-1.28451200

VI.5.11. S_ Int 2 (2C-3)

0 1

Si	-0.83030000	-0.09181600	0.00241200
Si	1.74015600	-0.20233800	-0.01140900
O	-1.95753900	-0.64420200	1.11906100
O	-1.78405700	0.34519700	-1.32007800
N	5.35968200	0.54556300	-1.23821100
N	3.06522200	0.19531600	-1.09789900
N	0.70743700	0.34700200	2.11859100
C	-3.04520100	-1.39120100	0.81228200
N	0.40143800	-1.17063800	-0.58511300
C	-3.11927900	-2.67708500	1.40164500
H	-2.32534000	-2.96414800	2.09386500
C	-5.24139100	2.62764300	-0.90384600
C	-4.04165300	-0.91696400	-0.02965000
C	-2.95729900	1.01839600	-1.20016700
C	0.65927800	2.48313300	0.74792300
C	-5.13728300	-3.13808900	0.14755700
C	0.12902300	0.66546700	3.43545900
C	-5.07523900	-1.83201100	-0.43326200
C	-6.37634800	3.45202000	-0.69277900
H	-6.40714900	4.43082200	-1.17922100
C	-4.04193800	0.48860500	-0.51121200
C	-3.03370700	2.28355600	-1.83375900
H	-2.17500300	2.60581700	-2.42603800
C	-5.18189000	1.33694800	-0.29009500
C	4.35499300	-2.60065600	-0.34457500
H	4.02523100	-2.73083100	-1.37664800
C	-7.41024400	3.04062400	0.11448400
H	-8.27686400	3.68650500	0.27393300
C	-0.26380700	3.32668500	0.12189200
H	-1.18607700	2.90856600	-0.28500600
C	-4.15474300	3.06035400	-1.70580400
H	-4.21352000	4.03200600	-2.20303600
C	-6.16769000	-4.03083200	-0.24425100
H	-6.20391700	-5.02022000	0.21991300
C	4.39680400	-0.11955500	-0.74711900
C	4.55792300	-1.30947800	0.15650700
C	2.81341200	1.08670100	-2.29091200
C	-6.25901900	0.95375100	0.55392100
H	-6.21723600	-0.01089000	1.06137400
C	4.61454400	-3.71828700	0.44535800
H	4.46187400	-4.71952800	0.03554900
C	-4.15217000	-3.52179400	1.09349100
H	-4.21217800	-4.51210500	1.55215500
C	6.79903700	0.33969300	-1.15092100
C	1.81381800	3.05860200	1.30443600
H	2.54652600	2.40916000	1.79044100
C	0.32949600	-2.51800500	-1.17858500
C	-6.03511100	-1.50338300	-1.42828000
H	-5.98411000	-0.52739500	-1.91237100

C	-7.33965900	1.78244900	0.75211900
H	-8.14850500	1.46415500	1.41433000
C	-1.09879800	1.56016400	3.26174000
H	-0.83093200	2.54086400	2.84555600
H	-1.55378200	1.72617500	4.24963400
H	-1.85173700	1.09108500	2.61615800
C	2.03772900	4.42919000	1.22343200
H	2.94634300	4.85498200	1.65686500
C	5.06655100	-3.55730400	1.75435200
H	5.26192800	-4.43242800	2.37864000
C	0.74193600	-3.54293800	-0.11845700
H	1.76383100	-3.35197600	0.24173000
H	0.69664900	-4.56848700	-0.51778200
H	0.06956900	-3.47542900	0.75004800
C	-0.28688100	-0.65696900	4.08933200
H	-1.05252200	-1.14742200	3.47242800
H	-0.69047500	-0.48869200	5.09993100
H	0.57577100	-1.33663200	4.17445000
C	0.46386400	1.00097700	0.83002600
C	-0.03578400	4.70007400	0.02988000
H	-0.77540400	5.33648900	-0.46292100
C	7.33389200	1.12733400	0.05111900
H	6.97622000	2.16742600	0.01685400
H	8.43491500	1.13714300	0.04502700
H	7.00405800	0.67954900	0.99874000
C	1.11518400	5.25810300	0.58051600
H	1.29299800	6.33421900	0.51526200
C	5.02779300	-1.15728700	1.46896000
H	5.19042400	-0.15556500	1.86840600
C	1.14476900	1.38931400	4.33009800
H	2.06640500	0.79737800	4.44754300
H	0.72150900	1.55353100	5.33314000
H	1.41571700	2.36695300	3.90664500
C	-7.09366400	-3.67105600	-1.19455900
H	-7.87990900	-4.36904600	-1.49164200
C	5.26878800	-2.27563900	2.26467300
H	5.61777000	-2.14291400	3.29132600
C	-7.01361200	-2.39733300	-1.79906700
H	-7.73268200	-2.11840100	-2.57317200
C	-1.09453800	-2.81035700	-1.65231000
H	-1.80376400	-2.85301600	-0.81430400
H	-1.12703800	-3.78807000	-2.15601800
H	-1.44178400	-2.04275200	-2.35856800
C	1.25474600	-2.59422200	-2.39544900
H	0.87765100	-1.95942400	-3.21087500
H	1.31594800	-3.62716500	-2.77018700
H	2.26813100	-2.25222500	-2.15130600
C	3.30410700	2.51500200	-2.02724500
H	2.78384700	2.94483500	-1.15910400
H	3.08011500	3.14469100	-2.90246600
H	4.38264800	2.52733800	-1.84024800
C	1.31200300	1.16920800	-2.57680000
H	0.85843900	0.19418700	-2.78837900
H	1.16555000	1.81046500	-3.45762900
H	0.75829700	1.65106700	-1.75763300
C	7.27882200	-1.11771500	-1.08782800
H	7.06989700	-1.60223300	-0.12583000
H	8.36811300	-1.14713400	-1.24471000
H	6.80788600	-1.71616200	-1.88270200
C	3.48197800	0.48493500	-3.53073300
H	4.57148900	0.45231600	-3.42410700
H	3.23884800	1.10129100	-4.40928000
H	3.10629000	-0.53296900	-3.71491600
C	7.36670700	0.96600500	-2.43430900
H	7.00899300	0.41927300	-3.32061700
H	8.46751400	0.94015800	-2.43717000
H	7.03627100	2.01099500	-2.52621000
S	2.26581800	-0.49483400	2.05420600

VI.5.12. CO_2_Int1

0 1

Si	-0.83926400	0.24002500	0.00545500
O	-1.99588100	0.73854200	-1.11524700
O	-1.69618600	-0.78805500	1.01009500
C	-3.25941900	1.13296300	-0.80548700
C	-4.14935000	0.30017800	-0.13937900
C	-3.87774500	-1.14586900	0.06982700
C	-2.72733200	-1.60312800	0.70066200
N	0.01332700	1.35537400	0.99620700
C	-0.66923800	2.36597100	1.88314000
C	1.45191900	1.48341300	0.71660600
Si	1.90777700	-0.06559500	-0.28792400
N	0.40686200	-0.50144900	-0.98555200
C	0.16339100	-1.36620500	-2.17922400
N	3.55166500	-0.15694200	-1.06807900
C	4.39418700	0.60859900	-2.01425100
C	3.92418400	-1.02649200	-0.10692900
N	2.82873800	-1.38448500	0.55654800
C	2.60300700	-2.38300800	1.62578400
O	3.64718700	1.35243600	1.59580200
C	2.44607400	1.16834400	1.91043800
O	1.98218000	0.63653300	2.92081900
C	1.40198800	-2.21727400	-2.46069000
H	2.28164200	-1.59694300	-2.68376600
H	1.64031000	-2.86236900	-1.60246700
H	1.22066400	-2.86328900	-3.33166400
C	-0.12979400	-0.46908400	-3.38484400
H	-1.03122700	0.13198200	-3.20559600
H	0.70830100	0.21841800	-3.57077900
H	-0.28625800	-1.07694900	-4.28944000
C	-1.01525100	-2.30600100	-1.93895800
H	-1.95390900	-1.75481900	-1.79194400
H	-1.15433400	-2.96074800	-2.81187600
H	-0.84468000	-2.94336100	-1.05952900
C	-1.22442300	3.54058400	1.06433600
H	-1.85896400	4.17103000	1.70659000
H	-0.42227900	4.17045400	0.65874600
H	-1.84584100	3.19373200	0.23028500
C	0.26702300	2.94841300	2.94206300
H	0.62546500	2.17942800	3.63303200
H	1.14054400	3.44037700	2.49101000
H	-0.28924500	3.72070600	3.49460400
C	-1.82820500	1.68056300	2.61189900
H	-2.63574400	1.37457500	1.92984200
H	-1.47344700	0.79196000	3.15086800
H	-2.26852100	2.38452900	3.33335000
C	3.44451500	1.35963000	-2.94233600
H	2.78856200	0.66331900	-3.48604000
H	2.82283400	2.07591200	-2.39092600
H	4.02450700	1.92506900	-3.68496400
C	5.28820900	1.60103900	-1.26912100
H	4.69853800	2.16997900	-0.53965300
H	6.09020400	1.08408600	-0.72522500
H	5.75357100	2.29279600	-1.98710500
C	5.23600900	-0.34959700	-2.86194800
H	5.75995900	0.22587700	-3.63929900
H	5.99727300	-0.86973600	-2.26741100
H	4.60231800	-1.09861500	-3.36207600
C	1.12311900	-2.27484300	1.98594900
H	0.89764000	-1.26697000	2.36744100
H	0.49051100	-2.48363700	1.11153500
H	0.87789000	-3.00436400	2.77122200
C	3.45516700	-2.10463800	2.86504500
H	4.52414100	-2.26283200	2.67020400
H	3.28097600	-1.07662000	3.21666600
H	3.15479500	-2.80478200	3.65950000
C	2.89831600	-3.77976500	1.07264500
H	3.95994100	-3.89605000	0.81252800
H	2.65892800	-4.53355400	1.83727700

H	2.28821300	-3.99432300	0.18173100
C	5.30538800	-1.44168200	0.22371400
C	5.90638500	-2.55160200	-0.37546500
C	5.98386500	-0.69213700	1.19301500
C	7.20109300	-2.91269800	-0.00826400
H	5.36581900	-3.12874900	-1.12923600
C	7.27939900	-1.06212100	1.54656000
H	5.46293600	0.15627700	1.65344800
C	7.88767600	-2.16700600	0.94984700
H	7.67459700	-3.78014000	-0.47363300
H	7.81470300	-0.48269000	2.30225800
H	8.90314000	-2.45213900	1.23559700
C	1.74677100	2.74286000	-0.10556800
C	2.73770300	3.67493100	0.22906600
C	0.94171700	3.02051600	-1.22328000
C	2.91449000	4.83356000	-0.52751600
H	3.38200100	3.46761100	1.08357900
C	1.12139000	4.17241000	-1.98397600
H	0.14088000	2.33039100	-1.50016300
C	2.11415100	5.08891200	-1.63949800
H	3.69159900	5.54592200	-0.23837700
H	0.47821200	4.35411600	-2.84889900
H	2.25880500	5.99710400	-2.22981200
C	-2.57974900	-2.96347200	1.06926500
H	-1.68569900	-3.23890800	1.63078700
C	-4.82491100	-2.12708100	-0.39445600
C	-5.38136400	0.87728100	0.33201200
C	-3.63346400	2.43564000	-1.21582600
H	-2.91746400	3.00457600	-1.81168500
C	-3.52955500	-3.88647300	0.72512700
H	-3.41534600	-4.93326700	1.01836000
C	-4.65534400	-3.50449000	-0.04874700
C	-5.60062900	-4.46274600	-0.49542500
H	-5.45805200	-5.50795600	-0.20745400
C	-5.92332900	-1.78748900	-1.22995600
H	-6.05299700	-0.75050000	-1.54155400
C	-5.73896000	2.20856100	-0.04812700
C	-4.85253700	2.95251700	-0.86784100
H	-5.13967700	3.95635600	-1.19064500
C	-6.66485800	-4.09445500	-1.28412500
H	-7.38397600	-4.84219600	-1.62669800
C	-6.81404600	-2.74294200	-1.66411200
H	-7.64292500	-2.45032800	-2.31331000
C	-6.25887800	0.17853600	1.20395800
H	-5.98746000	-0.82388100	1.53730800
C	-6.96199200	2.76320400	0.40849700
H	-7.22171300	3.77879900	0.09764500
C	-7.42987600	0.75050100	1.64634300
H	-8.07846600	0.19284400	2.32629100
C	-7.79702100	2.05087200	1.23633500
H	-8.73311800	2.49113900	1.58775000

VI.5.13. CO_2_Int2

0 1

Si	-0.88339200	-0.20595500	0.51899600
Si	1.88633400	-0.51543700	0.67388400
O	-2.17770300	-0.72312200	1.45731300
O	-1.60654900	0.02014200	-0.98888200
N	3.60459900	-1.21259100	0.65947100
N	2.77305400	-0.24420100	-1.07773500
N	-0.19677700	1.19907200	1.26565400
C	-3.28169600	-1.39048900	1.04356600
N	0.40266400	-1.33323900	0.26487600
C	-3.57870500	-2.59007700	1.73782700
H	-2.94531100	-2.85217700	2.58687700
C	-4.84213700	2.60813400	-1.29432600
C	-4.10090100	-0.93140600	0.01670200
C	-2.70448300	0.80022800	-1.12458300
C	1.86312700	2.48966600	0.58046700

C	-5.41474200	-3.03187400	0.22666000
C	-0.92665700	2.01917900	2.30689200
C	-5.14184800	-1.80533900	-0.45602200
C	-5.91174700	3.53952200	-1.31593800
H	-5.77295200	4.47963800	-1.85686300
C	-3.92477700	0.41386300	-0.58884100
C	-2.55380300	2.02195200	-1.82436500
H	-1.57984100	2.23691500	-2.26778000
C	-5.00299600	1.36420500	-0.60572300
C	6.06001700	0.32002700	-1.03204400
H	5.72048300	1.16638900	-0.43001200
C	-7.09180800	3.27916400	-0.66036800
H	-7.90605700	4.00737500	-0.67882600
C	1.12105200	3.29374400	-0.29260600
H	0.07216400	3.04321500	-0.46580300
C	-3.60438000	2.89492300	-1.92578000
H	-3.49118400	3.83673300	-2.46899300
C	-6.45257300	-3.87888700	-0.23950300
H	-6.65170400	-4.80632100	0.30446500
C	3.87451900	-0.75568900	-0.58161600
C	5.20827800	-0.77067600	-1.23294500
C	2.51611900	0.28110100	-2.43255500
C	-6.22738600	1.13928800	0.07936800
H	-6.35691400	0.21532000	0.64425200
C	7.32968700	0.32450500	-1.60588100
H	7.99282300	1.17763200	-1.44568500
C	-4.62807100	-3.38050400	1.35380700
H	-4.85497600	-4.30047000	1.89865800
C	4.50959500	-1.76124100	1.70077100
C	3.20083200	2.83481500	0.82296800
H	3.79246800	2.24161600	1.52467400
C	0.22733500	-2.76993800	-0.07828000
C	-5.90526000	-1.51161800	-1.61836600
H	-5.69009000	-0.59990700	-2.17693300
C	-7.24049500	2.07051400	0.05458200
H	-8.16724900	1.87298600	0.59884000
C	-2.31608300	2.38630400	1.78058000
H	-2.24928900	2.90626100	0.81355900
H	-2.80904800	3.06165100	2.49540800
H	-2.96635100	1.51179700	1.66040000
C	3.77343500	3.94972200	0.21349500
H	4.81391400	4.20755800	0.42823600
C	7.74983200	-0.75426300	-2.38299900
H	8.74604400	-0.74997000	-2.83151900
C	0.01356100	-3.55828800	1.21763000
H	0.88103100	-3.44874600	1.88479500
H	-0.13793700	-4.62964800	1.01185900
H	-0.86796400	-3.17967700	1.75175000
C	-1.07068500	1.23128400	3.61873400
H	-1.56948000	0.26876200	3.44884400
H	-1.67843100	1.80936000	4.33234700
H	-0.09663900	1.05185400	4.09254400
C	1.27399300	1.26512100	1.21057500
C	1.69206700	4.40764000	-0.90402600
H	1.08970000	5.02173800	-1.57847000
C	5.69551100	-2.52696100	1.10270100
H	5.36324400	-3.27969800	0.37268600
H	6.20386000	-3.05707000	1.92124100
H	6.43743800	-1.87648500	0.62426600
C	3.02298800	4.74252000	-0.65502900
H	3.47101600	5.61862000	-1.12976500
C	5.62769100	-1.84885400	-2.01676400
H	4.96180500	-2.70130700	-2.16756300
C	-0.20229600	3.33546700	2.61203800
H	0.83017700	3.18544300	2.95191500
H	-0.74648100	3.83325000	3.42866200
H	-0.19896500	4.01543700	1.75090600
C	-7.18556500	-3.55257100	-1.35589600
H	-7.97967100	-4.21499900	-1.70840700
C	6.89612800	-1.83818300	-2.59014400
H	7.22185800	-2.68386500	-3.20007900

C	-6.89555500	-2.36113100	-2.05624200
H	-7.45945100	-2.11138500	-2.95834300
C	-0.96553100	-2.96811200	-1.01541400
H	-1.91707900	-2.67173200	-0.55402500
H	-1.05273900	-4.03193200	-1.28211800
H	-0.84576600	-2.38254200	-1.93761500
C	1.47230100	-3.29935600	-0.79085200
H	1.64840600	-2.75099400	-1.72574600
H	1.33993600	-4.36383100	-1.03392800
H	2.36895300	-3.20913600	-0.16121200
C	3.30890900	1.56618100	-2.68606000
H	3.11657100	2.30886400	-1.90246600
H	3.00698100	2.00022100	-3.65127000
H	4.38932700	1.37296600	-2.73133500
C	1.02306500	0.57481100	-2.51015100
H	0.42547500	-0.33407900	-2.37264700
H	0.77660200	1.01119900	-3.48917100
H	0.72522100	1.29131900	-1.73617800
C	5.04205900	-0.62369800	2.57900900
H	5.58886100	0.10934000	1.96496800
H	5.74491100	-1.02230200	3.32664800
H	4.23395600	-0.10804400	3.11477100
C	2.85305800	-0.74984600	-3.51670100
H	3.93208900	-0.93075800	-3.59939600
H	2.50705200	-0.37269400	-4.49066100
H	2.34459500	-1.70694600	-3.32969700
C	3.71732600	-2.76683600	2.54402300
H	2.84897500	-2.29727300	3.01873300
H	4.36860300	-3.16660800	3.33519300
H	3.38444400	-3.61389000	1.92436400
C	1.85878200	0.83444800	2.57589300
O	2.26521400	1.49672400	3.49617500
O	1.88780000	-0.48720900	2.50225000

VI.5.14. CO_2_Int3

0 1

Si	-0.15199700	-1.45011200	-0.42277900
O	0.19650800	-0.04905800	0.44847500
O	-1.53120300	-1.03734500	-1.29077100
C	-0.77410200	0.63261500	1.10934100
C	-1.82910600	1.24437300	0.43983400
C	-1.87521100	1.31882700	-1.04275800
C	-1.77527800	0.17537300	-1.82842200
N	-0.81014900	-2.72998000	0.57605300
C	-0.02962900	-3.48611100	1.59971700
C	-2.12491300	-3.18327700	0.26402400
Si	2.77817200	-0.77276300	-1.25390900
N	1.27782200	-1.70993100	-1.36664600
C	1.23075000	-2.66286700	-2.55405100
N	3.36404700	-0.53634600	0.49032500
C	4.45528000	-1.24901000	1.18451300
C	3.20405200	0.79378400	0.34990000
N	2.58730700	1.01511100	-0.79785000
C	2.22021600	2.24913000	-1.50175400
O	3.89993800	-1.04092800	-2.29336100
C	-2.25405900	-4.31829400	-0.42868700
O	-2.36197500	-5.33935100	-0.97545700
C	0.86113900	-2.50193600	2.35520800
H	0.24285400	-1.80988000	2.94221700
H	1.52248400	-3.04365400	3.04772500
H	1.49926700	-1.90679700	1.68484000
C	-0.96606800	-4.14778100	2.61214500
H	-0.36334300	-4.60067000	3.41347700
H	-1.64968200	-3.41682900	3.06566700
H	-1.57245700	-4.94415700	2.15999400
C	0.82883600	-4.56256500	0.93105700
H	0.20218300	-5.29581500	0.39993400
H	1.51854800	-4.11017400	0.20633100
H	1.42198500	-5.11396900	1.67693600

C	1.25201100	-1.83995400	-3.84543000
H	1.15885700	-2.50003400	-4.72198500
H	0.41944000	-1.12104000	-3.86083700
H	2.20434600	-1.29505600	-3.92139800
C	2.42311500	-3.62626100	-2.53608100
H	3.36602000	-3.06839600	-2.61771200
H	2.42873100	-4.22659200	-1.61399400
H	2.34209300	-4.32139900	-3.38604300
C	-0.03798500	-3.51460300	-2.52507500
H	-0.03447700	-4.19222700	-1.66307200
H	-0.96008300	-2.91617300	-2.51506200
H	-0.06639900	-4.14321800	-3.42649600
C	1.36171500	1.81593800	-2.68813600
H	1.93415400	1.15839600	-3.35964100
H	0.46888000	1.27851300	-2.34754100
H	1.02966800	2.69400800	-3.25976000
C	3.48982100	2.92237700	-2.03546300
H	3.21997000	3.75125500	-2.70732600
H	4.09803500	3.33763100	-1.22014100
H	4.09317900	2.19274000	-2.59631100
C	1.43246500	3.19763800	-0.60029600
H	2.05405200	3.60709000	0.20782500
H	1.04798700	4.03909800	-1.19459600
H	0.57245000	2.67985200	-0.15449400
C	4.41103900	-1.01213600	2.69401100
H	3.42461700	-1.27495600	3.10387600
H	5.16758100	-1.64069700	3.18773300
H	4.62546200	0.03374900	2.95279200
C	4.25672100	-2.73549000	0.88510500
H	5.06035700	-3.32264800	1.35269700
H	3.29744000	-3.10025000	1.27190300
H	4.29227600	-2.91377900	-0.20053500
C	5.80605700	-0.81992100	0.59727600
H	6.04204200	0.22043800	0.86152100
H	6.61432400	-1.45367300	0.99318100
H	5.77420600	-0.91516000	-0.49955300
C	3.62730800	1.82550200	1.32928600
C	4.81857700	2.54080800	1.17633100
C	2.79861900	2.08378200	2.42573900
C	5.17868800	3.50177000	2.11952200
H	5.46710100	2.34277000	0.32108000
C	3.15842000	3.05012300	3.36045900
H	1.86175100	1.53661900	2.52880300
C	4.35068200	3.75867700	3.21131600
H	6.11309500	4.05428600	1.99709400
H	2.50024800	3.25207600	4.20869600
H	4.63423000	4.51532000	3.94673400
C	-3.35586400	-2.48480800	0.67767000
C	-4.56508900	-2.68147000	-0.01034600
C	-3.34731600	-1.60643000	1.76916800
C	-5.73189700	-2.04600200	0.40069800
H	-4.58742200	-3.32585700	-0.89436800
C	-4.51604500	-0.96500700	2.17273500
H	-2.41044500	-1.41286500	2.29379900
C	-5.71479700	-1.18284000	1.49667300
H	-6.65944600	-2.21238100	-0.15313700
H	-4.48559900	-0.27548300	3.01912200
H	-6.62547200	-0.66872900	1.81223900
C	-1.95951900	0.22632900	-3.23208000
H	-1.93516700	-0.71594300	-3.78152000
C	-2.00541600	2.58861700	-1.70789200
C	-2.88604500	1.81876000	1.22759500
C	-0.64803500	0.73340500	2.51692200
H	0.22224300	0.27182700	2.98361000
C	-2.15623000	1.42347700	-3.86538100
H	-2.29428900	1.45890000	-4.94895600
C	-2.15021500	2.63594400	-3.13007300
C	-2.75103400	1.91801500	2.64701200
C	-1.59178600	1.38656200	3.26471400
H	-1.47785500	1.47020100	4.34863000
C	-1.94917600	3.82638900	-1.01122800

H	-1.80540100	3.82228800	0.06941400
C	-2.26439800	3.88925300	-3.78461600
H	-2.38075100	3.89943500	-4.87175800
C	-4.10617200	2.25592100	0.64662700
H	-4.25163600	2.13903700	-0.42767600
C	-3.79361300	2.50042500	3.41307000
H	-3.66436500	2.57932100	4.49603800
C	-5.11159900	2.79223700	1.41754400
H	-6.04558000	3.10383600	0.94391200
C	-4.95270500	2.93369700	2.81394300
H	-5.75430900	3.37075600	3.41399600
C	-2.21794000	5.06644000	-3.07618600
H	-2.30156800	6.02593500	-3.59183200
C	-2.04932300	5.02786400	-1.67478500
H	-1.99186700	5.96110900	-1.10920500

VI.5.15. CO_2_Int4

0 1

Si	0.57073500	1.26392700	-0.19609200
O	1.64238600	1.00742400	1.21304800
O	1.71409600	0.56984300	-1.27419800
C	1.87987400	-0.25498600	1.58168900
C	2.77197000	-1.05575400	0.87153600
C	3.60889400	-0.43889800	-0.18156900
C	3.03394100	0.32682900	-1.19066100
N	0.41474200	3.00764800	0.09661200
C	1.52892000	3.95337900	0.40801500
C	-0.89924700	3.46075100	0.39357800
Si	-1.54110400	-0.00256500	-0.77996100
N	-0.71409000	1.12278100	-1.67002400
C	-0.64141500	1.43552600	-3.09690100
N	-1.86364600	-1.78447800	-1.06628000
C	-1.00381500	-2.98904900	-1.00811400
C	-3.13002800	-1.60269400	-0.68085200
N	-3.31938700	-0.28837400	-0.51201900
C	-4.46126000	0.50849600	-0.03165200
O	-0.63831400	0.24213200	0.56204700
C	-1.51109600	4.05371300	-0.63482800
O	-2.02142300	4.56517600	-1.54710200
C	-4.14591000	-2.65855300	-0.47592200
C	-4.43199200	-3.12105300	0.81177800
C	-4.83148800	-3.17581700	-1.57926100
C	-5.40465100	-4.10219900	0.99172300
H	-3.89063200	-2.71676400	1.66964800
C	-5.80818800	-4.14934200	-1.39041300
H	-4.59944400	-2.81158100	-2.58243800
C	-6.09519100	-4.61356200	-0.10645100
H	-5.62437000	-4.46729000	1.99740700
H	-6.34716100	-4.54966300	-2.25197400
H	-6.86030900	-5.37974200	0.03850300
C	-1.59850800	3.32158100	1.68406500
C	-2.80835600	3.99235300	1.94088400
C	-1.07767700	2.48759000	2.68539900
C	-3.48437200	3.81585400	3.14397000
H	-3.22616400	4.67022000	1.18950400
C	-1.75591400	2.31898900	3.88949000
H	-0.13694100	1.96865500	2.49988800
C	-2.96451500	2.97393000	4.12825100
H	-4.42187300	4.35059000	3.31825000
H	-1.32995800	1.66339000	4.65403500
H	-3.49172200	2.83854600	5.07551900
C	2.81429300	3.45414100	-0.24823800
H	3.14264300	2.49933900	0.18094200
H	2.68291600	3.33253600	-1.33324800
H	3.62049000	4.18450700	-0.08626500
C	1.74961200	4.09340100	1.91773600
H	0.84796300	4.47871800	2.41626000
H	2.00611600	3.11854000	2.35329800
H	2.57270000	4.79741800	2.11864100

C	1.20571600	5.32960800	-0.18344600
H	0.33553600	5.79478100	0.30208200
H	2.06298100	6.00445400	-0.04094700
H	1.00633700	5.25594000	-1.26412500
C	0.28952500	2.62711400	-3.33092800
H	1.29920000	2.39966700	-2.96658700
H	0.35009900	2.85476900	-4.40601500
H	-0.06725300	3.52580000	-2.81303800
C	-0.10877000	0.23250200	-3.88482500
H	-0.01245200	0.46726000	-4.95692600
H	0.87233700	-0.06791800	-3.49314700
H	-0.79776700	-0.62300100	-3.78872500
C	-2.04219900	1.78436400	-3.61065400
H	-2.73439500	0.93937200	-3.45010900
H	-2.44183000	2.66488000	-3.08889100
H	-2.02534900	1.99828000	-4.69098500
C	-0.81803500	-3.39463200	0.45584300
H	-1.76443500	-3.74183800	0.89680400
H	-0.08478600	-4.21066500	0.53542500
H	-0.43714900	-2.54583300	1.04250000
C	0.34780200	-2.59234400	-1.59711500
H	0.25870200	-2.32555100	-2.65860600
H	0.78891000	-1.73986500	-1.06113000
H	1.04999700	-3.43240300	-1.50237300
C	-1.59611400	-4.13526200	-1.82651700
H	-0.86563300	-4.95630700	-1.86984800
H	-2.51871800	-4.53406900	-1.38396300
H	-1.80788800	-3.81248700	-2.85737800
C	-4.39785800	0.56751300	1.49750200
H	-5.18827000	1.22537700	1.88739800
H	-4.53909800	-0.43242700	1.93405900
H	-3.42970100	0.96667700	1.83616600
C	-4.27224100	1.90771100	-0.61237100
H	-3.28671300	2.30575500	-0.33733200
H	-4.34642500	1.89615300	-1.70878800
H	-5.03607600	2.58907900	-0.21271600
C	-5.80135100	-0.05293800	-0.50499200
H	-6.05839800	-1.00535000	-0.02364500
H	-6.59164500	0.67012100	-0.25574400
H	-5.80632800	-0.19852200	-1.59587400
C	1.20216300	-0.77638300	2.71887100
H	0.54742100	-0.09980300	3.26971100
C	2.84349300	-2.45651800	1.17799200
C	5.04222200	-0.56727300	-0.15349200
C	3.82615200	0.84020200	-2.25192200
H	3.31450100	1.38656700	-3.04624200
C	1.35470300	-2.08393100	3.09313500
H	0.83482100	-2.47238700	3.97342800
C	2.14172300	-2.97184000	2.31227700
C	5.83048300	-0.03948200	-1.22385900
C	5.18000200	0.64638100	-2.28131100
H	5.77730800	1.03733400	-3.10931700
C	3.54778900	-3.38017200	0.35864800
H	4.06431200	-3.00917800	-0.52824000
C	2.20747100	-4.35724900	2.60506000
H	1.67332200	-4.72945400	3.48414500
C	5.73062800	-1.17346000	0.93265700
H	5.15698900	-1.54070600	1.78415600
C	7.24053400	-0.18782700	-1.19861500
H	7.82089300	0.21373300	-2.03414600
C	7.87053400	-0.80691100	-0.14462000
H	8.95821800	-0.91087900	-0.13408700
C	7.10234300	-1.28977800	0.93707500
H	7.60038100	-1.75478400	1.79159300
C	3.57371700	-4.72504500	0.65461200
H	4.11562900	-5.41173600	-0.00072400
C	2.90770700	-5.22339800	1.79629300
H	2.94393600	-6.29041200	2.02928200

VI.5.16. CO_2_Int5

0 1

Si	-2.24833600	-1.58077900	-0.46883700
Si	-0.42163700	0.94546400	-1.08655700
O	-1.61323300	-0.14782600	-1.10436900
O	0.73984800	0.73109700	-2.26686700
O	0.39456800	0.68383700	0.36214200
O	-4.22988900	1.85669400	-2.02789100
N	-2.03702600	-1.34234000	1.37683400
N	-3.45363400	-2.37049100	-1.10911800
N	-0.78926100	-2.54674000	0.09698800
N	-1.04022100	2.54325100	-1.31450700
C	1.59113900	1.27947800	0.60543400
C	-1.49040600	4.48272800	2.53710300
H	-0.59480100	4.83156500	3.05837600
C	3.90414800	1.74566300	0.04983100
C	-0.96906800	-2.13021700	1.35774100
C	-1.36235500	3.88549600	1.28547300
H	-0.37873900	3.76128900	0.83116000
C	-4.60482500	-2.35439400	-1.94970800
C	1.66177100	2.18529000	1.69126200
H	0.76976600	2.31760700	2.30431100
C	-2.48852000	3.42282000	0.59139500
C	3.96135500	2.69035500	1.12236700
C	2.71017500	0.97121400	-0.15739100
C	1.76072400	-0.15902700	-2.19222400
C	2.68664600	-0.12785400	-1.15654500
C	-3.75187100	3.59696900	1.18207400
H	-4.65166300	3.25121400	0.66398500
C	-0.17549400	-3.78642200	-0.40651300
C	-3.85589500	0.14500300	1.77711100
H	-4.36544100	0.80060100	2.49714500
H	-4.56788600	-0.60597600	1.40487300
H	-3.53339800	0.76487100	0.92855900
C	2.82465500	2.86042100	1.95382600
H	2.88093100	3.55444100	2.79650900
C	-1.21172500	4.92192700	-2.02282600
H	-1.11679200	5.28716300	-0.99124800
H	-2.28134600	4.84718700	-2.26336200
H	-0.77033200	5.67431900	-2.69238300
C	1.28876000	-3.91494500	0.01185300
H	1.40098200	-4.10202600	1.08825100
H	1.85031000	-3.00542000	-0.24282300
H	1.74965300	-4.75852400	-0.52356600
C	-2.64831400	-0.52656700	2.43080800
C	-0.67630900	3.15350200	-3.68673800
H	-0.14055800	2.21669000	-3.89020500
H	-0.30409300	3.92285500	-4.38099600
H	-1.74604400	2.99636100	-3.89823000
C	-2.74580000	4.63780900	3.12210900
H	-2.84681500	5.10606800	4.10373900
C	-3.35295500	2.28779000	-1.40556000
C	-0.48140100	3.59469500	-2.23306500
C	-3.87583500	4.19414700	2.43138500
H	-4.86870800	4.31425600	2.87224200
C	1.86754300	-1.09149900	-3.25127700
H	1.14117000	-1.02003300	-4.06272600
C	-2.31231100	2.75758500	-0.70619000
C	2.84784800	-2.04790800	-3.23799500
H	2.92928600	-2.76572200	-4.05797600
C	5.03779600	1.63496700	-0.79926100
H	5.00241600	0.94603000	-1.64411700
C	-0.27394500	-3.71011600	-1.92969500
H	0.14957300	-4.61816200	-2.38357700
H	0.28655600	-2.84394700	-2.30584300
H	-1.32693300	-3.61276300	-2.23492100
C	3.73047200	-2.15719400	-2.13369900
C	5.14597100	3.44245800	1.32995200
H	5.17399200	4.15230200	2.16115000
C	-0.06773400	-2.45193200	2.48941900

C	3.64121500	-1.19997700	-1.07494600
C	-1.68024100	0.53665800	2.95091200
H	-0.79863400	0.07886600	3.42308700
H	-2.18282000	1.17113400	3.69642500
H	-1.33906200	1.17907400	2.12996800
C	5.36438600	-2.43389700	0.12818200
H	5.98562200	-2.55743800	1.01855700
C	-5.05912500	-3.80307700	-2.18693500
H	-5.95657300	-3.85470600	-2.82532800
H	-5.28327900	-4.28670800	-1.22356400
H	-4.25227000	-4.37591300	-2.67033400
C	4.48034400	-1.38161300	0.05724200
H	4.40597800	-0.68073700	0.88999700
C	-5.75313900	-1.57883200	-1.28410200
H	-5.46018900	-0.52753700	-1.13649800
H	-5.97937900	-2.01692200	-0.29911400
H	-6.67259800	-1.59754300	-1.89289900
C	-3.13501500	-1.41461200	3.57964200
H	-3.73448500	-0.81619800	4.28227600
H	-2.29560300	-1.84597700	4.14157800
H	-3.76461200	-2.23109300	3.19489200
C	1.00258500	3.80937200	-1.92271400
H	1.59478700	2.90193700	-2.09379300
H	1.14384300	4.11894600	-0.87589700
H	1.40962100	4.60101000	-2.56960700
C	-0.99709500	-4.98012900	0.09286300
H	-0.92521700	-5.07637200	1.18713300
H	-0.62638100	-5.91650700	-0.35184900
H	-2.05361500	-4.84420600	-0.18490000
C	6.23194400	3.29630100	0.49964600
H	7.13694900	3.88527900	0.66586500
C	1.15441700	-1.77451400	2.57255700
H	1.39971100	-1.02197400	1.82268000
C	6.16730700	2.39077400	-0.58203200
H	7.02044000	2.29133800	-1.25748700
C	5.47215100	-3.35744400	-0.93478200
H	6.18248800	-4.18486100	-0.86990800
C	-4.28905700	-1.70307800	-3.30612400
H	-3.97527300	-0.65816900	-3.15939900
H	-5.16144300	-1.71159500	-3.98118500
H	-3.46365600	-2.24149000	-3.79792800
C	4.66829200	-3.21718500	-2.04129400
H	4.72612600	-3.93470600	-2.86421200
C	-0.38808100	-3.43331500	3.43142700
H	-1.33739700	-3.96812700	3.35916100
C	2.04400100	-2.07292700	3.60009700
H	2.99547200	-1.53928100	3.65933900
C	1.72591300	-3.05362500	4.54014500
H	2.42834200	-3.29141200	5.34262000
C	0.51174400	-3.73378500	4.45279600
H	0.26158100	-4.50615300	5.18379400

VI.5.17. Compound 5

0 1

Cl	2.83478900	0.41805700	-2.15676700
Cl	3.52235400	2.25699300	2.52220800
Si	1.69251000	0.02015600	-0.44195000
Si	-1.10455800	0.07751100	-0.08222200
O	-1.97865200	-0.78447200	1.06076800
O	-2.24774900	0.38531500	-1.28172300
N	0.20335400	-0.75388900	-0.88819500
N	2.84795100	-0.95974600	0.54188700
N	4.64453500	0.48633700	0.46186000
H	4.05155800	1.15654600	1.07531800
N	-0.33914600	1.37191600	0.72679700
C	-4.18497100	-1.14650100	0.17231100
C	-2.94703300	-2.90982500	1.33375300
H	-2.05010500	-3.16951200	1.89935800
C	-3.04632000	-1.58718200	0.83709200

C	0.44116200	3.88028800	-2.77323600
H	-0.23114300	3.92664300	-3.63425200
C	4.20079800	-0.72932700	0.35886100
C	-3.94418200	-3.81856700	1.10258500
H	-3.86688600	-4.83643200	1.49340900
C	0.29763400	2.85688900	-1.83932500
H	-0.48917700	2.11053400	-1.97554600
C	6.17251400	-2.19725200	0.86681700
H	6.37797000	-1.59471500	1.75325100
C	1.02996100	1.64827600	0.27145800
H	1.67679300	1.87301900	1.13862100
C	-4.37472900	0.28202600	-0.19195900
C	-6.29915100	-1.81068800	-0.99272900
H	-6.38853500	-0.80399200	-1.40281800
C	1.15073000	2.77515400	-0.73240900
C	-5.52618500	1.01109000	0.26578600
C	2.29231700	4.76787500	-1.51200800
H	3.07098700	5.52205600	-1.37006800
C	-3.43799600	0.96014200	-0.95859300
C	-5.74883700	2.34798900	-0.19213800
C	2.14701900	3.74409900	-0.57707900
H	2.79171100	3.69398700	0.30521900
C	-5.19010200	-2.12096400	-0.16014100
C	6.81898900	1.29193500	1.23148700
H	7.23125000	0.32377100	1.54856700
H	6.25794700	1.73787400	2.06689600
H	7.66187300	1.95063700	0.97525500
C	5.08543300	-1.87635100	0.04541000
C	-0.28621400	-1.03197700	-3.30068300
H	0.55603200	-0.37583700	-3.56229200
H	-1.19504100	-0.42045700	-3.22532400
H	-0.42374200	-1.76149300	-4.11416700
C	-5.07455300	-3.46206900	0.32324900
C	5.46130600	2.53633400	-0.45217000
H	6.34157700	3.10242600	-0.78877500
H	4.98673700	3.07839500	0.37793900
H	4.74589400	2.46578900	-1.28391300
C	1.21553900	-2.64895100	-2.13521400
H	2.08276300	-2.07457500	-2.48597600
H	1.01306000	-3.42910300	-2.88340700
H	1.47110600	-3.14309500	-1.18830700
C	-0.01495800	-1.75710700	-1.97808800
C	6.69960100	-4.07126300	-0.56055600
H	7.33064900	-4.93127700	-0.79731900
C	-6.89181100	3.05949900	0.25470000
H	-7.04947300	4.07649200	-0.11456300
C	4.80588700	-2.66522300	-1.07496300
H	3.95302400	-2.41077300	-1.70375400
C	6.58277900	0.42252100	-1.14367700
H	7.35381000	1.09206600	-1.55144300
H	5.86321800	0.20480100	-1.94647100
H	7.07643400	-0.51157600	-0.85014400
C	-7.24916200	-2.75822500	-1.29920300
H	-8.08631300	-2.49114700	-1.94874600
C	5.62108900	-3.74884200	-1.38526400
H	5.40865800	-4.35091500	-2.27157800
C	-0.32963000	3.60160600	1.82616400
H	0.76306800	3.67836100	1.91585200
H	-0.76564200	4.17061800	2.66083800
H	-0.64301000	4.07338500	0.88386300
C	-0.81624200	2.15141400	1.91143100
C	-2.34362000	2.17711000	1.95949000
H	-2.76242300	2.65552300	1.06257700
H	-2.66656200	2.76321200	2.83226000
H	-2.77602600	1.17387000	2.05984900
C	-1.20025600	-2.65679600	-1.62924800
H	-1.33815000	-3.41528100	-2.41393000
H	-2.13905500	-2.09021700	-1.55991700
H	-1.03650800	-3.18098000	-0.67621500
C	-0.27390800	1.51821200	3.19644700
H	-0.67126400	0.49981700	3.32045700

H	-0.58086900	2.11083200	4.07213700
H	0.82761800	1.49000000	3.19125800
C	2.47831500	-1.86789500	1.70214100
C	0.99157900	-1.72108300	1.99750600
H	0.70373900	-0.66804100	2.11414300
H	0.76677900	-2.22267200	2.94930600
H	0.36692900	-2.17283200	1.21905600
C	3.26342000	-1.39402800	2.92914800
H	2.93080500	-1.94512200	3.82112600
H	3.12880600	-0.31363700	3.09619700
H	4.34079600	-1.58518800	2.81616300
C	2.76294500	-3.34788400	1.42891400
H	2.28246700	-3.68414800	0.50005200
H	2.34645700	-3.94132200	2.25686300
H	3.83339800	-3.57683500	1.36636600
C	-6.07845900	-4.41160700	0.00340500
H	-5.97678000	-5.42840300	0.39260100
C	-6.44766400	0.46802700	1.20119200
H	-6.27614800	-0.53504400	1.59356300
C	-3.66535300	2.27727800	-1.42316500
H	-2.90005800	2.73906600	-2.04924600
C	5.90059600	1.14280100	0.01564300
C	1.44210700	4.83949600	-2.61489800
H	1.55447400	5.64344000	-3.34673700
C	-4.80606500	2.94648800	-1.06707700
H	-4.98451800	3.96323500	-1.42610700
C	6.96959600	-3.29908700	0.56988400
H	7.80615600	-3.55587800	1.22361800
C	-7.77519700	2.49466400	1.14377000
H	-8.64855400	3.05504600	1.48553500
C	-7.53848600	1.18982900	1.62909400
H	-8.22479600	0.74899900	2.35621300
C	-7.14982400	-4.07072200	-0.78794200
H	-7.91460400	-4.81195000	-1.03145700

VI.5.18. CO_2_1^M

0 1

Si	-1.39125400	-0.23008300	-0.00056800
O	-1.99106600	0.27544300	-1.48235500
O	-2.51638600	-1.30567900	0.61970300
C	-3.16782200	0.93461500	-1.58022600
C	-4.35521300	0.48754900	-1.13044700
C	-4.63350500	-0.75767900	-0.42859100
C	-3.80689300	-1.52532800	0.30894600
N	-1.08391800	1.04888300	1.09958400
C	-1.63211100	1.19277500	2.42829600
H	-0.99177800	0.70611200	3.19206500
H	-2.63262900	0.73764000	2.47525400
H	-1.73563400	2.25575500	2.70542900
C	0.25165400	1.47384100	0.80544300
C	0.63359200	2.88506500	1.11719100
H	1.65981100	3.09972300	0.77583500
H	-0.04937400	3.59287500	0.60903200
H	0.60254700	3.13864100	2.19509400
Si	1.30558300	0.14446900	0.31340000
N	0.11967700	-1.03527700	-0.20209600
C	0.28980700	-2.40507000	-0.62574800
H	-0.61982400	-2.99284400	-0.42067200
H	0.50129800	-2.49094900	-1.70754300
H	1.11883900	-2.89709500	-0.09056200
N	2.65592400	0.69009000	-0.80537900
C	2.87098000	1.70578000	-1.79206600
H	3.73104200	2.35217500	-1.54477900
H	3.03329100	1.28491800	-2.80014200
H	1.97281700	2.33619100	-1.83345100
C	3.52471400	-0.22425900	-0.36262400
C	4.94084700	-0.33278300	-0.80055200
H	5.44337500	-1.19578200	-0.34743700
H	4.98670800	-0.42630400	-1.89621200

H	5.49006100	0.58332700	-0.52965200
N	2.89663400	-0.95624500	0.53850400
C	3.43272100	-2.00604800	1.35007700
H	3.61209800	-2.93038900	0.77245500
H	2.70560900	-2.24608700	2.13849800
H	4.37623400	-1.72231800	1.84828700
H	-3.09396100	1.89369900	-2.10594900
H	-5.21462900	1.12704700	-1.35147800
H	-5.66288800	-1.12358800	-0.48316600
H	-4.18485800	-2.44515800	0.76818600

VI.5.19. $CO_2\_TS_1^M$

0 1

Si	1.62731100	-0.28598800	-0.05371300
O	2.47296300	-1.36071700	0.91740900
O	2.64635900	0.08404200	-1.32449200
C	3.66747000	-1.04498400	1.46979300
C	4.76594100	-0.66825400	0.78935500
C	4.89851600	-0.48939700	-0.64955100
C	3.94575600	-0.18260800	-1.55115700
N	1.08102000	1.14394200	0.71611400
C	1.62817300	2.46812400	0.53502400
H	1.05409200	3.05921500	-0.20434500
H	2.66495400	2.39855700	0.17482200
H	1.64255900	3.02874100	1.48505300
C	-0.26633900	0.95369600	1.15570700
C	-0.76855800	1.73311900	2.32780800
H	-1.78613500	1.41219500	2.60142800
H	-0.11485200	1.58164600	3.20789000
H	-0.83108700	2.82298200	2.15166900
Si	-1.12929000	-0.23691900	0.18562400
N	0.18301000	-1.04889400	-0.61540700
C	0.15703900	-2.06872700	-1.64148000
H	0.79979500	-1.79864000	-2.49666800
H	0.50115100	-3.04666500	-1.26117900
H	-0.86406700	-2.20856900	-2.02879400
N	-2.48845900	-1.07915900	1.07977900
C	-2.78800700	-1.50972900	2.41333500
H	-3.68511500	-1.01145700	2.81911700
H	-2.93773000	-2.60173900	2.47747700
H	-1.93764400	-1.24977800	3.05749600
C	-3.29455000	-1.09407300	0.01045500
C	-4.69834200	-1.57689100	0.00902500
H	-5.11683900	-1.60920200	-1.00400900
H	-4.75158500	-2.58135700	0.45450300
H	-5.32023800	-0.91086400	0.62921700
N	-2.61741400	-0.56111700	-0.98977700
C	-3.05395100	-0.30545900	-2.33084500
H	-4.06209200	0.14057600	-2.36593700
H	-3.05560300	-1.22102200	-2.94805600
H	-2.35726700	0.40940600	-2.79015800
O	-2.36361200	2.98304000	-0.09193300
C	-1.57250200	2.49396700	-0.80228900
O	-0.89527600	2.24592700	-1.72952700
H	3.69324100	-1.15708400	2.55990200
H	5.66713300	-0.51627800	1.39043800
H	5.90558700	-0.59208200	-1.06380500
H	4.20499800	-0.08233600	-2.61051100

VI.5.20. $CO_2\_I_1^M$

0 1

Si	1.66152700	-0.11653000	0.02190200
O	2.70151300	-0.29403300	1.32020200
O	2.59988100	-0.29362700	-1.33979500
C	3.92414800	0.27580700	1.44554300
C	4.93492200	0.14889700	0.56772100
C	4.90781100	-0.57134200	-0.69665800

C	3.85210100	-0.77274000	-1.50571300
N	0.69931300	1.27826600	-0.12177200
C	1.21018700	2.57621300	-0.52753200
H	0.49234800	3.04599900	-1.21431500
H	2.16841500	2.44179600	-1.05153600
H	1.38581900	3.24713400	0.33350900
C	-0.64377500	1.20028400	0.47373200
C	-0.66161600	1.65407700	1.93594700
H	-1.68619600	1.64948800	2.32752900
H	-0.01128100	1.03438200	2.57591600
H	-0.29150800	2.69045000	1.99589200
Si	-1.05146100	-0.60346500	0.20004500
N	0.46233500	-1.37332300	0.21118000
C	0.78814500	-2.78993700	0.19468800
H	1.13883200	-3.11826200	-0.79830400
H	1.58197200	-3.01239300	0.92515700
H	-0.08973900	-3.39788600	0.45959800
N	-2.56034600	-1.19509600	0.99123400
C	-3.18668600	-0.92564400	2.26407100
H	-3.55668500	0.11232600	2.29687500
H	-4.01677300	-1.62457400	2.44926100
H	-2.44728500	-1.06737300	3.06246100
C	-3.18248500	-1.02430700	-0.19562800
C	-4.62302900	-0.78883200	-0.39034200
H	-4.95845900	-1.11281300	-1.38292000
H	-5.22945100	-1.25796500	0.39320000
H	-4.70859700	0.31364200	-0.32058900
N	-2.23469800	-0.94815800	-1.12702600
C	-2.39712900	-0.58438700	-2.51559800
H	-3.29344800	-1.06231100	-2.93690900
H	-1.52312800	-0.94071300	-3.07667000
H	-2.45752100	0.51290700	-2.61684200
O	-2.93636900	1.68553100	0.23907100
C	-1.83727300	1.83044600	-0.35111900
O	-1.59931900	2.25014300	-1.48674900
H	4.05188700	0.83168700	2.38095000
H	5.88306300	0.60807300	0.86157200
H	5.85795100	-0.97347700	-1.05969000
H	3.96506600	-1.32733200	-2.44322400

VI.5.21. CO_2_TS_2^M

0 1

Si	1.65834100	-0.10277200	-0.00807900
O	2.66178700	-0.47669700	1.28006200
O	2.61622000	-0.22240600	-1.36544900
C	3.93962100	-0.07059000	1.46222000
C	4.94635800	-0.24326800	0.58721500
C	4.85769800	-0.84425100	-0.73510000
C	3.79981000	-0.84057100	-1.56625600
N	0.83047500	1.38054800	-0.01546300
C	1.47848900	2.67289400	-0.16519200
H	0.81560700	3.34286100	-0.72997700
H	2.41592400	2.54508900	-0.72750000
H	1.72706900	3.14083900	0.80604900
C	-0.56290300	1.33280500	0.43021700
C	-0.72573500	1.74997200	1.90013100
H	-1.78056700	1.69933100	2.20291400
H	-0.11222600	1.13061700	2.57591200
H	-0.39960800	2.79615000	2.01621000
Si	-1.12876000	-0.42566900	0.12587500
N	0.36197800	-1.26072700	0.04668200
C	0.57759100	-2.69084500	-0.09091600
H	0.87451800	-2.96617900	-1.11734000
H	1.37025500	-3.03372300	0.59337800
H	-0.33853600	-3.24967900	0.15409500
N	-2.51505600	-1.15915600	1.03207500
C	-3.06131900	-1.09083800	2.36065300
H	-3.59445700	-0.14072200	2.53193200

H	-3.74953200	-1.92897200	2.55188300
H	-2.24154400	-1.16203800	3.08692100
C	-3.21754500	-1.14703100	-0.11068200
C	-4.68702300	-1.12780200	-0.22590600
H	-5.02786300	-1.57624600	-1.16724900
H	-5.18024700	-1.60965500	0.62654400
H	-4.94403700	-0.05261300	-0.23699500
N	-2.35444900	-0.97272800	-1.10880900
C	-2.69579300	-0.54152500	-2.44135600
H	-3.45459700	-1.20194400	-2.88789600
H	-1.79694400	-0.58458100	-3.06987700
H	-3.06468000	0.49780600	-2.41605100
O	-2.78122600	1.54788900	-0.18398200
C	-1.65221300	2.07514000	-0.42667800
O	-1.35243100	2.97938900	-1.19236700
H	4.11557700	0.38532400	2.44301700
H	5.93618000	0.07219300	0.92922400
H	5.75793800	-1.32896600	-1.12368000
H	3.85999600	-1.32221300	-2.54824700

VI.5.22. $CO_2\_I_2^M$

0 1

Si	1.54753100	-0.12187100	0.00547100
O	2.28810800	-0.80184200	1.34999500
O	2.67522000	-0.27084300	-1.21916000
C	3.58007000	-0.68225500	1.72554200
C	4.64908100	-0.97818300	0.96382400
C	4.63113100	-1.42306300	-0.42142100
C	3.72034000	-1.11069100	-1.36177300
N	1.06086400	1.50450400	0.08885000
C	1.92547900	2.65148100	-0.09179400
H	1.51122600	3.33572000	-0.85043900
H	2.91333500	2.31657100	-0.44122900
H	2.07280600	3.22258500	0.84281900
C	-0.37583500	1.70618900	0.25120600
C	-0.72981200	2.60747500	1.42132600
H	-1.81970200	2.67346200	1.56477000
H	-0.27216900	2.23153100	2.35016200
H	-0.36561500	3.63440800	1.25120400
Si	-1.30573100	0.05243500	-0.17502900
N	0.08383800	-0.99220400	-0.24161800
C	0.11937300	-2.42365800	-0.48598600
H	0.31417300	-2.66413900	-1.54669200
H	0.91141400	-2.90117300	0.11380800
H	-0.83224600	-2.89917500	-0.20521400
N	-2.56372900	-0.25106000	1.14438100
C	-2.75141800	-0.00007700	2.54464500
H	-3.65818900	0.59701700	2.74583700
H	-2.81664900	-0.93381600	3.12995300
H	-1.89180400	0.56520600	2.92368500
C	-3.29103700	-1.08172300	0.37988600
C	-4.57163300	-1.70244200	0.80862200
H	-5.30634600	-0.91630700	1.04013400
H	-4.98501900	-2.36662400	0.04082600
H	-4.41103200	-2.28126500	1.73082700
N	-2.66584300	-1.20572100	-0.76386300
C	-3.12547500	-1.75028700	-2.00321800
H	-3.17401900	-0.95118900	-2.76061600
H	-4.12284300	-2.20939200	-1.91560700
H	-2.42586900	-2.51804300	-2.37106900
O	-1.72962300	1.12860100	-1.49825500
C	-1.01400700	2.19348500	-1.06328000
O	-0.93769700	3.24806500	-1.62682800
H	3.70370800	-0.36219700	2.76674700
H	5.62295400	-0.90628100	1.45673200
H	5.46035600	-2.05263000	-0.75689600
H	3.81482300	-1.50014700	-2.38167900

VI.5.23. CO_2_TS_3^M

0 1

Si	1.24352000	-0.06612400	-0.30115300
O	1.48569000	-1.53169700	0.48920500
O	2.55673000	0.10100100	-1.31909000
C	2.60644700	-1.82640900	1.18894500
H	2.41238300	-2.12764300	2.22535400
C	3.85923200	-1.81873300	0.69691600
H	4.64130600	-2.15423900	1.38419800
C	4.27465000	-1.46231800	-0.65189100
H	5.21283600	-1.89711300	-1.00766300
C	3.66568200	-0.63374800	-1.52172600
H	4.09968000	-0.45916600	-2.51189100
N	1.18564600	1.26511100	0.80161300
C	2.34551400	1.87701200	1.41784000
H	2.63234200	2.81750900	0.91174900
H	3.20399800	1.19073400	1.36772400
H	2.16044800	2.10289100	2.48145700
C	-0.05585100	1.97947700	0.77316500
C	-0.78220400	2.41834700	2.01801000
H	-1.77867100	2.78260300	1.72843800
H	-0.91080600	1.58561000	2.72039000
H	-0.24809900	3.22478400	2.54920400
Si	-1.68819200	0.52649400	-0.34966800
N	-0.22669500	-0.07682900	-1.15778700
C	-0.34372800	-0.85493800	-2.38447400
H	-1.08634800	-0.40202200	-3.06057600
H	0.61493800	-0.86597400	-2.92740000
H	-0.64257000	-1.90375600	-2.20537400
N	-1.85024700	-0.58338100	1.16643700
C	-1.17952000	-0.92670500	2.39005400
H	-0.20064300	-0.43133900	2.42110400
H	-0.99043900	-2.01006000	2.46195800
H	-1.75290300	-0.61369600	3.28074100
C	-2.70926900	-1.37727700	0.52308700
C	-3.32897100	-2.59927500	1.10415200
H	-4.14566000	-2.97606700	0.47681700
H	-2.56590900	-3.38970600	1.18574400
H	-3.70880700	-2.39611800	2.11536100
N	-2.87113900	-0.90120300	-0.69526800
C	-3.88524700	-1.18994000	-1.66183200
H	-4.70480600	-1.79304600	-1.24008400
H	-4.30969400	-0.24089300	-2.02479800
H	-3.47288400	-1.73201800	-2.52984900
O	-2.45511200	1.86171200	-0.61038900
C	-0.16968800	2.73375100	-0.36667700
O	-0.04724700	3.24426000	-1.39091200

VI.5.24. CO_2_I_3^M

0 1

Si	0.73075900	-0.57445200	0.26267500
O	-0.44903500	-1.00484900	1.38185200
O	1.62562700	-1.95162300	-0.08670800
C	-1.19977900	-2.12694800	1.38982300
H	-2.02626600	-2.05647700	2.10495600
C	-1.06028700	-3.25206500	0.65913500
H	-1.83578000	-3.99713600	0.85844000
C	-0.06005000	-3.65186800	-0.32153900
H	-0.27268000	-4.57748500	-0.86234300
C	1.11837800	-3.07544900	-0.62955500
H	1.78114500	-3.53228000	-1.37237300
N	1.73722300	0.54266700	1.08311200
C	1.90387700	0.67199700	2.51574300
H	2.84755100	0.22118000	2.87122900
H	1.07071800	0.16695800	3.02550400
H	1.90021500	1.73307800	2.81968400
C	2.66391200	1.22025200	0.23293400
C	2.41040400	2.61981400	-0.24718000

H	3.25026500	3.00433300	-0.84413100
H	1.50774000	2.65640500	-0.88081600
H	2.27004700	3.30664200	0.60453300
Si	-1.20608900	1.17695400	-1.31641800
N	0.09513800	0.01372700	-1.20951000
C	0.72080300	-0.35647300	-2.48398300
H	0.35149100	0.32297100	-3.26662000
H	1.81779300	-0.25929500	-2.44403300
H	0.47928500	-1.39313400	-2.77529000
N	-1.49836200	1.73398800	0.42178900
C	-1.01325000	2.64746800	1.41283400
H	-0.14381700	3.17972400	1.00963500
H	-0.68938600	2.12571200	2.32948000
H	-1.76973700	3.40294300	1.68736900
C	-2.55762100	0.92850000	0.53611600
C	-3.35067300	0.76978800	1.77999500
H	-4.19131900	0.07848600	1.65220700
H	-2.68543700	0.39126500	2.57113400
H	-3.73314100	1.74821200	2.10704700
N	-2.72602000	0.32035400	-0.62367200
C	-3.61722000	-0.74097700	-0.97990100
H	-4.49227400	-0.78039700	-0.31285300
H	-3.98163500	-0.58333900	-2.00546000
H	-3.10648300	-1.71847400	-0.93780600
O	-1.28740500	2.09419800	-2.54850300
C	3.75749600	0.54573300	-0.11608700
O	4.70512800	-0.05631400	-0.41903500

VI.5.25. $CO_2\_TS_4^M$

0 1

Si	-1.10810700	0.43132700	0.22167200
O	-1.08510600	1.76581400	1.25677600
O	-2.02829700	1.08651900	-1.07372200
C	-0.23999700	2.78741400	0.99768000
C	-0.13882000	3.42971500	-0.18552100
C	-0.91184000	3.21669800	-1.40079000
C	-1.74486500	2.20143400	-1.73681300
N	-2.19240800	-0.81249800	0.72046800
C	-2.30667100	-1.25960300	2.09106800
C	-2.67228100	-1.69706600	-0.28324400
Si	1.40067900	-0.36315000	0.33684000
N	0.56390000	-0.34855400	1.70785500
C	0.70747800	-0.22917500	3.11882400
N	3.00325400	0.43528200	-0.09478200
C	3.59644600	1.74185000	-0.12547500
C	3.46630700	-0.70805800	-0.58864000
N	2.52773700	-1.63175900	-0.39192200
C	2.51241700	-3.00361700	-0.80654000
O	0.23956700	0.05227000	-0.75354900
C	-3.80207600	-1.33697500	-0.88247500
O	-4.78568200	-1.01052700	-1.41222300
C	-1.90559900	-2.92754000	-0.67753800
H	-2.43583200	-3.51686800	-1.44040200
H	-0.92083100	-2.64452300	-1.08990200
H	-1.73382100	-3.58464300	0.19224400
C	4.76811200	-0.90338100	-1.27314100
H	5.13789200	-1.92804000	-1.13701300
H	4.63279500	-0.73349400	-2.35383300
H	5.51683800	-0.18945900	-0.90660300
H	1.74083600	-3.53571800	-0.23380300
H	2.26992100	-3.11111700	-1.87768600
H	3.47627000	-3.50226800	-0.61345100
H	4.42474000	1.83999100	0.59681900
H	2.82022100	2.47355600	0.13789900
H	3.96971700	1.99873400	-1.12998200
H	0.13876400	0.64487800	3.49074800
H	1.75230500	-0.09176200	3.45784300
H	0.31481500	-1.11000200	3.66276200
H	-2.06102100	-0.43500800	2.77449300

H	-3.34392600	-1.57191400	2.29690900
H	-1.63174700	-2.10272300	2.32993700
H	0.37114600	3.09047600	1.85717300
H	0.57375400	4.26054000	-0.20434600
H	-0.83613000	4.00616000	-2.15331400
H	-2.28561800	2.26865000	-2.68962000

VI.5.26. $CO_2\_I_4^M$

0 1

Si	-1.32443700	0.01444200	-0.16761400
O	-1.85117400	-1.03170200	-1.41295200
O	-2.61156700	-0.41224700	0.95294700
C	-1.57113500	-2.34407900	-1.35834700
H	-0.94580000	-2.71811700	-2.18282600
C	-2.03225000	-3.17990000	-0.40436600
H	-1.77353900	-4.23786200	-0.51300000
C	-2.91211500	-2.81386000	0.69324400
H	-3.47859100	-3.62869400	1.15354100
C	-3.16420000	-1.58696700	1.21544500
H	-3.92296400	-1.51891600	2.00820400
N	-1.82991700	1.68544600	-0.27035900
C	-3.18701900	2.13380500	-0.01463200
H	-3.28906400	2.62637000	0.96983100
H	-3.87951500	1.28394000	-0.03494300
H	-3.50488600	2.85539200	-0.78742400
C	-0.92179700	2.72843800	-0.59221700
C	-0.77907300	3.23090100	-1.99677100
H	-0.08770900	4.08437300	-2.06739900
H	-0.39675500	2.40754300	-2.62085700
H	-1.75528300	3.55114700	-2.39708800
Si	1.19733800	-0.22604100	-0.09295700
N	-0.03488300	-0.55939000	0.98251900
C	-0.16336100	-1.15458300	2.28835100
H	-0.93094300	-0.63061000	2.87666000
H	-0.46447500	-2.21403300	2.23116000
H	0.78612200	-1.09343500	2.84628600
N	2.52026600	-1.43040500	-0.48448100
C	2.66514700	-2.74529700	-1.04260600
H	1.67422600	-3.21967200	-1.05823500
H	3.33374100	-3.37677600	-0.43591100
H	3.04438200	-2.71508000	-2.07740200
C	3.44129900	-0.58899900	-0.02384600
C	4.90806300	-0.79546900	0.02176600
H	5.29365300	-0.54365300	1.02051500
H	5.18222100	-1.82648900	-0.23036300
H	5.39009900	-0.11343100	-0.69655900
N	2.79973600	0.50457100	0.39193900
C	3.35448200	1.70403200	0.95863800
H	3.95200300	1.48918400	1.86012900
H	3.99046700	2.24060900	0.23493200
H	2.52996800	2.37057500	1.24632800
O	0.25622800	0.38194000	-1.22167000
C	-0.19169000	3.18678900	0.41177100
O	0.47745100	3.54196000	1.30998800

VI.5.27. $CO_2\_TS_5^M$

0 1

Si	-1.44061700	-0.03477500	-0.09744800
O	-1.90813000	-1.05923400	-1.35466900
O	-2.59611600	-0.51723000	1.09124400
C	-1.56285400	-2.35997000	-1.33489500
H	-0.98712200	-2.68689700	-2.21085000
C	-1.89883100	-3.22463900	-0.35585600
H	-1.58854700	-4.26359500	-0.50278300
C	-2.69677400	-2.93648900	0.82697600
H	-3.13789100	-3.80272800	1.32837200
C	-3.00555600	-1.74792400	1.39845800

H	-3.67591700	-1.75111300	2.26756800
N	-1.92616500	1.61551000	-0.25997700
C	-3.32517900	1.99872100	-0.22902400
H	-3.54123200	2.67537400	0.61684400
H	-3.95428900	1.10655900	-0.11086300
H	-3.62648000	2.50857400	-1.16133100
C	-1.00063300	2.66837800	-0.47303900
C	-0.77227400	3.24876800	-1.83736300
H	-0.05156400	4.08009800	-1.81524600
H	-0.37691200	2.44541900	-2.47813100
H	-1.71316700	3.63121600	-2.26635200
Si	1.20342200	-0.10209400	-0.37909600
N	0.02489000	-0.42148800	0.79507900
C	0.10532300	-0.95196200	2.13772800
H	-0.64852400	-0.48789600	2.79047900
H	-0.06232400	-2.04231300	2.16590200
H	1.09587000	-0.74358800	2.57611300
N	2.53603600	-1.36742400	-0.52537800
C	2.69637500	-2.72893000	-0.94676700
H	1.70521900	-3.20364700	-0.96126500
H	3.33358000	-3.30207600	-0.25437400
H	3.12212900	-2.80115800	-1.96167200
C	3.41818700	-0.52669000	0.00404200
C	4.85496000	-0.78327800	0.27245400
H	5.09697000	-0.52418500	1.31373200
H	5.12318800	-1.82960800	0.08609800
H	5.46685400	-0.13796100	-0.37686100
N	2.77410900	0.61182200	0.25871200
C	3.29141500	1.82945700	0.81736900
H	4.01100100	1.62871500	1.62742200
H	3.78971600	2.45302400	0.05544100
H	2.45876600	2.40854300	1.24035200
O	0.34297700	0.41922500	-1.57153900
C	-0.33698900	3.08601200	0.59502600
O	0.28830600	3.41445700	1.53162900

VI.5.28. $CO_2\_I_5^M$

0 1

Si	0.90226300	-0.14013000	-0.47035000
O	0.67118100	-1.01698600	0.95002500
O	0.70389300	-1.19620300	-1.74728900
C	1.31039100	-2.17262600	1.23906100
C	1.33581800	-3.27072900	0.45991200
C	0.75305000	-3.44769400	-0.86173700
C	0.47784800	-2.51815300	-1.79952900
N	2.48487000	0.49008900	-0.58246800
C	3.50451800	0.10362500	-1.53714900
C	2.83975000	1.43809600	0.42041400
Si	-1.89779900	1.09651600	-0.21886800
N	-2.82219600	2.24399000	-0.72521500
C	-3.14660400	3.50191100	-1.26149300
N	-2.01337100	0.31558800	1.47786700
C	-1.50149800	0.58227600	2.78781400
C	-2.28430200	-0.86468800	0.93633900
N	-2.38186600	-0.68714400	-0.37997100
C	-2.86521200	-1.61810400	-1.35572900
O	-0.22088300	1.02397700	-0.46387800
C	2.40991500	2.68638200	0.24802500
O	2.01288000	3.76959300	0.11326500
C	3.66681400	1.03869600	1.60918000
H	3.86442600	1.89154300	2.27485200
H	3.14657300	0.26248300	2.19512700
H	4.64210700	0.62848300	1.29728300
C	-2.39928100	-2.16954800	1.63251500
H	-3.21676300	-2.76596600	1.20458100
H	-1.45810000	-2.72448500	1.48293100
H	-2.56567000	-2.04350500	2.70911700
H	-2.71228200	-1.18936000	-2.35486300
H	-2.31875200	-2.57499000	-1.31246800

H	-3.94497800	-1.81993700	-1.24298700
H	-0.40699100	0.44194600	2.82614000
H	-1.96156300	-0.07208600	3.54544400
H	-1.73120300	1.62184800	3.05988400
H	-3.61609600	4.17567400	-0.51467300
H	-3.87946800	3.42898900	-2.09177500
H	-2.27893100	4.06048100	-1.67059800
H	3.09055400	-0.63189500	-2.24029200
H	3.85701500	0.97400200	-2.11684100
H	4.37894100	-0.35417800	-1.04157100
H	1.79291500	-2.17428700	2.22310800
H	1.84086400	-4.13999100	0.89090300
H	0.54505400	-4.47713500	-1.16668300
H	0.05399000	-2.82443400	-2.76176400

VI.5.29. CO_2_TS_6^M

0 1

Si	-1.35985700	0.87738100	-0.09653200
O	-0.76781700	1.67753800	1.26942800
O	-1.88934700	2.04396200	-1.16104000
C	0.18218400	2.63203600	1.25559100
C	0.48452700	3.49880000	0.26686700
C	-0.12321400	3.69931200	-1.04308000
C	-1.15666400	3.07112800	-1.63800900
N	-2.60324000	-0.20280000	0.29631300
C	-4.00406700	0.07667500	0.51649900
C	-2.03357600	-1.48488000	0.59743200
Si	0.92129800	-1.14712800	-0.28919300
N	0.37905300	-2.66134800	-0.29805900
C	0.76395900	-3.93538600	-0.80199000
N	1.84862400	-0.43285700	1.15741500
C	1.72460700	-0.31249700	2.57937000
C	2.84161100	-0.07833600	0.35673000
N	2.56786600	-0.55148500	-0.86360600
C	3.13863000	-0.12405100	-2.10926000
O	-0.05170600	0.12090400	-0.74692200
C	-1.76615500	-2.21402900	-0.51741700
O	-1.96290700	-2.60913000	-1.60385800
C	-1.63379100	-1.85439900	1.99181300
H	-0.94570100	-2.71151000	1.96789400
H	-1.13108000	-1.01752400	2.50917900
H	-2.50153000	-2.13456600	2.61699000
C	4.01768500	0.75541400	0.71148600
H	4.89784100	0.46965000	0.12111800
H	3.77616900	1.80752800	0.48820000
H	4.25844100	0.67945300	1.77882300
H	2.98976100	-0.90808600	-2.86420000
H	2.66337300	0.80097300	-2.48048200
H	4.22221000	0.05033600	-2.01870800
H	0.84554300	0.30031900	2.83144400
H	2.61512300	0.15319800	3.02659300
H	1.58602100	-1.30521900	3.03516200
H	0.80431800	-4.70470400	-0.00619300
H	1.75048500	-3.95262100	-1.30594900
H	0.02593600	-4.29267600	-1.54874100
H	-4.23960400	1.09875300	0.18163900
H	-4.63313900	-0.62206400	-0.05990800
H	-4.28339300	-0.00886900	1.58211500
H	0.74061500	2.67876900	2.19773900
H	1.29767800	4.18763200	0.51270200
H	0.30847900	4.50792500	-1.63868000
H	-1.49404200	3.39428500	-2.62809500

VI.5.30. CO_2_3^M

0 1

Si	-1.64634000	-0.11142900	0.11621100
O	-2.46434500	0.67018800	1.34693900

O	-2.73625600	-0.29825700	-1.12759000
C	-3.06092700	1.87641800	1.19104200
C	-3.97629600	2.18625400	0.25570500
C	-4.50118200	1.33080200	-0.79922300
C	-3.94442100	0.24494700	-1.36878500
N	-0.98793100	-1.60797600	0.56971300
C	-1.71974900	-2.84805000	0.71295100
C	0.46722400	-1.60662000	0.74361100
Si	1.14925600	0.02685300	-0.08637000
N	1.71164700	-1.28607100	-1.13618800
C	2.42865100	-1.46851000	-2.36877200
N	2.23679800	0.91700700	1.10381200
C	2.64195900	0.83890400	2.47775100
C	2.67044300	1.79892100	0.18702300
N	2.13074800	1.46885200	-0.95789100
C	2.06604900	2.20694200	-2.17936500
O	-0.34153700	0.79179300	-0.34529200
C	1.15242300	-2.33253100	-0.43032600
O	1.20107900	-3.51343800	-0.69804000
C	0.90845100	-2.15074100	2.09027400
H	1.99916200	-2.06755700	2.21871800
H	0.41286800	-1.60896000	2.91158700
H	0.65767500	-3.22048800	2.18599900
C	3.57492900	2.94061600	0.48752300
H	3.86587500	3.48130900	-0.42050900
H	3.07246800	3.64126200	1.17236900
H	4.48142700	2.57795100	0.99415200
H	2.37131000	1.57501800	-3.02825700
H	1.02939500	2.53484300	-2.36444300
H	2.71418800	3.09762200	-2.16825800
H	1.88025500	0.29429600	3.04879100
H	2.73833700	1.84033300	2.92841700
H	3.60204600	0.30820500	2.61028900
H	3.39279400	-0.93677200	-2.35018100
H	1.85079300	-1.10809300	-3.23654100
H	2.61639600	-2.54436800	-2.50712800
H	-2.73603900	-2.72903500	0.30762100
H	-1.22397800	-3.65652300	0.15140300
H	-1.81338100	-3.16670000	1.76690100
H	-2.75701500	2.61312600	1.94342000
H	-4.39433300	3.19477700	0.32225100
H	-5.47609000	1.60955300	-1.20894000
H	-4.48066700	-0.29480700	-2.15615700

VII. References

1. S. S. Sen, H. W. Roesky, D. Stern, J. Henn, D. Stalke, High yield access to silylene. *J. Am. Chem. Soc.* **2010**, *132*, 1123-1126.

2. G. A. Sheldrick, short history of SHELX. *Acta Cryst. A* **2008**, *64*, 112-122.

3. G. A. Sheldrick, Crystal structure refinement with SHELXL. *Acta Cryst. C* **2015**, *71*, 3-8.

4. O. V. Dolomanov, L. J. Bourhis, R. J. Gildea, J. A. K. Howard, H. Puschmann, OLEX2: a complete structure solution, refinement, and analysis program. *J. Appl. Crystallogr*. **2009**, *42*, 339-341.

5. M. J. Frisch, G. W. Trucks, H. B. Schlegel, G. E. Scuseria, M. A. Robb, J. R. Cheeseman, G. Scalmani, V. Barone, G. A. Petersson, et al., *Gaussian 16, Revision B.01*, **2016**.
